# Reassessment of the roles of coronin proteins as actin effectors and in signaling

**DOI:** 10.1371/journal.pbio.3003904

**Published:** 2026-08-18

**Authors:** Roko Gvozdenica Šipić, Haiyan Zhang, Andrii Kharin, Jerneja Koren, Viviana D’Otolo, Yuko Nariai, Takeshi Urano, Pawel Pelczar, Klemens Fröhlich, Alexander Schmidt, Urszula Brykczynska Kunzmann, Tohnyui Ndinyanka Fabrice, Alexey Baldin, Jean Pieters

**Affiliations:** 1 Biozentrum, University of Basel, Basel, Switzerland; 2 Center for Vaccines and Therapeutic Antibodies for Emerging Infectious Diseases, Shimane University, Izumo, Japan; 3 Center for transgenic models, University of Basel, Basel, Switzerland; UCL: University College London, UNITED KINGDOM OF GREAT BRITAIN AND NORTHERN IRELAND

## Abstract

Coronin proteins are present in all known non-plant eukaryotes, and are involved in key biological processes including cytoskeletal dynamics and the regulation of cell population sizes in amoeba and mammals. How, exactly, coronin proteins exert their function is debated. On the one hand, coronins are widely considered as F-actin-binding and regulatory proteins. On the other hand, coronin proteins were also shown to act as regulators of cAMP/Ca^2+^ signaling. Here, we demonstrate that endogenously expressed mammalian coronin 1, 2 and 3 did not depend on F-actin for their subcellular localization and did not impact actin-related processes that were previously reported to be coronin-dependent. We also show that the fusion of GFP or FLAG-tags to coronin proteins resulted in their increased colocalization with actin and phenocopied loss-of-function mutation in mice. Together these results suggest that inclusion of tags on coronin proteins causes increased colocalization with F-actin and can disrupt their in vivo function. Furthermore, we show that the ubiquitously expressed coronin 2 and coronin 3 regulated cAMP production regardless of the presence of F-actin. The analysis presented here may contribute to a re-evaluation of the function of coronin proteins in signaling, independently of actin modulation.

## Abstract 2

Coronins are evolutionarily conserved proteins involved in many biological processes, but exactly how coronin proteins exert their function is unclear. This study shows that peptide or fluorescent protein tagging has the potential to alter coronin colocalization with F-actin and disrupt their in vivo function, with further evidence of coronin involvement in cAMP signalling.

## Introduction

Members of the coronin protein family are highly conserved throughout evolution and expressed in most eukaryotes, with the exception of plants. In metazoa, there is an expansion of the gene family with mammals expressing up to 7 coronins [1–3]. Structurally, coronin proteins consist of a central tryptophan-aspartate (WD)-containing domain (also referred to as WD40 repeats) that folds into a β-propeller structure followed by a C-terminal coiled coil that mediates oligomerisation [4–6]. Coronin proteins are localized in the cytosol and are partially associated with membranes [5,7–10]. Their reported activities, which range from modulation of the actin cytoskeleton to signaling, have a wide-ranging impact on a plethora of biological processes including immunity, development, cell survival and cell population size control, at least some of which are evolutionarily conserved from amoeba to humans [11–15].

Coronin was initially detected in a search for components possibly involved in chemotaxis in the slime mould *Dictyostelium discoideum.* In particular, a link was sought between environmental sensing and cytoskeletal regulation, the latter known to be involved in chemotaxis [16]. Analysis of the proteins co-precipitating with an actin/myosin complex identified coronin, so named because of its localization in the crown-shaped cell surface projections typical of *Dictyostelium discoideum* [4]. Subsequent analysis of *Dictyostelium* lacking coronin (now referred to as coronin A, given the presence of a second, non-redundant ‘tandem’ coronin, coronin B) showed reduced growth and migration as compared to wild-type cells whereas crown formation, actin assembly, chemotaxis and phagocytosis or localization of actin filaments were not affected by coronin deletion [4,17–19]. In the yeast *Saccharomyces cerevisiae*, the coronin-like protein, termed Crn1, was found to localize to cortical actin patches whereas deletion of *crn1* does not affect polarized growth and secretion, endocytosis, mating projection formation and bud side selection, suggesting Crn1 to be dispensable for these F-actin-dependent processes in yeast [20,21]. Further, in mammalian cells, the loss of coronin 1, one of the most widely studied coronin proteins, does not affect migration, phagocytosis, macropinocytosis, proliferation and F-actin distribution and dynamics [22–27]. Thus, while the initial detection of coronin in *Dictyostelium* occurred as a result of analyzing actin/myosin-interacting proteins, a number of subsequent analyses of phenotypes in amoeba, yeast and mammalian cells suggested that the investigated cellular processes regulated by F-actin are unaffected upon inactivation of coronin-encoding genes.

Apart from the analysis of a role for coronin proteins in processes that are known to depend on F-actin, several studies in amoeba and mammalian cells have implicated coronin proteins in the regulation of cAMP and Ca^2+^-dependent signaling. For example, *Dictyostelium* coronin A was found to be required for the initiation of the cAMP relay leading to the transition from single-celled amoeba to multicellular fruiting body formation [18]. In mammals, coronin 1-dependent modulation of cAMP and Ca^2+^ signaling underlies the intracellular survival of *M. tuberculosis* [24,25], cognition and behavior [27,28] and the regulation of T cell population size [13,22,23,29,30]. Of note, both in *Dictyostelium* and mammalian cells, coronin-dependent cAMP signaling is unaffected upon F-actin depolymerization [18,27]. A role for coronin 1 and coronin A in the regulation of cAMP signaling is furthermore consistent with structural homology of coronin proteins to the β subunit of trimeric G proteins known to be involved in cAMP signaling [4–6,18,24,29].

Thus, the exact molecular function of coronin proteins has been a matter of debate, with a considerable number of studies suggesting a direct function for coronin proteins in F-actin-mediated cytoskeletal modulation (see, e.g., [12,31–33], whereas other studies suggest coronin proteins to regulate cAMP/Ca^2+^-dependent signal transduction in a manner that is independent of F-actin [18,22,24,26–30,34].

In this report, as an attempt towards a better understanding of the biology of coronin proteins, we present a systematic analysis of mammalian coronin 1, 2 and 3 regarding (i) the subcellular localization of endogenously expressed coronin proteins in relation to actin, (ii) the consequences of tagging coronin proteins with green fluorescent protein (GFP) and FLAG tags [35–37] for their subcellular localization and in vivo functionality and (iii) the deregulation of F-actin dynamics upon their absence in mammalian cells. In the course of this work, we found that most of the available antibodies, including ones reported in the literature for detecting coronin 2 or coronin 3 localization, show no differential staining between wild-type and coronin-deficient cells. Furthermore, GFP-tagging of coronin proteins at the N- or C-termini significantly increased their colocalization with F-actin in cultured cells while N-terminal FLAG-tagging of coronin 1 phenocopied coronin 1 deficiency in transgenic mice. We also found that actin polymerization dynamics were not perturbed in cells upon the abrogation of coronin protein expression. Additionally, proteomic and transcriptomic analyses of wild-type and coronin-deficient cells and mice revealed no significant differences in expression of proteins previously implicated in coronin-mediated F-actin regulation. Similarly, we found that the bulk of coronin proteins does not associate with filamentous actin in cells. Moreover, bibliometric analysis suggested that conclusions on coronin-mediated effects on the F-actin cytoskeleton are to a large extent based on the analysis of heterologous experimental systems that may not replicate the function of native coronin proteins within cells. Finally, we report that the most widely expressed mammalian coronin proteins, namely coronin 2 and coronin 3, were required for stimulus-dependent cAMP production regardless of the presence of F-actin.

Together the results reported here suggest that tagging coronin proteins can result in their mislocalization in cells and impede their functionality in vivo. Combined with both published and here-provided evidence for functions of coronin proteins within cells in the absence of F-actin, these results may lead to a re-evaluation of non-F-actin-related functions for coronin proteins, including in cAMP signaling.

## Results

### Identification of coronin 2 and 3 specific antibodies

A common approach when analyzing the intracellular localization of coronin proteins is to ectopically express them as fusions with different fluorescent proteins or other tags (see S1 Table). Given that inclusion of a tag may alter protein function and/or localization [38–40], we decided to compare the localization of endogenously expressed coronin 1, 2 and 3 with that of their respective GFP-tagged counterparts. In order to do so, we first evaluated the availability of antibodies specifically recognizing the individual coronin proteins.

A well-accepted standard for deciding on antibody specificity when performing immunolocalization analyses on fixed cells is to test reactivity on the same specimen lacking the antigen [41,42]. To analyze the specificity of antibodies for detection of coronin 2 or 3, we generated coronin 2, coronin 3, as well as coronin 2- and 3-deficient HeLa cell lines using CRISPR/Cas9 technology (S1A and S1B Fig). HeLa cells, that are of epithelial origin [43], express predominantly coronin 2 and 3 (S1C Fig). Subsequent testing of multiple antibodies, including ones cited in the literature as specific for coronin 2 and 3 detection (either by immunostaining on fixed cells or tissue sections) showed a high degree of nonspecific staining in wild-type and coronin 2- or coronin 3-deficient cells (S2 Table), suggesting the antibodies not to be sufficiently specific under the conditions tested. Our findings thus show that the majority of the tested antibodies fail to distinguish between wild-type and coronin 2- and 3-deficient cells when analyzed by immunofluorescence staining on fixed cells.

For specific detection of coronin 2 using paraformaldehyde (PFA) fixation, that is compatible with phalloidin-mediated detection of F-actin [44,45], a mouse monoclonal IgG2bκ, was identified that specifically detected coronin 2 in wild type, but not in coronin 2-deficient HeLa cells (S2 Table). Using this mouse monoclonal antibody, coronin 2 was found to localize in puncta distributed throughout the cytoplasm of HeLa cells (S1D Fig). Also, consistent with earlier work [46], analysis of the localization of coronin 2 in murine embryonal fibroblasts (MEF) using the rabbit polyclonal antibody PA5-64443 (that is largely specific in HeLa cells apart from nonspecific signal in the Golgi area, see S2 Table) revealed a similar ubiquitous and punctate distribution in these untransformed primary cells (S2A Fig).

For coronin 3, none of the antibodies tested showed specific staining using PFA fixation (S2 Table). We therefore tested the same panel of anti-coronin 3 antibodies using methanol fixation, and identified a polyclonal rabbit antibody (PA5-30479) that specifically detected coronin 3 in wild-type but not coronin 3-deficient HeLa cells (S1E Fig), showing coronin 3 to localize throughout the cytoplasm with a fraction of the protein localizing to the plasma membrane.

### Relocalization of coronin proteins to F-actin upon GFP-tagging

Next, we analyzed the subcellular localization of endogenous coronin 1, 2 and 3 and their respective mEmerald fluorescent fusion variants relative to the actin cytoskeleton. mEmerald is a monomeric variant of EGFP [35], and referred to as GFP in the following text. The fluorescent fusion proteins were expressed ectopically by transiently transfecting cells with expression plasmids. As coronin 1 is primarily expressed in cells of hematopoietic origin [47,48], Jurkat T cells were used as a model to study its localization. To statistically analyze colocalization between tagged and untagged coronin proteins and actin, Pearson’s correlation coefficient was chosen to better account for different expression and background levels between conditions, as Manders’ coefficients heavily depend on appropriate thresholding and comparable levels of the signal between conditions, while the Pearson’s correlation coefficient requires no particular pre-processing of the images [49, 50]. Moreover, to accurately assess colocalization and account for the effects of antibody accessibility in fixed cells [51,52], both endogenous and tagged coronin protein variants were probed using antibody staining, without the reliance on GFP fluorescence.

Colocalization analysis showed that the addition of the GFP-tag onto coronin 1 with subsequent ectopic expression significantly increased its colocalization with F-actin relative to endogenously expressed, untagged coronin 1 (Figs 1A, 1C, and S3A). To better visualize the localization of coronin 1 or GFP-coronin 1 relative to F-actin, in a separate experiment, Jurkat cells were centrifuged onto coverslips prior to fixation and staining, thereby enlarging the cytoplasm:nucleus ratio. As shown in Fig 1B, this procedure resulted in visible separation of coronin 1 from F-actin in flattened cells, while GFP-coronin 1 still colocalized with F-actin to a high degree. It should be noted, however, that direct interaction between GFP-coronin 1 and actin cannot be ascertained, but can be excluded for endogenous coronin 1 at the resolution used. These results suggest that the exclusive use of GFP-coronin 1 in colocalization studies may lead to significantly overestimated levels of colocalization between coronin 1 and F-actin.

**Fig 1:**
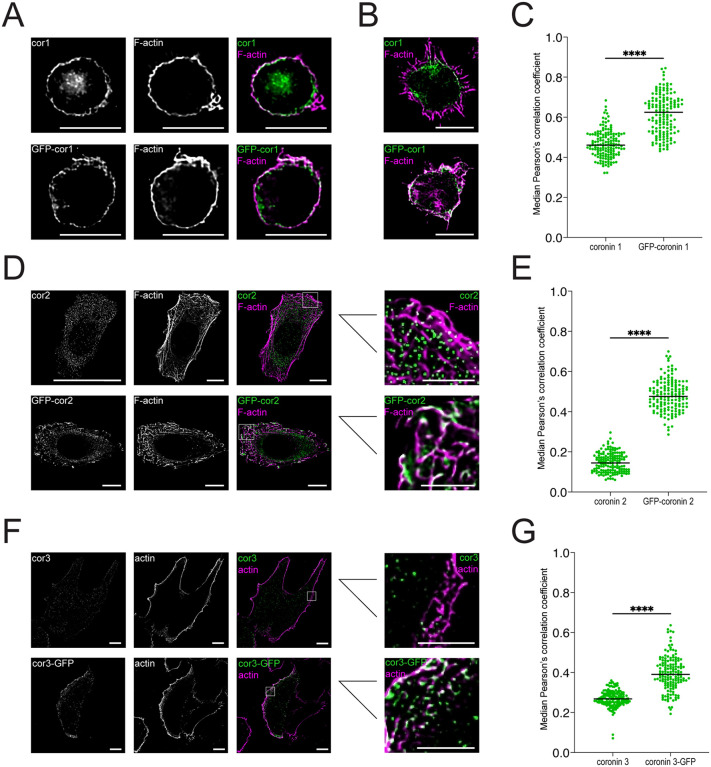
Relocalization of coronin proteins to F-actin upon GFP-tagging. **(A)** Representative Jurkat cells fixed in PFA, permeabilized, and stained for endogenously expressed coronin 1 (upper panels), or Jurkat cells transfected with a plasmid encoding GFP-coronin 1 fusion protein and then fixed in PFA, permeabilised, and stained with anti-GFP antibodies (lower panels). 647I-phalloidin was used to label F-actin. Images were deconvolved and single slices are shown. Bars are 10 μm. **(B)** Representative untransfected and transfected Jurkat cells seeded by centrifugation at 200*g* fixed in PFA, permeabilized and stained for either coronin 1 (upper) or GFP (lower) using antibodies, with F-actin stained using 647I-phalloidin. Images were deconvolved and single slices are shown. Bars are 10 μm. **(C)** Median Pearson’s correlation coefficients calculated between coronin 1 and coronin 1-GFP fusion proteins relative to F-actin in cells seeded by sedimentation and stained as in **(A)**. **** *p* < 0.0001 (Student *t t*est relative to coronin 1). Numeric values can be found in S1 Data. **(D)** Representative HeLa cells either fixed in PFA, permeabilized, and stained for endogenously expressed coronin 2 (upper panels), or HeLa cells transfected with a plasmid encoding GFP-coronin 2 fusion protein fixed in PFA, permeabilized, and stained with anti-GFP antibodies (lower panels). 647I-phalloidin was used to label F-actin. Images were deconvolved and single slices are shown. Enlarged insets from white rectangles are shown to the right of each image. Bars are 10 μm for images and 5 μm for insets. **(E)** Median Pearson’s correlation coefficients calculated between coronin 2 and GFP-coronin 2 fusion proteins relative to F-actin in cells stained as in **(D)**. **** *p* < 0.0001 (Student t tes*t* relative to coronin 2). Numeric values can be found in S1 Data. **(F)** Representative HeLa cells either fixed in methanol and stained for endogenously expressed coronin 3 (upper panels), or HeLa cells transfected with a plasmid encoding coronin 3-GFP fusion protein fixed in methanol and stained with anti-GFP antibodies (lower panels). Anti-actin antibodies were used to label actin. Images were deconvolved and single slices are shown. Enlarged insets from white rectangles are shown to the right of each image. Bars are 10 μm for images and 5 μm for insets. **(G)** Median Pearson’s correlation coefficients calculated between coronin 3 and coronin 3-GFP fusion protein relative to actin in cells stained as in **(F)**. **** *p* < 0.0001 Student t test. Numeric values can be found in S1 Data.

To characterize the localization of coronin 1 in an adherent cell line and account for the potential effect of tagged coronin overexpression on localization, we repeated the colocalization analysis in human melanoma Mel JuSo cells stably overexpressing either murine coronin 1 [48] or coronin 1-EGFP (not mEmerald). As seen in S4 Fig, coronin 1-EGFP colocalized with F-actin to a significantly higher degree relative to the untagged coronin 1, despite the fact that both coronin 1 and coronin 1-EGFP are not expressed in wild-type Mel JuSo cells. This suggests the properties of GFP or the effect of tagging by GFP that results in increased F-actin colocalization between tagged coronins and F-actin to be independently of or in addition to potential overexpression artifacts.

Colocalization of coronin 2 with F-actin in HeLa cells was done analogously to coronin 1 in Jurkat cells. Endogenously expressed coronin 2 and 3 localization was largely independent of actin, while fusion of coronin 2 or 3 to GFP with subsequent ectopic expression altered their localization and significantly increased colocalization with phalloidin-probed F-actin for coronin 2 (Figs 1D, 1E, S3B, and S3F) and with antibody-probed actin for coronin 3 (Fig 1F and 1G).

To ascertain a baseline of colocalization to be expected from abundant cytoplasmic or membrane-associated proteins that are considered not to bind actin, we used fluorescent wheat germ agglutinin (WGA) as a cellular membrane marker [53] and antibody-stained S6 ribosomal protein as a cytoplasmic marker (S3C and S3D Fig). Colocalization analysis of these membrane and cytoplasmic markers with F-actin showed that in Jurkat T cells the membrane marker colocalized with F-actin to a similar degree as coronin 1, while the cytoplasmic control stain showed lower colocalization with F-actin (S3E Fig), suggesting that the partial colocalization of endogenous coronin 1 with F-actin at the resolution used can be explained by its reported membrane association [5,8,54]. Notably, in HeLa cells, the colocalization coefficients for endogenous coronin 2 relative to F-actin were significantly lower even when compared to the coefficients obtained by colocalising the cytoplasmic (ribosomal) and membrane (wheat germ agglutinin) markers with F-actin (S3F Fig), suggesting the localization of endogenous coronin 2 to be independent of actin filaments. Interestingly, in cells ectopically expressing GFP stained for endogenous coronin 2, prominent actin filaments were largely positive for GFP fluorescence, but not antibody-probed coronin 2, suggesting the increased colocalization of fluorescently tagged proteins with the actin cytoskeleton to be a consequence of either the ectopic expression and/or properties of the GFP and GFP-fused protein, but not coronin 2 itself (S3F and S3G Fig). In fact, this observation is in line with previously published imaging data in the coronin literature showing enrichment of control GFP fluorescence along actin filaments [55] and is furthermore consistent with the reported aberrant localization of GFP-fused proteins and/or the interference of GFP with cellular processes, including F-actin colocalization and dynamics (see S2B Fig and [56–59]). Whereas partial colocalization of endogenous coronin 2 with actin filaments was observed in MEFs (S2A Fig), and more prominently in larger, flatter cells, transiently expressed GFP was also noted to follow similar filaments (S2B Fig), suggesting this manner of colocalization with F-actin to be a consequence of cell morphology and/or the experimental procedure used, but not necessarily to be of physiological relevance.

**Fig 2:**
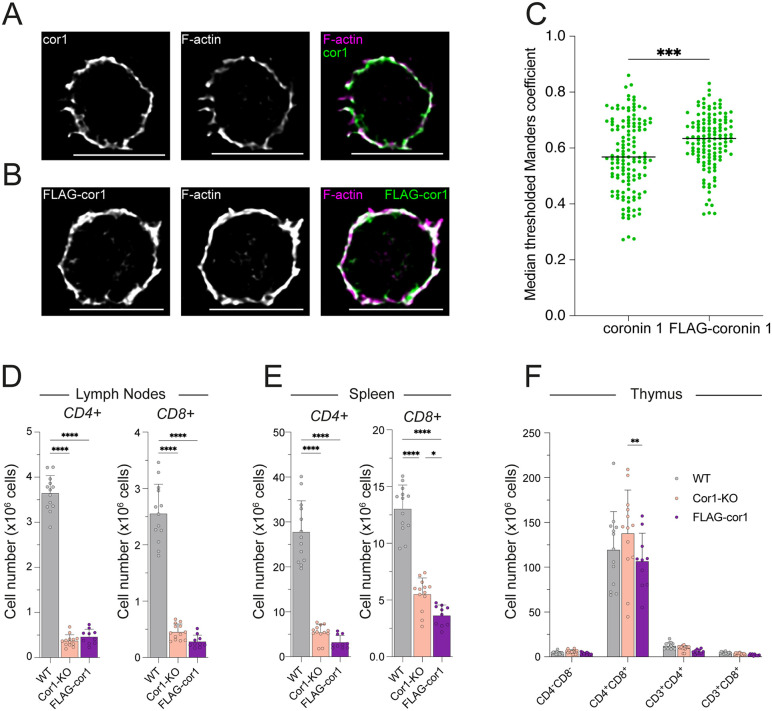
Knocking in the octapeptide FLAG tag at the N-terminus of coronin 1 in mice causes increased colocalization with F-actin and phenocopies coronin 1 deletion. **(A, B)** T cells were isolated from wild-type (A) or FLAG-KI (B) mice, fixed in PFA, permeabilized and stained for coronin 1 or FLAG-coronin 1 using anti-coronin 1 antibodies and F-actin using 647I-phalloidin. Samples were imaged on a super-resolution Zeiss Lattice SIM 3D microscope and deconvolved, with representative cells shown. Scale bar is 5 μm. **(C)** T cells were fixed and stained as in (A, B) and imaged using a Zeiss Cell Observer widefield microscope and deconvolved. Median thresholded Manders correlation coefficients (tM2) were calculated for coronin 1 and FLAG-coronin 1 relative to F-actin. *** *p* < 0.001 (Student *t t*est). Numeric values can be found in S1 Data. **(D, E)** Mean CD4+ (left panels) or CD8+ (right panels) T cell numbers in lymph nodes (D) and spleen (E) from wild type (WT), coronin 1-deficient (Cor1-KO) and FLAG KI (FLAG-cor1) mice. Each dot represents T cell numbers from one mouse. Error bars show standard deviation. Significance was assessed by one-way ANOVA. ****p < 0.0001, *p < 0.05. Numeric values can be found in S1 Data. Gating strategy and.fcs files can be found in S1 Data. **(F)** Mean double negative (CD4-CD8-), double positive (CD4+ CD8+) and single positive (CD3+ CD4+ and CD3+ CD8+) cells isolated from the thymi of the mice of indicated genotypes. Each dot represents cell numbers from one mouse. Error bars show standard deviation. Significance was assessed by two-way ANOVA. **p < 0.01. Numeric values can be found in S1 Data. Gating strategy and.fcs files can be found in S1 Data.

**Fig 3:**
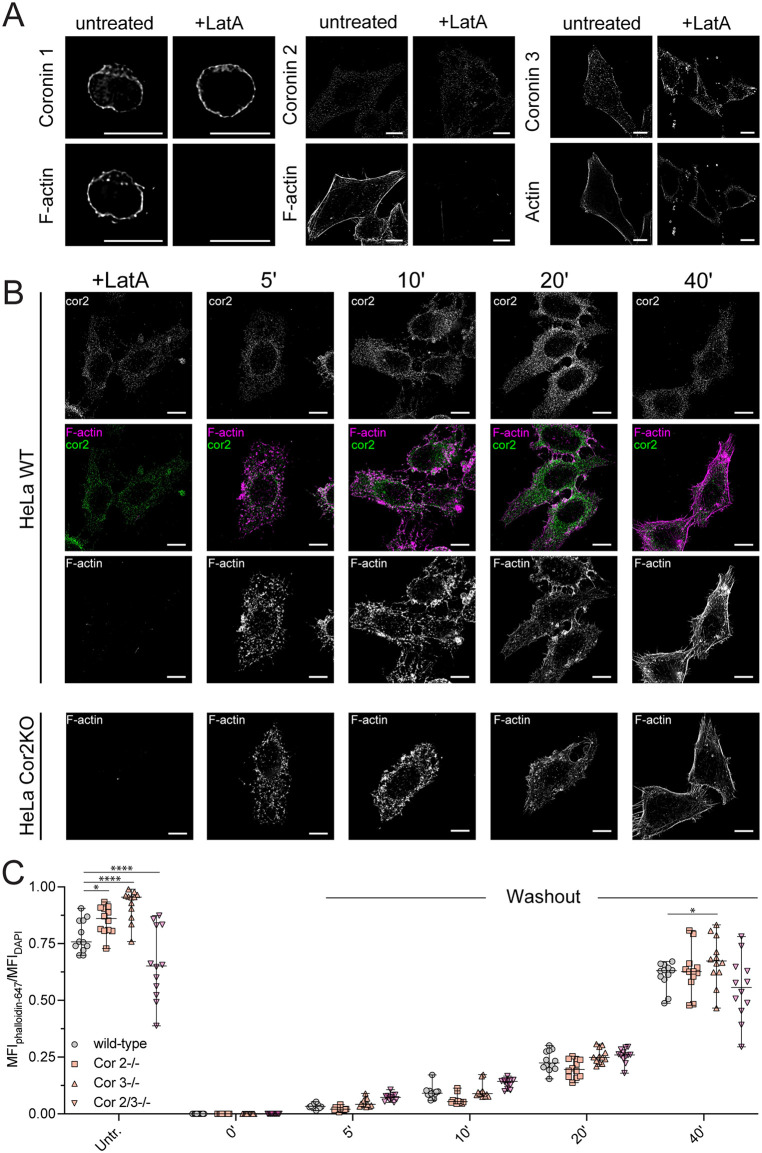
Endogenous coronin protein localization upon F-actin depolymerization and F-actin reassembly. **(A)** Representative images of Jurkat (left panels) and HeLa cells (middle and right panels) left untreated or treated with latrunculin A (1 h), fixed and stained with anti-coronin 1 (left panels, PFA), anti-coronin 2 (middle panels, PFA) or anti-coronin 3 (right panels, MeOH) antibodies, as well as 647I-labeled phalloidin (left, middle) or anti-actin antibodies (right). Bars are 10 µm. **(B)** Wild-type HeLa cells were treated with latrunculin A (1 h), after which the latrunculin A-containing medium was replaced with full cell culture medium without latrunculin A. Cells were fixed at the indicated timepoints and stained using anti-coronin 2 antibodies and 647I-phalloidin. F-actin stain of coronin 2-deficient HeLa cells (Cor2KO) cells treated the same way is shown underneath. Images were deconvolved and maximum intensity projections of 10 central slices are shown. Bars are 10 µm. **(C)** Mean fluorescence intensity (MFI) ratio of 647I-phalloidin to DAPI was quantified at the indicated timepoints following LatA treatment (2 h), washout, PFA fixation and staining for indicated HeLa cell lines. For MFI calculation, 12 replicate wells of 6 separate 96-well plates were measured per condition (*n* = 12) using a Tecan Spark plate reader. Lines indicate median with range. MFI ratio values relative to wild-type were compared using two-way ANOVA, * *p* < 0.05, **** *p* < 0.001. Numeric values can be found in S1 Data.

Together these results suggest that ectopic expression of coronin proteins fused to GFP or GFP-derivatives alters their subcellular localization relative to untagged and endogenously expressed coronin proteins in favor of increased colocalization with the F-actin cytoskeleton, possibly resulting in overestimation of the degree of colocalization between coronin proteins and actin.

### Insertion of the N-terminal octapeptide FLAG tag in the endogenous coronin 1-encoding locus increases F-actin colocalization and phenocopies coronin 1-deletion

The data thus far suggest that ectopic expression of coronin 1, 2 or 3 fused to a GFP-based tag resulted in their mislocalization and increased colocalization with the actin cytoskeleton. Indeed, the addition of fluorescent tags onto proteins has been reported to result in mislocalization, aberrant cytoskeletal structure, altered protein interaction dynamics and reduced cellular viability [39,40,60–62]. We therefore sought to investigate the functional consequences of fusing coronin 1 to the FLAG tag, which represents one of the smallest protein tags available and is considered not to affect the properties of the protein it is fused to [37,63]. To that end, and to ensure endogenous expression levels, a FLAG tag was inserted into the mouse coronin 1-encoding gene using CRISPR/Cas9 technology to generate N-terminally-tagged FLAG-coronin 1 knock-in mice (S5 Fig). Mice expressing the N-terminally inserted FLAG tag were viable, fertile and did not show any obvious phenotype. Analysis of the subcellular localization of knocked-in FLAG-coronin 1 relative to F-actin in T cells showed a small but significant increase in F-actin colocalization compared to wild type T cells expressing untagged coronin 1 as estimated using the thresholded Manders’ coefficient (tM2) to express the fraction of above-background coronin 1 tagged or untagged variant on F-actin-positive structures (Fig 2A–2C). Manders’ coefficient was chosen over Pearson’s correlation coefficient for this particular analysis based on the endogenous expression levels of both tagged and untagged proteins, thus allowing for robust thresholding of the signal. As such, Manders’ coefficient allows for more biologically meaningful interpretation of colocalization, i.e., to state what fraction of total signal of the probe of interest colocalizes with the second probe, in this case coronin 1, with F-actin [49,50]. These results suggest that the presence of the short peptide FLAG-tag can affect the localization of coronin 1 relative to F-actin.

Given the in vivo role for coronin 1 in the establishment of the appropriate T cell population size [13,22,23,30,33,64–66], we investigated the consequence of expressing FLAG-tagged coronin 1 on T cell populations in secondary lymphoid organs. We found that the presence of the FLAG tag at the N-terminus of coronin 1 resulted in a significant reduction of T cell subsets in peripheral lymphoid organs of FLAG knock-in mice as compared to wild type mice, while T cell population sizes in the thymus were similar (Fig 2D–2F). The profound reduction in T cell numbers in peripheral lymphoid organs as observed following N-terminal FLAG insertion phenocopied the reduction of T cell numbers upon coronin 1 deletion [22,23,33,64,65], despite the expression of FLAG-coronin 1 being comparable to the expression of untagged coronin 1 in wild type mice (S5D and S5E Fig). We therefore conclude that fusion of the N-terminal FLAG octapeptide tag to coronin 1 results in disruption of its function in T cell population size control in mice, thus phenocopying genetic ablation of coronin 1.

### Coronin localization is independent of the presence of F-actin

The above results suggest that fluorescent protein or peptide tagging of coronin proteins can cause their increased colocalization with the F-actin cytoskeleton and may result in disruption of their in vivo activity in the case of FLAG-coronin 1. Whether or not F-actin dynamics affect endogenous coronin localization in cells is not clear. To address a possible role of the F-actin cytoskeleton in the localization of endogenously expressed, non-tagged coronin proteins, we subjected cells to the G-actin-sequestering agent latrunculin A (LatA) that is commonly used to depolymerize F-actin in living cells [67]. As can be seen in Fig 3A, the presence of LatA resulted in profound depolymerization of F-actin as judged by the absence of most of the fluorophore-coupled phalloidin fluorescence. As expected, signal corresponding to monomeric actin was still detectable by anti-actin antibody staining in methanol-fixed samples (in the case of coronin 3 detection, Fig 3A). Crucially, the subcellular localization pattern of coronin 1, 2 and 3 remained essentially unaltered.

We previously reported that the localization of coronin 1 in macrophages can be shifted from the cellular cortex to the cytoplasm upon phosphorylation due to interferon-γ/protein kinase C (PKC) signaling [68,69]. To analyze whether this relocalization of coronin 1 was dependent on the presence of F-actin, we stimulated J774.A1 murine macrophages, as well as Jurkat T cells, with phorbol myristate acetate (PMA), a potent PKC agonist [70], in the presence or absence of LatA. As shown in S6A and S6B Fig, cortical coronin 1 localization in both cell lines was not altered upon depolymerization of the actin cytoskeleton, whereas a reduction in cortical staining and a more diffuse cytoplasmic signal were observed upon PMA treatment independently of the presence of an intact F-actin cytoskeleton. A similar reduction in the cortical stain was observed using the protein tyrosine phosphatase inhibitor pervanadate ([71], see lower panels in S6A and S6B Fig). Since coronin 2 shows mostly cytoplasmic localization in HeLa cells and MEFs (Figs 1D, S1D, and S2A), we hypothesized that an opposite effect may be observed upon inhibition of PKC activity. To this end, we treated HeLa cells with the PKC inhibitor chelerythrine [72], as well as PMA. The localization of coronin 2 remained largely unaltered by PMA treatment, while chelerythrine treatment resulted in prominent relocalization of coronin 2 onto the cell cortex, independently of the presence of an intact F-actin cytoskeleton (S6C Fig).

Taken together, these results suggest that filamentous actin is dispensable for the subcellular localization as well as stimulus-dependent relocation of endogenous coronin proteins.

### Coronin depletion does not affect F-actin dynamics

While the above results suggest that coronin localization is independent of F-actin, whether the absence of coronin proteins alters actin polymerization dynamics in cells is not clear. To further analyze a potential function of coronin 2 and coronin 3 in F-actin polymerization, wild-type HeLa cells or cells lacking coronin 2 and/or 3 were treated with latrunculin A to depolymerize F-actin, followed by re-culturing in medium lacking latrunculin A to allow actin repolymerization. As shown in Fig 3B, during actin repolymerization, the subcellular localization pattern of endogenous coronin 2 remained essentially unchanged, and no obvious recruitment of endogenous coronin 2 to sites of actin nucleation and repolymerization was observed. Furthermore, no apparent morphological differences in the F-actin cytoskeleton were observed during the actin repolymerization process in coronin 2-deficient cells subjected to the same treatment (Fig 3B). Moreover, actin repolymerization dynamics were similar between wild-type and coronin 2- and/or coronin 3-deficient cells (Fig 3C), suggesting F-actin polymerization dynamics in cells to be independent of coronin 2 or 3. Also, since no other coronin proteins detected by mass spectrometry in four different clones of coronin 2 and 3-deficient HeLa cells showed a compensatory increase (S7 Fig), we conclude that the lack of effect on actin repolymerization dynamics upon the deletion of coronin 2 and/or 3 cannot be due to compensation by other coronins.

Together these data suggest that coronin 2 and 3 are dispensable for actin polymerization in cells.

### Biochemical characterization of coronin protein association with filamentous actin in cells

To experimentally test whether, and if so, to what degree endogenous coronin proteins associate, either directly or indirectly, with filamentous actin in cells, we performed pelleting assays as described previously [19,73]. In these assays, to increase the intracellular ratio of filamentous-to-globular actin in living cells, we employed the toxin jasplakinolide that causes an increased polymerization state of actin by stabilizing its filaments [74]. Conversely, to achieve the opposite effect and depolymerize filamentous actin, we used LatA. As shown in Figs 4A, 4B, S8A, and S8B, filamentous and globular actin were efficiently separated using the described method, as well as predominantly shifted into the pellet or supernatant fraction using jasplakinolide or latrunculin A treatment, respectively. However, supernatant-to-pellet distribution of endogenously expressed coronin 1 in Jurkat (Fig 4A), as well as coronin 2 and 3 in HeLa cells (Fig 4B) remained unaltered regardless of the polymerization state of actin in cells. To verify that the assay can capture conventional known F-actin binders, the same lysates were probed for the distribution of the Arp2 component of the Arp2/3 complex [75,76], showing that Arp2 distribution pattern closely matched the distribution pattern of filamentous actin (Fig 4A and 4B). To compare whether, and if so, to what degree, the tagging of coronin proteins by GFP affects the association of coronin proteins with actin in the here-employed pelleting assay, we analyzed cells ectopically expressing N- and C-terminal fusions of GFP to coronin 1 in Jurkat and to coronin 2 in HeLa cells revealing no apparent differences in the supernatant-to-pellet ratios. As expected, all immunoreactivity of GFP was recovered in the supernatant regardless of the treatment (S8A and S8B Fig).

Taken together, these results suggest that the increase in colocalization of GFP-fused coronins with actin is unlikely to be due to an increase in F-actin binding and that the bulk of endogenous coronins 1, 2 and 3 does not associate with F-actin under the conditions tested.

### Coronin 1, 2 or 3 ablation does not result in deregulation of actin-interacting and modulating proteins previously reported as coronin-dependent

To further evaluate the reported involvement of coronin proteins in actin regulation, we analyzed the effect of coronin ablation on the expression of proteins involved in a number of actin-related processes. To this end, we assembled a list of 53 proteins that are reported to be regulated by or interact with coronin 1, 2 or 3 in the context of actin-mediated cytoskeletal modulation (20, 40 and 30 proteins respectively listed in S3 Table and annotated for the described link to coronins in S4 Table), including among others Arp2/3 complex components, cofilin, actin isoforms and actin-interacting protein 1 (WDR1) (S3 and S4 Tables). We examined the differential abundance of these proteins in corresponding proteomes of wild-type versus coronin 1-deficient Jurkat cells [13] and in wild-type versus coronin 2- or 3-deficient HeLa cells (Fig 5A and S2 Data). When applying the standard significance thresholds of adjusted *p*-value below 0.01 and fold change above 2, with an exception of smooth muscle actin isoform ACTH which was upregulated in coronin 3-deficient HeLa cells (see also below), there were no changes in the abundance of the analyzed proteins.

The transcriptomes of wild-type versus coronin 1- or 3-deficient Jurkat cells and of wild-type versus coronin 1-deficient mouse T cells [30] were also analyzed in a similar manner (Fig 5B and S2 Data). By applying the same standard thresholds (adj. *p*-value <0.01 and fold change >2), no significant changes in the expression of the majority of the analyzed actin-related genes were detected. The exception to this was the observed upregulation of ACTG2/Actg2, encoding the smooth muscle actin isoform ACTH, that was also upregulated in coronin 3-deficient HeLa cells (see above and Fig 5A) that, interestingly, was reported to be deregulated in autoreactive T cells [77]. Furthermore, whereas a slight upregulation of the Actin-related protein 3B (Actr3b) and Src substrate cortactin (Cttn) was detected in coronin 1-deficient mouse T cells, expression of the main 7 components of the Arp2/3 complex was not changed. The analysis presented here suggests that upon coronin 1, 2 or 3 ablation the proteome and transcriptome of actin-interacting and modulating proteins, previously reported to interact with or be regulated by these coronin proteins, were not deregulated.

### Bibliometric analysis on coronin proteins and F-actin binding and modulation

The above data suggest that endogenously expressed coronin proteins largely do not associate with F-actin in cells nor were genes or proteins reported to be associated with coronins deregulated in their absence. These results are in apparent contrast to the frequent description of coronin proteins primarily as being actin-binding and -modulating proteins in the existing literature. To gain a better understanding of the available data supporting the association and/or modulation of actin by coronin proteins, we analyzed the collective literature on coronin proteins for reports on coronin-actin interaction (see S3 and S4 Data and https://www.coronin.org).

Analysis of the available literature on coronin proteins revealed that the majority of studies do not address coronin-actin interaction experimentally, instead referring to previous work stating F-actin interaction and modulation by coronin proteins (gray bars in Fig 6A–6C and S3 and S4 Data). The bibliometric analysis also showed that in non-mammalian eukaryotes, the majority of studies concluding on interaction of coronin proteins with actin are conducted studying the yeast coronin-like protein Crn1 (see red bar in Fig 6A and S3 Data), that, in contrast to non-fungal coronin proteins, possesses several domains predicted to interact with actin-binding and -modulating proteins [1,2,78,79]. Also, the conclusions of Crn1 interacting with actin in these studies are largely based on experiments with *E. coli*-expressed Crn1 fusion proteins using heterologous systems where such purified Crn1 constructs are mixed with rabbit muscle actin [79,80]. Such in vitro assays may not reflect the physiological conditions occurring in the cytoplasm of a living cell. Specifically, the reported higher affinity of yeast Crn1 for rabbit muscle actin compared with yeast actin [81] suggests that the choice of such heterologous experimental setup may contribute to the risk of observing non-physiological phenomena.

### Regulation of cAMP production by coronin proteins

The results thus far show that the bulk of endogenous coronin proteins did not associate with F-actin in cells, that the most abundantly expressed coronins in cells examined were dispensable for F-actin polymerization dynamics, and that tagging coronin proteins may result in overestimated colocalization with F-actin within cells and loss of function in vivo. Another proposed function for coronin proteins is the regulation of cAMP and Ca^2+^ signaling [13,18,22,24,26–29,69,82], consistent with their sequence and structural homology to the β subunit of the trimeric G protein complex [4–6]. Also, previous studies have shown that both *Dictyostelium* coronin A and mammalian coronin 1 regulate cAMP production and modulate cAMP-dependent signaling regardless of the presence or absence of an intact F-actin cytoskeleton [18,27,29,82,83].

Whereas, as is the case for coronin 1 [27,82], mammalian coronin 2 and 3 have been co-immunoprecipitated with the Gα_S_ subunit of the trimeric G protein complex [10], whether coronin 2 and 3 are also involved in the regulation of cAMP signaling is presently not known. To analyze a potential role for coronin 2 and 3 in cAMP signaling, while avoiding compensatory effects between the two coronin proteins, cells either expressing or lacking both coronin 2 and coronin 3 were transiently transfected with the Epac-S-H187 cAMP-reactive fluorescent reporter [84], stimulated with the adenylate cyclase activator forskolin [85] and imaged at the times indicated in Fig 7A and 7B. We found that in wild-type cells, forskolin addition resulted in rapid cAMP production as measured by ratiometric FRET analysis, that was significantly reduced in cells lacking coronin 2 and coronin 3 (Fig 7A and 7B, S1 Movie). To analyze whether reduced cAMP production was due to an aberration in the F-actin cytoskeleton, this measurement was also performed on cells pretreated with LatA to depolymerize F-actin, with similar results (Fig 7C and 7D, S1 Movie), suggesting that the decrease in cAMP production upon coronin 2 and 3 deletion is independent of an intact F-actin cytoskeleton. Of note, these results are in line with previously observed coronin 1-dependent cAMP production in cells upon complete depolymerization of F-actin [27]. Moreover, no significant differences were observed in cAMP production between LatA-treated and control cells within the same cell line, suggesting F-actin to be dispensable for forskolin-induced cAMP production. Furthermore, analysis of cAMP production using a FRET-based competition assay replicated the defect of coronin 2/3-deficient cells in cAMP production regardless of the presence of F-actin, while both coronin 2 and coronin 3-deficient cells (‘single knockouts’) showed an intermediate phenotype between wild-type and ‘double knockout’ cells (S9 Fig). Additionally, no apparent effect of actin depolymerization on cAMP production was observed within each cell line, confirming F-actin at large to be dispensable for this process. The data presented here provide evidence in favor of the ubiquitously expressed mammalian coronin 2 and 3 playing a role in cAMP production, and doing so independently of the polymerization state of the actin cytoskeleton, in agreement with prior findings on coronin 1 in mammals and coronin A in *Dictyostelium* [18,27,82].

**Fig 4:**
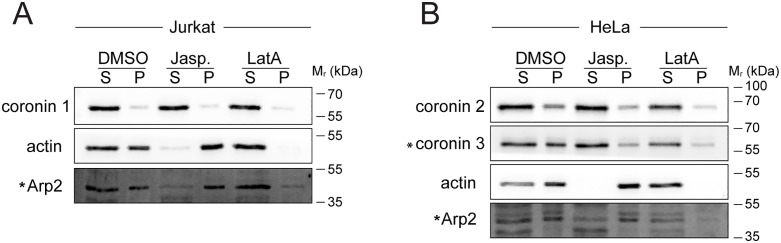
Analysis of coronin protein association with filamentous actin in cells. **(A, B)** Lysates of wild-type Jurkat (A) and HeLa (B) cells exposed to vehicle control (DMSO), actin-polymerizing (Jasp.) and actin-depolymerizing (LatA) treatment as described in the methods section were fractionated by ultracentrifugation at 150,000*g* for one hour, after which the supernatant and pellet fractions were collected, with the pellet resuspended in equal volume of buffer. Equal volumes of centrifuged lysates corresponding to material from the same cell numbers harvested were separated by SDS-PAGE and probed for the indicated antigens by Western blotting. Asterisks indicate blots from the same lysates probed for the indicated antigen on an equally loaded gel and membrane different from the membrane used for the actin blot. Raw images of blots can be found in S1 Raw Images.

**Fig 5:**
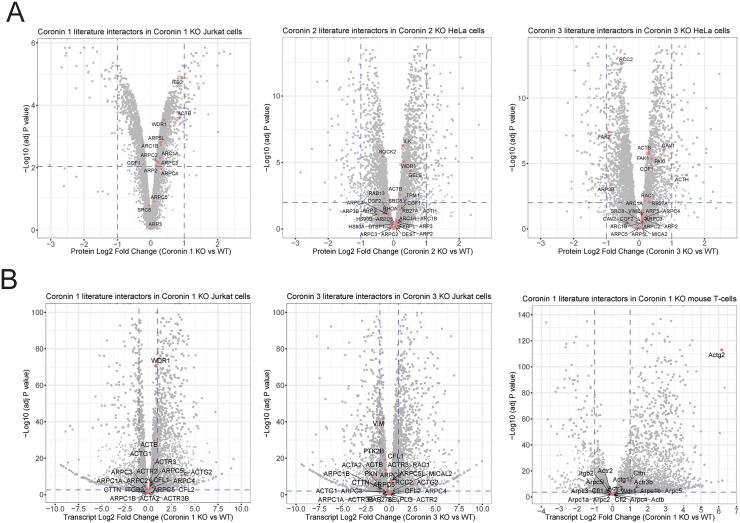
Expression of proteins involved in actin-related processes reported to be coronin-dependent are not affected by coronin deletion. **(A, B)** Volcano plots of proteomic (A) and transcriptomic (B) analysis of coronin-deficient vs. corresponding wild-type cells. Each data point represents a protein (A) or a gene (B). Vertical lines indicate the threshold of 2-fold change (Log2(fold change) <−1 and >1) and horizontal line represents the significance threshold of adjusted *P*-value below 0.01 (−Log10 (adjusted *P*-value) > 2), as calculated with (A) linear mixed model implemented in MSstats R package for proteomic analysis and (B) DESeq2 R package for transcriptomic analysis. Red data points indicate proteins (A) and corresponding genes (B) reported in literature to be regulated or to interact with each of coronin 1, 2 or 3 (for the lists of proteins see S3 and S4 Tables for references to papers describing their connection to coronins). For all experiments, the estimated power to detect a change above 2-fold with an adjusted *P*-value < 0.01 was at least 80%. Only proteins (A) and genes (B) expressed and detected in a given experiment are plotted. See also S2 Data.

**Fig 6:**
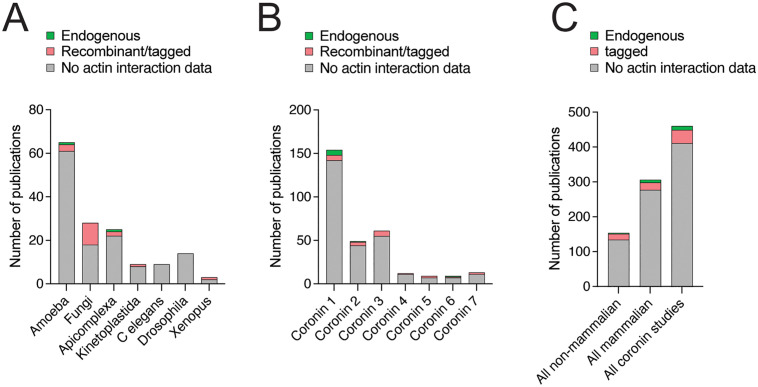
Bibliometric analysis on coronin proteins and actin interaction. **(A–C)** Bar graphs of the number of publications on non-mammalian (A), mammalian (B) or all (C) coronin/Crn1 proteins available on PubMed either not investigating actin interaction (gray) or demonstrating interaction with actin using endogenous (green) or recombinantly expressed and/or tagged coronin/Crn1 proteins (red). Publications on non-mammalian coronin proteins (A) were split according to the taxa. Publications on mammalian coronins (B) according to the family member investigated. See also S3 and S4 Data. Numeric values can be found in S1 Data.

**Fig 7:**
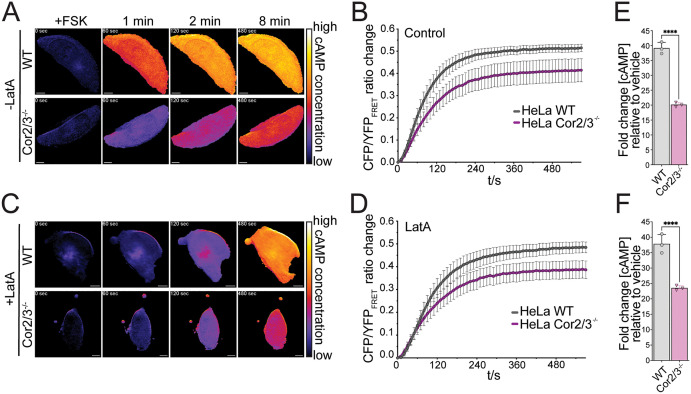
Production of cAMP in wild-type and coronin-deficient HeLa cells upon forskolin stimulation with and without an intact actin cytoskeleton. **(A–D)** Wild-type (WT) and coronin 2 and 3-deficient HeLa cells transfected with a plasmid expressing Epac-S-H187 cAMP-reactive FRET reporter were incubated for 30 min in 500 µM IBMX in the absence (A, B) or presence of 2 µM LatA (C, D). Cells were stimulated with 25 µM forskolin at *t* = 0 s and imaged in donor and FRET-acceptor channels every 10 s. Cells were analyzed with an ImageJ macro and representative cells are shown at the indicated timepoints (A, C), with warmer colors indicating increased cAMP concentration. Minimum display range values were normalized to the fluorescence at the time of stimulation (*t* = 0 s) and maximum values set to a common value of 1.4. Bars are 10 µm. Donor/acceptor ratios were averaged after setting the ratio value to 0 at the time point at which forskolin was added with at least 44 cells per condition (B, D). Error bars indicate 95% confidence intervals. Numeric values can be found in S1 Data. (E, F) Wild-type and coronin 2/3-deficient HeLa cells were incubated in 500 µM pan-phosphodiesterase inhibitor IBMX in the absence (E) or presence of 2 µM LatA (F) for 30 min, after which they were stimulated with either DMSO (vehicle) or 50 µM forskolin for 30 min. Concentration of cAMP was measured using a FRET-based immunocompetition kit, where donor and acceptor fluorescence were measured on a Tecan Spark plate reader. Calculated fold cAMP concentration changes upon forskolin stimulation relative to vehicle control are shown for each cell line. Bars represent standard deviation and statistical significance was examined by one-way ANOVA. ns - nonsignificant, ** *p* < 0.01, *** *p* < 0.001, **** *p* < 0.0001. Numeric values can be found in S1 Data.

### Discussion

A sizable body of published work supports the model of functional interactions between coronin proteins and F-actin, implicating coronins primarily and sometimes exclusively as actin-binding and -modulating proteins. The data presented here challenge this notion and support the existence of additional, signaling-proximal functions for coronin proteins, while demonstrating that overt reliance on tagged coronin proteins risks biasing the experimental outcome towards cytoskeletal functions.

We found that, when staining cells using specific antibodies, mammalian coronin 1, 2 and 3 colocalized with F-actin to a significantly lesser degree compared with tagged coronin proteins. Moreover, insertion of the FLAG tag into the mouse coronin 1-encoding locus phenocopied loss-of-function mutation in mice. Furthermore, the subcellular localization pattern of endogenous coronins did not depend on the presence of an intact F-actin cytoskeleton, while the absence of coronin 2 and/or 3 in turn did not affect F-actin distribution or polymerization dynamics within cells. This was further corroborated by biochemical analysis of endogenous and tagged coronin proteins co-pelleting with actin which revealed no notable alteration in supernatant-to-pellet ratio of all coronins examined regardless of the polymerization state of actin, suggesting that the bulk of coronin proteins does not associate with actin regardless of the tag. Finally, we show, as is the case for mammalian coronin 1 and *Dictyostelium* coronin A [18,27,29,82], that coronin 2 and 3 supported cAMP production independent of the presence of an intact F-actin cytoskeleton. These data, that are in line with previous work on coronin proteins from amoeba to mammals [18,19,22–26,29], may prompt a re-evaluation of the function(s) for coronin proteins independent of direct F-actin modulation.

The link between coronin protein function and F-actin modulation may be due to coronin having been identified as a protein copurifying with actin/myosin complexes in cytoplasmic extracts of the slime mould *Dictyostelium discoideum* [4]. Moreover, the majority of studies analyzing F-actin interaction and modulation were performed using the yeast coronin-like protein, Crn1, that, in contrast to non-fungal proteins, possesses several domains that share homology with proteins known to interact with Arp2/3 complexes [1,2,79]. Furthermore, yeast coronin-like protein was reported to interact with mammalian actin more strongly than with yeast actin [81].

Complexes of tagged versions of yeast Crn1 as well as mammalian coronin proteins with actin have been analyzed at the structural level revealing different interaction modes depending on the F-actin nucleotide state, where coronin facilitates phosphate release from actin filaments in vitro [81,86], a function that may be related to the role for coronin proteins in the modulation of GαS activity [27,82], or interaction with small GTPases [87–89]. While it is unclear whether such function of coronin proteins is also relevant in the cellular context and whether untagged coronin proteins could perform differently, it plausibly links the proposed roles of coronin proteins in F-actin dynamics and signaling.

Based on the results described here, we argue that relying solely on the analysis of tagged coronin proteins bears significant risks of investigating non-physiological phenomena. This notion is in agreement with the fact that protein tags are known to potentially alter the protein’s localization and/or function [38,39,60,61]. Moreover, GFP is known to interact with actin-binding sites of myosin [57,58], possibly contributing to the F-actin relocalization of GFP-tagged coronin proteins as compared to their non-tagged, endogenous counterparts. Alternatively, tagging coronin proteins with GFP may expose a cryptic actin binding site on the tagged coronin protein [90–92], although this is unlikely given that GFP-tagged coronin proteins co-pelleted with filamentous actin to a similar degree regardless of the polymerization state of actin. An additional possibility could be that tagging coronin proteins alters post-translational modifications [68,82,93,94], their oligomerization state [5,69,95], or membrane association [5,95] contributing to their relocalization. Therefore, even though tags may provide useful tools for analyzing protein interactions and localization, verifying their functionality and localization relative to the endogenous proteins is important. In our work, we have encountered unexpected losses of coronin protein function in cell population maintenance upon tagging, examples of which include FLAG-tagging of coronin 1 in mice shown in this work, and CTAP-tagging of coronin A in *Dictyostelium* for the purpose of interactome analysis [96]. Contrarywise, FLAG-coronin A proved to be innocuous for cell population establishment in *Dictyostelium*, readily rescuing the phenotypes of coronin A-deficient cells [13,97], which does not appear to be the case for coronin 1 in mice as shown here. This suggests that functionality of tagged coronin proteins may need to be examined on a case-by-case basis, as it could vary in a cell type and/or context-dependent manner.

Additionally, detecting coronins with specific antibodies poses a unique challenge in immunofluorescence analyses, likely due to a high degree of evolutionary conservation, their homology in sequence and structure, and expression of multiple coronin proteins in a given cell type. In our hands, several (for coronin 2) or all (for coronin 3) of the antibodies screened upon the commonly used 4% PFA fixation of cells, including antibodies used in the literature to show subcellular localization of coronin 2 and/or 3, were found to be nonspecific since they failed to distinguish between wild-type and the respective knock-out cells. However, avoiding the use of antibodies instead employing expression of fluorescently tagged coronin proteins may result in artificially high colocalization with the actin cytoskeleton.

Together, the data reported here align with findings studying endogenously expressed and non-tagged coronin proteins across species that show subcellular localization of endogenous coronin proteins to be independent of F-actin and actin-related-pathways to be functional regardless of the presence or absence of coronin proteins [17–20,24,26,27,48,98,99]. Furthermore, the here-demonstrated role for coronin 2 and 3 in the regulation of cAMP production is not only consistent with previous reports of coronin-dependent cAMP production [18,27,29,82] but also the sequence and structural homology of coronin proteins to the β subunit of trimeric G proteins [4–6]. This is of particular interest since the interaction of Gβ proteins with the Gαs subunit is known to be involved in the activation of adenylate cyclase activity leading to cAMP production [100–102]. While we cannot exclude a role for coronin proteins in F-actin depolymerization, both cAMP and Ca^2+^ signaling has been implicated in regulation of the cytoskeleton [103], possibly providing a link between coronin function and F-actin rearrangement in cells independently of a direct effect on the cytoskeleton.

Given the importance of coronin proteins in a wide range of physiological processes including immunity, development, tissue architecture and cell population size regulation, the work reported here provides impetus to define the roles of coronin proteins in signaling and unravel the underlying molecular mechanisms.

## Materials and methods

### Reagents and Resources

All reagents and resources used in this study are described in Table 1.

**Table 1:** Reagents and Resources used in this study.

Reagent/Resource	Reference or Source	Identifier or Catalog Number
**Experimental Models**		
Human: Jurkat T cells cl. E6-1	ATCC	RRID:CVCL_0367
Human: HeLa	ATCC	RRID:CVCL_0030
Jurkat Cor1KO	Ndinyanka Fabrice and colleagues (2022), PMID: 36346836	
HeLa Cor2 KO	This paper	
HeLa Cor3 KO	This paper	
HeLa Cor2/3 KO	This paper	
JLA20 hybridoma	Developmental Studies Hybridoma Bank	RRID: AB_528068
Mouse, C57BL/6JRj strain	Javier Labs	
Mouse, coronin 1-deficient	Kaminski and colleagues (2011), PMID: 21700422	
Mouse, FLAG knock in	This paper	
Mouse embryonal fibroblasts	Isolated in house	
Mouse: J774.A1	ATCC	RRID: CVCL_0358
Human: Mel JuSo	Pieters and colleagues (1991), PMID: 1955469	RRID:CVCL_1403
Mel JuSo cor1^+^	Ferrari and colleagues (1999), PMID: 10338208	
Mel JuSo cor1-EGFP^+^	This paper	
**Recombinant DNA**		
mEmerald-Coronin1B-N-10	Addgene, by Michael Davidson	ID #54050
mEmerald-Coronin1B-C-10	Addgene, by Michael Davidson	ID #54049
mEmerald-C1	Addgene, by Michael Davidson	ID #53975
mEmerald-Coronin1-N-10	This paper	
mEmerald-Coronin1-C-10	This paper	
mEmerald-Coronin3-N-10	This paper	
pEGFP-N1::hCor1 #3	Available in the laboratory	
pPR-CMV-hCor3-FLAG	Available in the laboratory	
Epac-S-H187	Addgene, by Klarenbeek and colleagues 2015	ID# 170,348
**Antibodies**		
CORO1A monoclonal antibody (M01), clone 4G10	Abnova, CAT: H00011151-M01	RRID:AB_606085
Coronin 1 rabbit polyclonal antibody	In house, Ferrari and colleagues (1999), PMID: 10338208	
Coronin 2 monoclonal antibody, clone 5-7.1	Takeshi Urano	
Coronin 1B monoclonal antibody, clone G-8	Santa Cruz Biotechnology, CAT: sc-271445	RRID:AB_10659094
Coronin 1B Polyclonal Antibody	Thermo Fisher, CAT: PA5-64443	RRID:AB_2640078
Anti-CORO1B polyclonal antibody	Sigma	RRID:AB_2686268
Anti-CORO1B polyclonal antibody	Sigma	RRID:AB_2676850
Coronin 2 rabbit polyclonal antibody	You and colleagues.	
Coronin 3 mouse monoclonal antibody cl. K6-444	Eichinger and colleagues.	
Coronin 3 rabbit polyclonal antibody	Invitrogen, CAT: PA5-30479	RRID:AB_2547953
Coro1C rabbit polyclonal antibody	Proteintech, CAT: 14749-1-AP	RRID:AB_2230110
Anti-Coronin-1C goat polyclonal antibody	Abcam, CAT: ab15719	RRID:AB_302060
Recombinant Anti-Coronin-1C antibody [EPR25365-24]	Abcam, CAT: ab283693	
CORO1C, Mouse anti-Human, Clone: 1F7	Abnova, CAT: H00023603-M02	RRID:AB_1137146
Coronin 1C Antibody (D-9)	Santa Cruz Biotechnology, CAT: sc-376919	
Coronin 1C Antibody (G-R2)	Santa Cruz Biotechnology CAT: sc-130448	RRID:AB_2087033
Alexa Fluor 700 anti-mouse CD3	Biolegend, CAT: 100216	RRID:AB_493697
Brilliant Violet 510 anti-mouse CD4	Biolegend, CAT: 100559	RRID:AB_2562608
Spark Blue 550 anti-mouse CD8a	Biolegend, CAT: 100780	RRID:AB_2819773
Anti-FLAG antibody	Cell Signaling, CAT: 2368S	RRID:AB_2217020
Goat anti-mouse AF488	Thermo Fisher, CAT: A-11029	RRID:AB_2534088
Donkey anti-rabbit AF488	Thermo Fisher, CAT: A21206	RRID:AB_2535792
Goat anti-chicken AF568	Thermo fisher, CAT: A11041	RRID:AB_2534098
Donkey anti-mouse AF647	Life Technologies, CAT: A31571	RRID:AB_162542
Donkey anti-rabbit AF568	Thermo fisher, CAT: A10042	RRID:AB_2534017
Chicken anti-GFP polyclonal antibody	Millipore CAT: 06-896	RRID:AB_310288
Goat anti-mouse IgG-conjugated HRP	Southern Biotech	RRID:AB_2794340
Anti-mouse IgG, HRP-linked Antibody	Cell Signaling Technology, CAT: 7076S	RRID: AB_330924
Anti-rabbit IgG, HRP-linked Antibody #7,074	Cell Signaling Technology, CAT: 7074S	RRID: AB_2099233
Mouse anti-actin (cl. JLA20)	In-house hybridoma from Developmental Studies Hybridoma Bank	RRID:AB_528068
Donkey anti-rabbit AF647	Thermo Fisher	RRID:AB_2536183
Anti-S6 ribosomal protein rabbit polyclonal antibody	Cell Signalling Technology, CAT: 4857S	
Mouse anti-β-actin (cl. C2)	Santa Cruz Biotechnology CAT: sc-8432	RRID: AB_626630
Rabbit anti-GFP (cl.D5.1)	Cell Signalling Technology, CAT: 2956S	RRID: AB_3713179
Rabbit anti-Arp2	Santa Cruz Biotechnology CAT: sc-15389	RRID: AB_2221848
**Oligonucleotides and other sequence-based reagents**		
hCor2-pSpCas9-GFP-sgRNA2 5′-CACCGCGCATCATCGACCCCCGTCG-3′	Microsynth	
hCor3-pSpCas9-GFP-sgRNA-2 5′-CACCGATAATCATAGAGGCAAGTGG-3′	Microsynth	
Fwd. primer for amplification of coronin 1 from pEGFP-N1::hCor1 #3 5′- ATATAGATCTGCCACCATGAGCCGGC-3′	Microsynth	
Rev. primer for amplification of coronin 1 from pEGFP-N1::hCor1 #3 5′- CGGTGGATCCCTACTTGG-3′	Microsynth	
Fwd. primer used for amplification of coronin 3 from pPR-CMV-hCor3-FLAG 5′- ATATGCTAGCGCCACCATGAGGCGAGTGGT-3′	Microsynth	
Rev. primer used for amplification of coronin 3 from pPR-CMV-hCor3-FLAG 5′- ATATGGATCCCCGCTACCGCCGGCTGCTATCTTTGCC-3′	Microsynth	
Alt-R CRISPR-Cas9 crRNA for FLAG -KI mice generation 5′ -CUGTAUCCUUCAGGAUGAGC- 3′	Integrated DNA Technologies	
Alt-R CRISPR-Cas9 tracrRNA	Integrated DNA Technologies CAT: 1072532	
Ultramer FLAG-Coro1 for FLAG-KI mice generation 5′ - CCTCAAGGCCAGGCCAAGGGTTGCTCTGGGGATGACAAAGTGCGGCTGGGATTGACCCAAAGAAAAGTTTACAATCTGTATCCTTCAGGATGGACTACAAGGACGACGATGACAAGAGCCGGCAGGTGGTTCGCTCCAGCAAATTCCGCCACGTGTTTGGACAGCCAGCCAAGGCTGACCAGTGCTATGAGGATGTGCGC -3′	Integrated DNA Technologies	
Genotyping Primer, mCor1_forward 5′ -ATGGTGCAGGATTGAGGGTG- 3′	Microsynth	
Genotyping Primer, FLAG_ forward 5′ -ACAAGGACGACGATGACAAG- 3′	Microsynth	
Genotyping Primer, mCor1_reverse 5′ -CTCCCCTTCCTGTTTCACCC- 3′	Microsynth	
Human coronin 1 forward 5′-CTGTGCTGTCAACCCTAAGTT-3′	Microsynth	
Human coronin 1 reverse 5′-GTGGGCGCATTCTTGTCCA-3′	Microsynth	
Human coronin 2 forward 5′-CAG GCT TCA GCC GAA TGA G-3′	Microsynth	
Human coronin 2 reverse 5′-CGC AGA CGT AGA CCA CAC T-3′	Microsynth	
Human coronin 3 forward 5′-GAC CTG GGA TAG TTC CTT TTG TG-3′	Microsynth	
Human coronin 3 reverse 5′-AAT TCG ACC AGT CTT GTG CAG-3′	Microsynth	
Human coronin 4 forward 5′-CCC CAC TAC CCA AAA GTC TGC-3′	Microsynth	
Human coronin 4 reverse 5′-CAG GAG GCG ATC TCA AAA TCA TC-3′	Microsynth	
Human coronin 5 forward 5′-CAG GAT TGA ACC CAA CTA CCC-3′	Microsynth	
Human coronin 5 reverse 5′-GAG CAC GAG GCA ATG ATG TT-3′	Microsynth	
Human coronin 6 forward 5′-AGA CAG GGC GAG TGG ATA AGA-3′	Microsynth	
Human coronin 6 reverse 5′-CAT TGT GTG GAC ACC AGT CAA-3′	Microsynth	
Human coronin 7 forward 5′-GCT GCC ATT CAG ACC TAG TCA-3′	Microsynth	
Human coronin 7 reverse 5′-AGT CGC CAG AGT TTT ACC GTC-3′	Microsynth	
GAPDH forward 5′-GGAGCGAGATCCCTCCAAAAT-3′	Microsynth	
GAPDH reverse 5′-GGCTGTTGTCATACTTCTCATGG-3′	Microsynth	
**Chemicals, Enzymes and other reagents**		
DAPI	Sigma-Aldrich, CAT: D9542	LOT: 0000111504
Phalloidin-647I	Abnova, CAT: U0298	LOT: 3240741
WGA-CF568	Biotium, CAT: 29077-1	LOT: 23W0329-1216072
Latrunculin A	Focus Biomolecules, CAT: 10-2254	LOT: FBA3069
Cytochalasin D	Focus Biomolecules, CAT: 10-2071	LOT: X102185
Jasplakinolide	Tocris, CAT: 2792	LOT: 11A
RPMI-1640	Sigma-Aldrich, CAT: R8758	
DMEM	Sigma-Aldrich, CAT: D6429	
Fetal bovine serum	Gibco, CAT: 10207-106	LOT: 2177362
Fetal bovine serum	Sigma, CAT: F0804	LOT: BCCC4867
Penicillin-Streptomycin	Sigma-Aldrich, CAT: P4333	
0.05% Trypsin-EDTA	Gibco, CAT: 25300-054	
PBS	Sigma-Aldrich, CAT: D8537	
HBSS	Gibco, CAT: 14025092	
TurboFect transfection reagent	Thermo Scientific, CAT: R0531	
Cell-Tak	Corning, CAT:354240	
Paraformaldehyde	Sigma-Aldrich, CAT:158127	
Methanol	Sigma-Aldrich, CAT: 32213	
Tween 20	Sigma, CAT: 93773	
NP-40 alternative	Millipore, CAT:492016-100ML	
ATP	Sigma, CAT:A7699	
BSA	Sigma-Aldrich, CAT: A9647	
Vectashield Vibrance Antifade mounting medium	Vector Labs, CAT: H-1700	
Immersol 518F	Zeiss, CAT: ISO 8036	
BamHI HF	New England Biolabs, CAT: R3136S	
BamHI	New England Biolabs, CAT: R0136S	
NheI HF	New England Biolabs, CAT: R3131S	
BglII	New England Biolabs, CAT: R0144S	
KpnI-HF	New England Biolabs, CAT: R3142S	
AflI	New England Biolabs, CAT: R0520S	
AgeI-HF	New England Biolabs, CAT: R3552S)	
Q5 High Fidelity DNA Polymerase	New England Biolabs, CAT: M0491S	
Shrimp Alkaline Phosphatase (SAP)	Promega, CAT: M8201	
T4 DNA ligase	Promega, CAT: M180B	
T4 Polynucleotide kinase	New England Biolabs, CAT: M0201L	
β-mercaptoethanol	Sigma, CAT: M7522	
glycerol	Sigma, CAT:49770-1L	
Kanamycin sulfate	BioChemica, CAT: A1493,0025	
Ampicillin Sodium Salt	BioChemica, CAT: A0839,0025	
Fixable Live/Dead marker	Thermo Fisher, CAT: L10119	
Protease/Phosphatase inhibitor	Thermo Fischer, CAT: 1861281	
Paraformaldehyde, pH 6.9	Sigma, CAT:10049960700	
Saponin	Sigma, CAT: S7900	
Triton X-100	Sigma, CAT: 93443	
Keyhole-limpet hemocyanin	Thermo Scientific, CAT: 77666	
Freund’s Complete Adjuvant	Sigma-Aldrich, CAT: F5881	
Freund’s Incomplete Adjuvant	Sigma-Aldrich, CAT: F5506	
Polyethylene glycol	Sigma-Aldrich, CAT: 3,350, P4338	
HAT medium	MP Biomedicals, CAT: 16-808-49	
RIPA buffer	Produced in-house	
SDS sample buffer	Produced in-house	
FACS buffer	Produced in-house	
F-actin stabilization buffer	Produced in-house	
GTAEELYRLDSLHPD-C, peptide mixture for coronin 2	This paper	
SLREAYVPSKQRD-C, peptide mixture for coronin 2	This paper	
Alt-R S.p. HiFi Cas9 Nuclease V3, 100 μg, 10 μl	IDT, CAT: 1081060	
EDTA solution	Produced in-house	
Tris-glycine buffer	Produced in-house	
MgCl_2_	Sigma, CAT:M8266-1KG	
PIPES	Sigma, CAT: P1851-100G	
DMSO	Sigma, CAT: D2438	
IBMX	Tocris, CAT: 2845	
Forskolin	AG scientific, CAT: F-1026	
PMA	Tocris, CAT: 1201	LOT: 25A/289273
Chelerythrine	Tocris, CAT: 1330	LOT: 8D/285544
Sodium othovanadate	Merck, CAT: 567540-5GM	LOT: 4257073
Hydrogen peroxide	Merck, CAT: K40677109	
**Software**		
*Huygens Remote Manager*		RRID:SCR_014237
*Huygens Professional*		RRID:SCR_014237
*Huygens Essential*		RRID:SCR_014237
*Fiji* ImageJ		RRID: SCR_002285
*BIOP JACoP* plugin for *Fiji*	PTBIOP, EPFL Lausanne	
*Zeiss Zen Blue 3.2*		RRID:SCR_013672
*Graphpad Prism 10*		RRID:SCR_002798
FlowJo		RRID:SCR_008520
OMERO	OME	RRID:SCR_002629
*Adobe Illustrator*		RRID:SCR_010279
**Other**		
Competent cells E. coli 10-β	Generated in-house	
Neon Transfection System	Thermo Fisher, CAT: 10431915	
NepaGene electroporator	NepaGene	
Plasmid Plus Midi Kit	Qiagen, CAT: 12943	
EndoFree Plasmid Maxi Kit	Qiagen, CAT: 12362	
QIAquick Gel Extraction Kit	Qiagen, CAT: 28704	
PCR Cloning kit	New England Biolabs, CAT:E1202	
Western Bright Quantum Kit	Advansta, CAT:K-12042-D10)	
Attune CytPix Flow Cytometer	Invitrogen, CAT: A48671	
eBlot L1 Protein Transfer System	GenScript	
SurePAGE, Bis-Tris, 10x8, 4%–20%, 15 wells	Genscript, CAT: M00657	
Ultracentrifuge	Eppendorf, model MicroUZ CS150FNX with fixed angle rotor model S150AT	
IsoStrip Mouse Monoclonal Antibody Isotyping Kit	Roche, CAT: 11493027001	
BCA assay	Thermo Fisher, CAT: 23223; CAT: 1859078	
HTRF cAMP Gs Dynamic kit	Perkin Elmer, CAT: 62AM4PEB	
13 mm microscopy glass cover slips	VWR, CAT: 631-0150	
Microscopy slides	Huber Lab, CAT: 300741	
Nitrocellulose membrane	Thermo Scientific, CAT:88018	
Costar 24-well Clear TC-treated Multiple Well Plates	Corning, CAT: 3524	
Costar 6-well Clear TC-treated Multiple Well Plates	Corning, CAT: 3506	
Corning 96-well Flat Clear Bottom Black Polystyrene TC-treated Microplates	Corning, CAT: 3603	
Lab-Tek II Chambered #1.5 German Coverglass System	Thermo Fisher, CAT: 155409	
Corning 25 cm^2^ Rectangular Canted Neck Cell Culture Flask with Vent Cap	Corning, CAT: 430639	
ProxiPlate-384 Plus (White, Shallow 384-Well, Pinch bar design)	Perkin Elmer, CAT: 6008280	
Syringe 27G	Braun, CAT:4657705B	
Thick-wall ultracentrifugation tubes	Beckman-Coulter, CAT:343778	
150 µm polyamide mesh	Sefar Nitex, CAT: 03-150-38	

### Animals

Mice (wild type C57BL/6J, coronin 1-deficient [104], kindly provided by Dr. Josef Penninger, Vienna, Austria, and FLAG-Cor1 Knock-In mice (see below)), were bred according to cantonal ethic and husbandry standards at the animal facility of the Biozentrum, University of Basel. Both male and female littermates (8–17 weeks) were used for the studies.

### Ethics statement

All mouse experiments were performed according to the governing cantonal veterinary rules of the Canton of Basel, Switzerland (license number 1893) or the animal care and use committee of Shimane University, Japan (ID: IZ2-58).

### Generation of FLAG-Cor1 knock-in mice

The Coro1-Nt-FLAG allele was obtained by Cas9/CRISPR embryo electroporation. The FLAG sequence was inserted at the N-terminus to the Coro1 coding region. The Cas9/CRISPR target sequence ccttcaggatgagccggcagg(tgg) (PAM sequence in brackets) was selected with the help of CRISPOR software (http://crispor.tefor.net/). A ssDNA oligonucleotide *cctcaaggccaggccaagggttgctctggggatgacaaagtgcggctgggattgacccaaagaaaagtttacaatctgtatccttcaggatg***gactacaaggacgacgatgacaag***agccggcaggtggttcgctccagcaaattccgccacgtgtttggacagccagccaaggctgaccagtgctatgaggatgtgcgc* was designed to insert the FLAG tag (in bold) into the Cas9-generated double strand break (DSB) by homologous recombination downstream of the ATG codon of the *coro1a* gene (underlined) with the help of 5′ and 3′ homology arms (italic) [105]. Electroporation with a mixture of ssDNA oligonucleotide targeting template, 16uM cr:trcrRNA hybrid targeting Coro1 and 16uM Cas9 protein (all reagents from IDT) was carried out using 1 mm gap electroporation cuvette and the NepaGene electroporator as previously described [106]. Surviving embryos were washed with M16 medium (Sigma) and transferred immediately into the oviducts of 8–16-weeks-old pseudopregnant Crl:CD1(ICR) females that had been mated with sterile genetically vasectomized males [107] the day before embryo transfer (0.5 dpc). Pregnant females were allowed to deliver and raise their pups until weaning age. Coro1-Nt-FLAG positive F0 founder mice were outcrossed with C57BL/6JRj (Javier Labs) wild type mice to produce FLAG knock-in homozygous mice, where the expression of FLAG tag was confirmed in spleen, lymph nodes, and blood using flow cytometry.

### Cell lines

Jurkat T cells (clone E6.1), HeLa cells and J774.A1 macrophages were purchased from ATCC. All cell lines used in the study were authenticated by Microsynth (Switzerland). For regular Jurkat T cells’ culturing RPMI-1640 (Sigma-Aldrich, CAT: R8758) media supplemented with 10% FBS (Gibco, CAT: 10207-106), 100 U/mL penicillin, and 100 µg/mL streptomycin (Sigma-Aldrich, CAT: P4333) was used. For regular HeLa cells and mouse embryonic fibroblasts (MEF) culturing DMEM media (Sigma-Aldrich, CAT: D6429) supplemented with 10% FBS (Sigma, CAT: F0804) and 100 U/mL penicillin, and 100 µg/mL streptomycin (Sigma-Aldrich, CAT: P4333) was used. For regular J774.A1 culturing DMEM media (Sigma-Aldrich, CAT: D6429) supplemented with 10% heat-inactivated FBS (Gibco, CAT: 10207-106), 100 U/mL penicillin, and 100 µg/mL streptomycin (Sigma-Aldrich, CAT: P4333) was used. All cell lines were cultured at 37 °C in a humidified atmosphere containing 5% v/v CO_2_.

Jurkat T cells were harvested by centrifugation and subcultured every fifth day. HeLa and MEFs (up to three passages only) were harvested and subcultured upon reaching 80%–90% confluency using trypsinization (trypsin-EDTA by Gibco, CAT: 25300-054). J774.A1 macrophages were passed upon reaching 70%–80% confluency (every third day) and were collected for that purpose by centrifugation after 20 min incubation in calcium- and magnesium-free PBS followed by vigorous pipetting. Mel JuSo cells were originally from Dr. Johnson [108] and cultured as described earlier [109,110].

### Generation of Hela cells deficient in coronin 2 and/or coronin 3

HeLa cells (ATCC) were cultured in a 6-well plate until ~90% confluency, harvested and transfected using the Neon transfection system (Thermo Fisher, CAT: 10431915). The following plasmids were used: hCor2-pSpCas9-GFP-sgRNA2 for generation of Cor2-KO cells. The sgRNA primers were first phosphorylated and annealed using the T4 polynucleotide kinase (NEB, CAT: M0201L). The annealed primers were and the pSpCas9(BB)-2A-Puro (PX459) V2.0 vector (Addgene, plasmid CAT: 62988) were digested with BbsI and ligated in a Golden gate cloning step. Competent 10-β *E. coli* cells were transformed using the heat-shock procedure, plated on LB-agar plates with 100 µg/ml Ampicillin, single colonies were picked, expanded and the cloning was confirmed by sequencing.

For generation of Cor3-KO cells sgRNA hCor3-pSpCas9-GFP-sgRNA-2 was used. The transfected cells were seeded in antibiotic-free medium, expanded and single clones were sorted based on the GFP expression (both plasmids contained EGFP in the same operon as the Cas9 enzyme), using the FACS Aria III Cell Sorter (BD Biosciences) with a 488 nm laser and a 514/30 nm bandpass filter. The clones were then expanded and validated in Western blotting and Flow cytometry to be later used for immunofluorescence staining.

For the generation of Cor2/3 double knockout (DKO) cells, the procedure was performed analogously, with both sgRNAs transfected into the cells using the XFect Transfection Reagent (Takara, #631318).

### Generation of the coronin 1 and 3 deficient Jurkat T cells

Jurkat T cell coronin 1 KO ware generated as described [13]. Jurkat T cell coronin 3 KO were generated by CRISPR-Cas9 genome editing. The plasmid pSpCas9(BB)-2A-GFP (green fluorescent protein) (PX458) was a gift from F. Zhang (Addgene plasmid #48138). A 20-nucleotide sequence targeting the third exon of human coronin 1 followed by a protospacer adjacent motif from *Streptococcus pyogenes* [CAGGGGCTCACCTTGCCCAG(GGG)] was selected using publicly available tools for the least number of potential off-target sites. The sequence was cloned into PX458 as previously described [111]. Jurkat T cells (1 × 106) were transfected with 7.5 μg of pX458-sgRNA plasmid in a six-well tissue culture plate using the Xfect Transfection Reagent (Takara Bio, catalogue no. 631318) according to the manufacturer’s protocol. GFP-expressing cells were sorted 48 hours after electroporation, enriched, and then sorted again after a week and assessed for editing (loss of coronin 3 expression). After confirming the occurrence of edited cells, we sorted the cells into 20, 10, 5, and 1 cell per well into 96-well tissue culture plates, expanded the clones, and screened for coronin 3 knockout clones by flow cytometric and microscopic analysis of intracellularly stained cells. More than 20 coronin 3-deficient clones were isolated and showed a similar phenotype in culture. Absence of coronin 3 expression was further confirmed by immunoblotting and genotyping using the Inference of Crispr Edited-based webtool (https://ice.synthego.com/).

### Anti-coronin 2 antibody production

Mouse mAbs against coronin 2 were produced by immunizing mice with the mixture of peptides GTAEELYRLDSLHPD-C and SLREAYVPSKQRD-C conjugated with keyhole-limpet hemocyanin to the C-terminus of the peptides using the added cysteine (C) during peptide preparation. The kit used was the Imject Maleimide-Activated mcKLH spin Kit (77666, Thermo Scientific). Each KLH crosslinked peptide was mixed 1:1 for immunization. Emulsions were prepared by mixing 150 mL of KLH-conjugated peptide/PBS and 150 mL of Freund’s Complete Adjuvant (CAT: F5881, Sigma-Aldrich) and inoculated subcutaneously (*N* = 3). Two weeks later, emulsions of 150 mL of KLH-conjugated peptide/PBS and 150 mL of Freund’s Incomplete Adjuvant (CAT: F5506, Sigma-Aldrich) were inoculated subcutaneously. Incomplete Adjuvant was used for the third, fourth, and subsequent inoculations, which were given every week. Serum titers were monitored by immunoblotting, using the cell lysate transfected with coronin 2-encoding plasmids. Spleen cells from mice with elevated antibody titers were prepared and fused with Sp2/O-Ag14 cells using polyethylene glycol (CAT: 3,350, CAT: P4338, Sigma-Aldrich), and fused cells were selected with HAT medium (CAT: 16-808-49, MP Biomedicals, Santa Ana, CA, USA) after two days. Clonal populations of fusion cells were screened by ELISA for antibody production against the peptides conjugated with BSA. Productive cells were cloned to monoclonal lines by serial dilution screening. The isotypes of mAbs were confirmed using an IsoStrip Mouse Monoclonal Antibody Isotyping Kit (Roche, CAT: 11493027001). Highly concentrated mAbs were isolated from murine ascites after intraperitoneal injection of the hybridoma cells. All animal experiments were performed in compliance with the standards established by the International Guiding Principles for Biomedical Research Involving Animals and were approved by the animal care and use committee of Shimane University (ID: IZ2-58).

### Western blotting

Cells were washed twice in PBS and lysed in RIPA buffer (0.1% SDS, 50 mM Tris, 1% NP-40, 0.5% sodium deoxycholate, 1 mM MgCl_2_, 100 mM NaCl, 1 mM EDTA), supplemented with Protease/Phosphatase inhibitor mix (Thermo Fisher, CAT: 1861281). The lysates were centrifuged at 12,500*g*, 15 min, 4 °C and the protein concentration in the lysates was determined with BCA assay (Thermo Fisher, CAT: 23223; CAT: 1859078). SDS sample buffer (5% SDS, 0.6 mg/ml bromophenol blue, 30% glycerol, 0.3 M Tris-HCl, 16% β-mercaptoethanol, pH 6.8) was added to the lysates, samples were incubated at 95 °C for 5 min and 10 µg total protein was loaded per lane into a 10% acrylamide/bisacrylamide gel. For actin pelleting experiments, gels were loaded corresponding to the material from same cell numbers instead, and either the 12.5% acrylamide/bisacrylamide gels or 4%–20% precast SurePAGE gels (Genscript, CAT: M00657) were used. Proteins were separated at constant voltage of 120–200 V and transferred onto a nitrocellulose membrane (Thermo Scientific, CAT: 88,018) using the eBlot L1 Protein Transfer System (GenScript) or tank blot transfer for 1 h at 100V in transfer buffer (Tris-glycine, v/v 20% MeOH). Efficient protein transfer was assessed by Ponceau Red stain and subsequently washed in PBS/0.2% Tween20. Membranes were blocked for 1 hour with blocking solution (5% milk or 3% BSA in PBS-Tween20) and incubated either overnight at 4 ˚C or for 30 min at room temperature with primary antibodies, dissolved in blocking solution. Membranes were washed three times with PBS/0.2% Tween20 (PBS-T) and incubated with secondary antibodies, conjugated with HRP that was dissolved in blocking solution. Membranes were then washed three times with PBS-T and once with PBS, the HRP-conjugated secondary antibodies were visualized with Advansta Western Bright Quantum kit (CAT: K-12042-D10). The following antibodies were used for Western blotting: anti-coronin 2 (cl.5-7.1, generated in mouse, 1:1,000), anti-coronin 3 (cl. K6-444, generated in mouse, obtained from L. Eichinger, 1:75), anti-actin (cl. JLA-20 generated in mouse, 1:20), anti-β-actin (cl. C2, generated in mouse, Santa Cruz Biotechnology CAT: sc-8432, 1:500), anti-GFP (cl.D5.1 generated in rabbit, Cell Signalling Technology, CAT: 2956S, 1:1,000), anti-Arp2 (generated in rabbit, Santa Cruz Biotechnology CAT: sc-15389, 1:1,000), anti-mouse, HRP-conjugated antibody (generated in goat, Southern Biotech CAT: 1034-05, 1:10,000), anti-mouse, HRP-conjugated antibody (generated in horse, Cell Signaling Technology CAT: 7076S, 1:10000), anti-rabbit (generated in goat, Cell Signaling Technology, CAT: 7074S, 1:10,000).

### Filamentous actin pelleting experiments

The experiments were performed as described previously [19,22,73]. In brief, Jurkat cells were collected from full growth medium (RPMI-1640 (Sigma-Aldrich, CAT: R8758), 10% FBS (Gibco, CAT: 10207-106), 100 U/mL penicillin, and 100 µg/mL streptomycin (Sigma-Aldrich, CAT: P4333)) by centrifugation, washed once in PBS and counted using the Neubauer chamber. The cells were then centrifuged again and resuspended in RPMI-1640 medium without serum or antibiotics, with the addition of 2 µM jasplakinolide (Tocris, CAT: 2792), 10 µM latrunculin A (Focus Biomolecules, CAT:10-2254-100uG), or equivalent volume of DMSO (Sigma, CAT: D2438) as a vehicle control, since the jasplakinolide and latrunculin stocks were prepared in DMSO. The cells were treated for 45 min in the incubator at 37 °C and 5% CO_2_. Afterwards, the cells were collected by centrifugation, the medium aspirated, and lysed in F-actin stabilization buffer (50 mM PIPES (Sigma, CAT:P1851-100G), 50 mM NaCl (Sigma, CAT:S7653-1KG), 5 mM MgCl_2_ (Sigma, CAT:M8266-1KG), 5 mM EGTA (Roth, CAT:3054.3), 5% v/v glycerol (Sigma, CAT:49770-1L), 0.1% v/v Triton X-100 (Sigma, CAT:93443–100ML), 0.1% v/v NP-40 alternative (Millipore, CAT:492016-100ML), 0.1% v/v Tween 20 (Sigma, CAT:93773-1KG), 0.1% v/v β-mercaptoethanol (Sigma, CAT:M7522-250ML) in ddH_2_O, buffered to pH 6.9 at ambient temperature. Stored at 4°C and an aliquot was taken before each use, to which 1 mM ATP (Sigma, CAT:A7699) and 1% v/v protease and phosphatase inhibitor cocktail (Thermo, CAT: 1861281) were added) in the volume corresponding to the cell concentration of 20 million per ml. The suspension was passed through a 27G syringe (Braun, CAT:4657705B) 10 times and incubated at 37 °C for 15 min. The lysates were then pre-cleared by centrifugation at 1,000*g* for 5 min in a tabletop centrifuge (Eppendorf), and 200–300 µl of the supernatant was transferred into thick-wall ultracentrifugation tubes (Beckman-Coulter, CAT:343778). Lysates were centrifuged at 37 °C and 150,000*xg* for one hour in an ultracentrifuge (Eppendorf, model MicroUZ CS150FNX with fixed angle rotor model S150AT), after which the pellet and the supernatant were separated and kept on ice. The pellet was depolymerized by the addition of equivalent volume of ice-cold milliQ distilled water containing 10 µM cytochalasin D (Focus Biomolecules*,* CAT:10-2071), after which the pellet was gently agitated by resuspension every 15 min for one hour. Finally, SDS sample buffer (5% v/v SDS, 0.6 mg/ml bromophenol blue, 30% v/v glycerol, 0.3 M Tris-HCl, 16% v/v β-mercaptoethanol, pH 6.8) was added to all the tubes and the samples were separated by SDS-PAGE and western blot as described previously. For HeLa cells, the procedure was similar but adapted in the following manner for adherent cells: the cells were grown to 80%–90% confluency in the wells of a 6-well plate (Corning). They were washed once with PBS and treated with actin-modulating drugs or DMSO in serum- and antibiotic-free high-glucose DMEM (Sigma-Aldrich, CAT: D6429). Post-incubation, the cells were collected by scraping in the F-actin stabilization buffer and passed through the 27G syringe 10 times. The remainder of the procedure was identical as for Jurkat.

### Quantitative polymerase chain reaction (qPCR)

RNA was isolated following the manufacturer’s protocol (Zymo Research Direct-Zol RNA Isolation Kit). In brief, 1 μg of isolated RNA was transcribed into cDNA (by high-capacity cDNA reverse transcription kit, ThermoFisher, #4368814). For each qPCR reaction, 10 ng of cDNA were used and detection was based on SYBR Green reaction mix (ThermoFisher, #4385612). qPCRs were performed on an Applied Biosystem machine (StepOne Plus) using StepOne v1.2.x software and results were analyzed using the comparative CT (ΔΔCT) method. Results were normalized to the expression of glyceraldehyde 3-phosphate dehydrogenase (GAPDH). Primers, synthesized by MicroSynth (Balgach, Switzerland), are shown below:

Human coronin 1 5′-CTGTGCTGTCAACCCTAAGTT-3′ 5′-GTGGGCGCATTCTTGTCCA-3′
Human coronin 2 5′-CAG GCT TCA GCC GAA TGA G-3′ 5′-CGC AGA CGT AGA CCA CAC T-3′
Human coronin 3 5′-GAC CTG GGA TAG TTC CTT TTG TG-3′ 5′-AAT TCG ACC AGT CTT GTG CAG-3′
Human coronin 4 5′-CCC CAC TAC CCA AAA GTC TGC-3′ 5′-CAG GAG GCG ATC TCA AAA TCA TC-3′
Human coronin 5 5′-CAG GAT TGA ACC CAA CTA CCC-3′ 5′-GAG CAC GAG GCA ATG ATG TT-3′
Human coronin 6 5′-AGA CAG GGC GAG TGG ATA AGA-3′ 5′-CAT TGT GTG GAC ACC AGT CAA-3′
Human coronin 7 5′-GCT GCC ATT CAG ACC TAG TCA-3′ 5′-AGT CGC CAG AGT TTT ACC GTC-3′
GAPDH 5′-GGAGCGAGATCCCTCCAAAAT-3′ 5′-GGCTGTTGTCATACTTCTCATGG-3′

### Flow cytometry analysis

For flow cytometry, cells were washed once with FACS buffer (PBS with 2% FBS, 10 mM EDTA, 0.05% sodium azide) and incubated with Lime fixable Live/Dead marker (Thermo Fisher, CAT: L34991, 1:1,000), diluted in FACS buffer, on ice for 20 min. Cells were then washed with FACS buffer, fixed with 4% PFA, pH 6.9 (Sigma-Aldrich, CAT: 1004960700) on ice for 15 min, washed with FACS buffer and permeabilised with permeabilisation buffer (1× FACS buffer with 0.1% saponin) on ice for 15 min. Cells were then distributed in a 96-well U-bottom plate and incubated with primary antibodies for 1 hour at room temperature, washed with permeabilisation buffer and incubated with secondary antibodies for 1 hour at room temperature. The cells were then washed once with FACS buffer, resuspended in fresh FACS buffer and fluorescence was measured with an Attune acoustic cytometer (Thermo Fisher). Data were analyzed with FlowJo. The following antibodies were used: anti-coronin 2 (generated in rabbit, Sigma, CAT: HPA070456, 1:500), anti-coronin 3 (generated in rabbit, Thermo Fisher, CAT: PA5-30479, 1:100), anti-rabbit, conjugated with AlexaFluor488 (generated in donkey, Thermo Fisher, CAT: A21206, 1:500), anti-rabbit, conjugated with AlexaFluor647 (generated in donkey, Thermo Fisher, CAT: A31573, 1:500).

### Genotyping FLAG-Cor1 knock-in mice

Small piece of toes, collected from mouse pups, were lysed with 250 μl tail buffer containing 100 μg/ml Proteinase K at 56 °C (temp) overnight. The supernatant was harvested by centrifugation at 210*g* for 10 min. The gene region harboring the FLAG tag-encoding region was amplified by PCR (40 cycles of 30 s at 95 °C, 30 s at 54 °C, and 60 s at 72 °C on an Eppendorf Mastercycler). The presence of the FLAG tag was analyzed using the FLAG_ forward primer and mCor1_reverse primer followed by 2% agarose gel electrophoresis. For Sanger Sequencing, the gene region harboring the FLAG tag was amplified using mCor1_forward primer and mCor1_reverse primer. For primer sequences, see the resource table.

### Quantification of T lymphocytes in mice

The protocol was adapted from a previously published method [112]. Thymus, spleen, and pooled inguinal + axillary lymph nodes were harvested from wild type, FLAG-KI or coronin 1-deficient mice. Single-cell suspensions were prepared by mechanically dissociating the organs through a 150 µm polyamide mesh (Sefar Nitex, cat. no. 03-150-38) using a plunger. Cells were subsequently counted using the Attune flow cytometer. The single-cell suspensions were then stained with an antibody mixture containing Fixable Live/Dead marker (Thermo Fisher, cat. no. L10119, 1:1000), anti-CD3 (BioLegend, cat. no. 100216, 1:150), anti-CD4 (BioLegend, cat. no. 100559, 1:150), and anti-CD8 (BioLegend, cat. no. 100780, 1:150). Staining was performed for 30 min at 4 °C. After staining, cells were washed with FACS buffer, and the pellets were resuspended in FACS buffer. Flow cytometric analysis was performed using either a BD LSR Fortessa or an Attune CytPix flow cytometer. Flow cytometry data were analyzed using FlowJo software (v10 or v11). Total cell numbers per organ were determined based on the population frequency of the indicated subsets.

### Plasmid cloning

Plasmids mEmerald-Coronin1B-N-10, mEmerald-Coronin1B-C-10 and mEmerald-C1 were obtained from Addgene (plasmids by Michael Davidson, Addgene ID #54050, #54049 and #53975, respectively). Plasmid expressing mEmerald-coronin 1 and coronin 1-mEmerald were cloned by replacing the coronin 2 (coronin 1B) sequence of mEmerald-Coronin1B-C-10 and Coronin1B-N-10, respectively, with coronin 1. Coronin 1 cDNA was amplified from plasmid pEGFP-N1::hCor1 #3 available in the laboratory (using primers fwd: ATATAGATCTGCCACCATGAGCCGGC and rev: CGGTGGATCCCTACTTGG). Amplified coronin 1 cDNA and mEmerald-Coronin1B-C-10 were restricted using BglII and BamHI (New England Biolabs, CAT: R0144S; CAT: R0136S), while amplified coronin 1 cDNA and mEmerald-Coronin1B-N-10 were digested using NheI-HF and BamHI-HF (New England Biolabs, CAT: R3131S; CAT: R3136S). Ligation was performed using T4 DNA ligase (Promega, CAT: M180B). All the DNA separation and isolation steps were done after 1% agarose gel electrophoresis using QIAquick gel extraction kit (Qiagen, CAT: 28704).Plasmid expressing coronin 3-mEmerald was cloned by replacing the coronin 2 (coronin1B) sequence of mEmerald-Coronin1B-N-10 with coronin 3. Coronin 3 cDNA was amplified from plasmid pPR-CMV-hCor3-FLAG (excluding the FLAG-tag) available in the laboratory (using primers fwd: ATATGCTAGCGCCACCATGAGGCGAGTGGT and rev: ATATGGATCCCCGCTACCGCCGGCTGCTATCTTTGCC). Amplified coronin 1 cDNA and mEmerald-Coronin1B-N-10 were digested using NheI-HF and BamHI-HF (New England Biolabs, CAT: R3131S; CAT: R3136S). Ligation was performed using T4 DNA ligase (Promega, CAT: M180B). All the DNA separation and isolation steps were done after 1% agarose gel electrophoresis using QIAquick Gel Extraction Kit (Qiagen, CAT: 28704). All plasmids used in the study were validated by full plasmid sequencing at Microsynth (Switzerland).

### Cell transfections

Cells were transfected either using the TurboFect transfection reagent (Thermo Scientific, CAT: R0531) or Neon Transfection System (Thermo Fisher, CAT: 10431915). When using the TurboFect transfection reagent, cells were transfected according to the manufacturer’s protocol in a 6-well plate (Corning, CAT: 3506), with 12 µl of the TurboFect reagent and 2.5 µg of plasmid DNA added per well (volume total of 3 ml per well, out of which 1 ml transfection volume). After 24 h, cells were trypsinized and seeded onto microscopy glass coverslips (VWR, CAT: 631-0150). Cells were used in experiments 48 h post-transfection without selection.

When using the Neon Transfection System, Jurkat cells were transfected according to the manufacturer’s protocol for suspension cells in a 10 µl tip and seeded in a 24-well plate (Corning, CAT: 3524). Electroporation conditions used were 1,400 V for two pulses lasting 20 ms each. The cells were re-seeded into a T-25 flask (Corning, CAT: 430639) after 24 h. The cells were used in experiments 48 h post-transfection without selection. HeLa cells were transfected according to the manufacturer’s protocol for adherent cells in a 10 µl tip and seeded in a 6-well plate (Corning, CAT: 3506). Electroporation conditions used were 1,005 V for two pulses lasting 35 ms each. The cells were trypsinized and re-seeded onto microscopy glass coverslips (VWR, CAT: 631-0150) in a 24-well plate (Corning, CAT: 3524) after 24 h and used in experiments 48 h post transfection without selection.

### Immunofluorescence microscopy

HeLa cells (30,000 cells) and mouse embryonal fibroblasts (10,000 cells) were seeded in full growth medium (DMEM, 10% FBS, 1% Pen-Strept) onto microscopy glass coverslips (VWR, CAT: 631-0150) at the bottom of a 24-well plate (Corning, CAT: 3524) well and left overnight to attach in a humidified incubator with 5% CO_2_ atmosphere. Mel JuSo (30,000 cells) was seeded identically, in its own full growth medium (10% heat-inactivated FBS in RPMI-1640, 1% Pen/Strept); same for J774.A1 (30,000 cells) (10% heat-inactivated FBS in high-glucose DMEM, 1% Pen/Strept).

The coverslips were coated with CellTak (Corning, CAT: 354240) according to the manufacturer’s protocol. Jurkat cells were seeded (50,000 cells) onto CellTak-coated microscopy glass coverslips on the bottom of a 24-well plate (Corning, CAT: 3524) well immediately before fixation. For spread Jurkat samples, the cells were allowed to sediment in the incubator and additionally centrifuged at 200*g* at room temperature for one minute to spread them laterally. Mouse T cells were seeded onto CellTak-coated coverslips as described for Jurkat cells.

Cells were fixed either using 4% methanol-free formaldehyde (referred to as PFA fixation) or ice-cold (−20 °C) methanol (Sigma-Aldrich, CAT: 32213). Blocking solution consisted of 3% BSA, 0.1% Tween20 in PBS. Washing buffer was 0.1% Tween20 in PBS. For membrane control stainings, cells were incubated in DMEM containing 3 µg/ml CF568-WGA (Biotium) for 15 min prior to fixation at 37 °C.

For PFA fixation, the cells were incubated in 4% PFA (Sigma-Aldrich, CAT:158127; Sigma-Aldrich, CAT: D8537) for 15 min at room temperature. They were then washed once with PBS (Sigma-Aldrich, CAT: D8537) and incubated for 20 min in permeabilisation buffer (0.2% Tween20 in PBS). For blocking, the cells were incubated in blocking solution at room temperature for 1 h while gently shaking on a rocking platform. Primary antibody incubation was done overnight at 4 °C in primary antibody-containing blocking solution. The following primary antibodies were used in the experiments: anti-coronin 1 (mouse monoclonal cl. 4G10, Abnova, 1:1,000; rabbit polyclonal serum 1,002 produced in house 1:1,000), anti-coronin 2 (mouse monoclonal SC-271445, cl. G-8, Santa Cruz Biotechnology, 1:500; rabbit polyclonal, Thermo CAT: PA564443, 1:1,000), anti-GFP (chicken polyclonal, Millipore, 1:1000), and anti-S6 ribosomal protein (rabbit polyclonal, Cell Signaling Technology, 1:1,000). After the primary antibody incubation, the cells were washed in washing buffer three times 5 min while gently shaking as above. Secondary antibody staining was done for 1 h at room temperature in secondary antibody-containing blocking solution while gently shaking in the dark. Phalloidin-647I (Abnova, 1:10,000) and DAPI (Sigma, 0.1 µg/µl) were added to the secondary antibody staining solution. Following secondary antibodies were used in the experiments: goat anti-mouse AF488 (Thermo Fisher, 1:1,000), goat anti-mouse AF568 (Thermo Fisher, 1:1,000), goat anti-chicken AF568 (Thermo Fisher, 1:1,000), donkey anti-rabbit AF488 (Thermo Fisher 1:1,000). Finally, the cells were washed in washing buffer for three times (5 min, room temperature), after which the coverslips were removed from the 24-well plate and mounted onto microscopy glass slides with a droplet (~10 µl) of mounting medium (Vector Labs, CAT: H-1700). The slides were left in the dark at room temperature for 30 min for the mounting medium to solidify.

For methanol fixation, the cells were incubated on ice for 5 min in ice-cold (−20° C) methanol. They were then washed once with PBS and incubated in blocking solution, with the remaining staining procedure performed as described above for 4% PFA. Primary antibodies used in the experiments were anti-coronin 3 (rabbit polyclonal, Invitrogen, 1:1,000), mouse anti-actin (hybridoma, cl. JLA20, 1:50) and anti-GFP (chicken polyclonal, Millipore, 1:1,000). The following secondary antibodies were used: donkey anti-mouse AF647 (Life Technologies, 1:1,000), goat anti-chicken AF568 (Thermo Fisher, 1:1,000), donkey anti-rabbit AF488 (Thermo Fisher, 1:1,000). Fluorophore-coupled phalloidin was not used in methanol-fixed samples.

The samples for phalloidin-647I-fluorescence retrieval were fixed using 4% PFA for 15 min followed by permeabilization for 20 min (both at room temperature). Samples were incubated in blocking solution for 1 h while gently shaking (on a rocking platform at room temperature and subsequently in phalloidin-647I-containing blocking solution (1:10,000) for 1 h while gently shaking as above. The cells were washed in washing buffer three times 5 min. at room temperature and covered in the mounting medium.

### F-actin depolymerization

HeLa cells (30,000 cells) were seeded in full medium (DMEM, 10% FBS, 1% Pen-Strept) onto microscopy glass coverslips (VWR, CAT: 631-0150) at the bottom of a 24-well plate (Corning, CAT: 3524) and left overnight to attach in a humidified incubator with 5% CO_2_ atmosphere. The medium was then aspirated and replaced with DMEM (Sigma-Aldrich, CAT: D6429) containing 2 µM latrunculin A (Focus Biomolecules, CAT: 10-2254), upon which the cells were returned to the 37 °C/5% CO_2_ incubator for 1 hour. Afterwards, the medium was aspirated and cells fixed as described for immunofluorescence microscopy.

For actin repolymerization experiments, the above procedure was repeated until the point when the latrunculin A-containing medium was replaced with full medium, after which the cells were returned to the incubator. Media were then aspirated and cells fixed as described for immunofluorescence microscopy at 5, 10, 20, and 40 min time points post-washout.

For 647I-phalloidin (Abnova, CAT: U0298) fluorescence retrieval experiments, HeLa cells (15,000) were seeded in wells of black 96-well plate (Corning, CAT: 3603). F-actin depolymerization and washout were done as described in the “Immunofluorescence microscopy” section, with only difference being the used 4 µM LatA concentration. Instead of the mounting medium, 90% v/v solution of glycerol and PBS was used.

Jurkat cells were collected by centrifugation at 200*g* and counted using a Neubauer chamber. They were then resuspended in RPMI medium (Sigma-Aldrich, CAT: R8758) containing 2 µM latrunculin A (Focus Biomolecules, CAT: 10-2254). The cells were then returned to 37 °C/5% CO_2_ for 1 hour. Afterwards, Jurkat cells were resuspended and seeded (50,000 cells) onto CellTak (Corning, CAT: 354240)-coated microscopy glass coverslips (VWR, CAT: 631-0150) at the bottom of a 24-well plate (Corning, CAT: 3524) well and fixed as described for immunofluorescence microscopy.

For protein kinase C activation/inhibition assays, the cells were incubated for one hour in the presence and absence of the following compounds in their respective serum- and antibiotic-free media, or calcium- and magnesium-free PBS for J774.A1 macrophages in the presence or absence of the following compounds: 10 µM PMA (Tocris, CAT: 1201), 20 µM chelerythrine (Tocris, CAT: 1330), 50 µM final concentration of pervanadate (Merck, CAT: 567540-5GM, 1:1 diluted orthovanadate stock using 30% hydrogen peroxide by Merck, CAT: K40677109, prepared fresh before every experiment). All of the conditions were also performed with the addition of 2 µM LatA, or equivalent volume of DMSO [113]. The rest of the experiments was as described in the immunofluorescence section.

### Measurement of phalloidin-based fluorescence retrieval

The samples for phalloidin fluorescence retrieval experiments were recorded using a Tecan Spark plate reader, with the following filters:

DAPI Ex. 340/20; Em. 510/10
Phalloidin-647I Ex. 648/10 (monochromator); Em. 680/30.

### Measurement of cAMP levels by immunocompetition assay

Measurement was done using the HTRF cAMP Gs Dynamic kit (Perkin Elmer, CAT: 62AM4PEB) according to the manufacturer’s instructions. Briefly, HeLa cells were harvested by 5 mM EDTA in DPBS (Sigma-Aldrich, CAT: D8537), transferred into FACS buffer (1% BSA in DPBS) and counted by flow cytometry. The cells were incubated for 30 min at 37 °C/5% CO_2_ in 500 µM IBMX (Tocris, CAT: 2845) in FACS buffer with and without the presence of 2 µM LatA (Focus Biomolecules, CAT: 10-2254). 30,000 cells were seeded per well of a 384-well plate (PerkinElmer, CAT: 6008280) and stimulated with 50 µM forskolin (AG Scientific, CAT: F-1026) in FACS buffer or DMSO (Sigma, CAT: D2438) for 30 min at 37 °C/5% CO_2_. Afterwards, the antibody reagents from the kit were added and incubated for 1 h at room temperature. Fluorescence was measured on the Tecan Spark plate reader using Ex 320/25 Em 620/10 filters for the donor and Ex 320/25 Em 665/8 filters for the acceptor channels.

### Measurement of cAMP levels by ratiometric FRET microscopy

Plasmid for the expression of Epac-S-H187 [84] was obtained from Addgene (#170348). Cells were transfected as described using the Neon Transfection System (Thermo Fisher, CAT: 10431915). Cells were seeded inside Lab-Tek II #1.5 microscopy glass bottom chambers (Thermo Fisher, CAT: 155409) 24 h post-transfection. Imaging was done 48 h post-transfection with temperature control at 37 °C on the Zeiss CellObserver.Z1 widefield microscope through a 40× objective (oil fluor M27, NA 1.3, CAT: 420260-9900-000). Cell medium was replaced by 500 µM IBMX (Tocris, CAT: 2845) with or without 2 µM LatA (Focus Biomolecules, CAT: 10-2254) in DPBS (Sigma-Aldrich, CAT: D8537) and incubated for 30 min. Imaging was begun for 30 s prior to the addition of forskolin (AG Scientific, CAT: F-1026) to ensure stable fluorescence and continued in 10 s intervals until the end of 10 min. Forskolin (AG Scientific, CAT: F-1026) was added as a 2× solution (300 µl into 300 µl of the pre-incubation solution per chamber well) for the final concentration of 25 µM, and the time point at which forskolin was added was considered time point zero (*t* = 0 s) for further analysis.

### Imaging of cells

Fixed microscopy samples were imaged using the Zeiss Cell Observer.Z1 inverted widefield microscope through the 63× 1.4NA oil plan-apochromatic objective (Zeiss, CAT: 420782-9900). Z-stacks of 40–80 slices with 0.2 µm distance between slices and 103 nm laterally per pixel were acquired. Brightfield images were acquired using HAL 100 TL Halogen lamp (Zeiss, CAT: 423000-9901-000). Fluorescence images were acquired using the X-Cite Xylis Lamp (Excelitas Technologies, CAT: XT720S). Camera used was ORCA-Fusion Digital CMOS camera (Hamamatsu Photonics, CAT: C14440-20UP).

Filter cubes used:

1. For AF647 and 647I - BP 640/30, FT 660, BP 690/50
2. For DAPI – BP 370/40, FT 395, BP 445/50
3. For GFP and AF488 – BP 474/28, BP 525/50
4. For AF568 and WGA-555 – BP 550/25, FT 570, BP605/70
5. For CFP – BP 436/25, FT455, BP 480/40
6. For YFP-FRET – BP 433/24, FT 458, BP 562/40

The software used to operate the microscope was ZenBlue 3.2 (Zeiss).

For super-resolution images of mouse T cells, the samples were imaged using the Zeiss Lattice SIM3D inverted microscope through the 40 × 1.4NA oil plan-apochromatic objective (Zeiss, CAT: 420762-9900-000). Images were acquired in z-stacks of 59 slices 0.12 µm distance between slices and 36.5 nm laterally per pixel.

### Image analysis

Images were assembled, organized and arranged into figures using OMERO (OME) and deconvolved using Huygens Remote Manager, Huygens Essential, or Huygens Professional (SVI) with a measured point-spread function (classical maximum-likelihood estimation, 500 cycles, stopping threshold 0.001) and corrected for chromatic shift. Export image format was ICS2. For presentation purposes, the images were imported and arranged in OMERO.

For colocalization analysis, downloaded images in image cytometry standard (.ics) format were cropped and regions of interest drawn by hand in Fiji (ImageJ) to mark single cells. Cropped images were saved in.tiff format and analyzed further.

Analysis was performed for every image using a Fiji macro as follows: in brief, the brightness and contrast settings were reset to global z-stack maximum and minimum values for each channel, and the bit depth was converted from 32-bit to 16-bit. BIOP-JACoP (PTBIOP, EPFL Lausanne) plugin was then launched and an automatic Otsu threshold applied to the actin channel per stack histogram, and a manual threshold of 5,000 determined from respective knockout cells applied to the coronin channel. Pearson’s and thresholded Manders’ coefficients were generated. Report windows and.csv files containing the data were exported and saved. Afterwards, the numeric data were assembled, analyzed and visualized in GraphPad Prism.

Phalloidin-647I fluorescence retrieval was quantified by expressing the ratio of phalloidin and DAPI channel fluorescence averages for each well. The values were normalized by subtracting the ratio values obtained from time point 0′ plate from the corresponding wells in every other plate. The values were assembled and plotted in GraphPad Prism.

Ratiometric FRET images were generated and measured from cropped raw images using an ImageJ macro as follows: channels were split, with the CFP donor channel duplicated once more to generate a binary mask by applying the Otsu auto threshold. Selection was created from the mask and restored on the original donor and acceptor channel images, for which mean intensity values were measured and ratioed. The values were pooled in Graph Pad Prism and analyzed further. Pixel values outside the selection were set to zero. This was repeated for every time point of the timelapse images. After the complete measurement of the timelapse, donor and acceptor channel stacks were ratioed and a ‘Fires’ LUT was applied to the resulting image. Minimum display range values were set below the average at the time point at which forskolin was added and the maxima were unified at 1.4 to calibrate the LUT to absolute CFP/FRET changes.

### LC–MS analysis

Hela cells deficient in coronin 2, coronin 3 and corresponding wild-type cells were subjected to LC–MS analysis. Three to 4 replicates of one clone per each genotype were analyzed. Dried peptides were resuspended in 0.1% aqueous formic acid and subjected to LC–MS/MS analysis using an Orbitrap Exploris 480 Mass Spectrometer fitted with an Vanquish Neo nano-LC (both Thermo Fisher Scientific) and a custom-made column heater set to 60 °C. Peptides were resolved using a RP-HPLC column (75 μm × 30 cm) packed in-house with C18 resin (ReproSil-Pur C18–AQ, 1.9 μm resin; Dr. Maisch GmbH) at a flow rate of 0.2 μL/min. The following gradient was used for peptide separation: from 4% B to 10% B over 7 min to 30% B over 76 min to 40% B over 7 min to 95% B over 1 min followed by 10 min at 95% B. Buffer A was 0.1% formic acid in water and buffer B was 80% acetonitrile, 0.1% formic acid in water.

The mass spectrometer was operated in DIA mode with a cycle time of 3.5 seconds. MS1 scans were acquired in the Orbitrap in centroid mode at a resolution of 60,000 FWHM (at 200 *m*/*z*), a scan range from 425 to 625 *m*/*z*, normalized AGC target set to 300% and maximum ion injection time mode set to 50 ms. MS2 scans were acquired in the Orbitrap in centroid mode at a resolution of 30,000 FWHM (at 200 *m*/*z*), precursor mass range of 430–670, quadrupole isolation window of 5 *m*/*z* without window overlap, a defined scan range of 200–1,800 *m*/*z*, normalized AGC target set to 3,000% and a maximum injection time set to auto (55 ms). Peptides were fragmented by HCD (Higher-energy collisional dissociation) with stepped collision energy set to 22,26,30% and one microscan was acquired for each spectrum.

### Protein identification and quantification

The generated raw files were searched against a predicted human library (A reviewed-entry FASTA w/o isoforms was downloaded from Uniprot on 2021-11-22, 1 allowed missed cleavage, C+57 as fixed modification was set) using DIANN (PMID: 31768060) (v1.8.1) and default settings with a few changes: MBR was activated, Protein names were selected for Protein inference and Neural network classifier were set to Single-pass mode. Quantitative results obtained from the report file were further analyzed using the MSstats R package v.4.7.3. (https://doi.org/10.1093/bioinformatics/btu305) [114]. Linear mixed model as implemented in MStats R package was used to identify significantly changed proteins. The obtained *p*-values was corrected for multiple testing using Benjamini–Hochberg procedure as implemented in MStats package. The power analysis was performed using pwr R package (citation: Champely S (2023). *pwr: Basic Functions for Power Analysis*. R package version 1.3-0, https://github.com/heliosdrm/pwr.). In all three (Coronin 1, 2 and 3 KO) datasets >99% of identified proteins had a power of at least 80% for detecting a 2-fold change with adjusted *p*-value 0.01. Log2 fold changes of protein abundance and corresponding adjusted *p*-values between coronin 2 and coronin 3-deficient cells in comparison to wild-type HeLa cells are listed in S2 and S3 Data.

All mass spectrometry files associated with this manuscript are accessible at MassIVE (https://massive.ucsd.edu) under accession number MSV000097230. To review: go to https://massive.ucsd.edu, login as: MSV000097230_reviewer, password: coronins.

### RNA-seq

Wild-type, coronin 1- or 3-deficient Jurkat cells were subjected to transcriptomic analysis. One clone per each genotype were analyzed in 3 replicates. RNA extraction was perfumed using the RNeasy Micro kit (QIAGEN, catalogue 74004). Library preparation was performed starting from 200 ng total RNA, using the TruSeq Stranded mRNA Library Kit (Cat# 20020595, Illumina, San Diego, CA, USA) and the TruSeq RNA UD Indexes (Cat# 20022371, Illumina, San Diego, CA, USA). Fifteen cycles of PCR were performed. Libraries were quality-checked on the Fragment Analyzer (Advanced Analytical, Ames, IA, USA) using the Standard Sensitivity NGS Fragment Analysis Kit (Cat# DNF-473, Advanced Analytical). Average concentration was 153 ± 13 nmol/L and average library size was 345 ± 6 base pairs. Samples were pooled to equal molarity. The pool was quantified by Fluorometry using the QuantiFluor ONE dsDNA System (Cat# E4871, Promega, Madison, WI, USA). Libraries were sequenced Paired-End 51 bases (in addition: 8 bases for index 1 and 8 bases for index 2) setup using the NovaSeq 6000 instrument (Illumina) and the SP Flow-Cell.

### Transcriptomic data analysis

Both raw and trimmed (4-nucleotide sliding windows, Phred threshold of 15, using Trimmomatic [115] reads were aligned to the Homo sapiens genome (Ensembl 104, GRCh38) using STAR [116]. Trimmed reads alignments were kept on the basis of the number of uniquely aligned reads. Read counts were summarized using the “--quantmode GeneCounts” option of STAR, with the appropriate strandedness. Differential expression analysis was performed by DESeq2 [117] using a model incorporating the effects of genotype with a cutoff of adjusted *P*-values <0.01, using the ashr shrinkage estimator for log fold changes [118]. The power analysis was performed with RNA-Seq Power Calculator (DESeq2 Model) online tool [119]. The estimated power for all three RNA-seq datasets was above 85% for detecting a 2-fold change with adjusted *p*-value 0.01.

Log2 fold changes of counts per gene and corresponding adjusted *P*-value between coronin 1 and coronin 3 deficient in comparison to wild -ype Jurkat cells are listed in S2 Data for coronin 1 and 3, respectively. Raw and processed RNA-seq data are available under GEO accession number: GSE292209. To review: go to https://www.ncbi.nlm.nih.gov/geo/query/acc.cgi?acc=GSE292209. Enter token: szyxmmoervctzsd into the box.

### Bioinformatic analysis

Transcriptomic and proteomic data used in Fig 5 were either described before [13,30] or generated and analyzed as described above. Volcano plots were generated using R programming software (https://www.R-project.org). Lists of 20, 40 and 30 actin associated proteins reported to interact with either coronin 1, 2 or 3, respectively, were manually assembled based on PubMed available literature on coronin proteins. Each list was used for analyzing the ablation experiments of corresponding coronin protein. Proteins are listed in S3 Table. S4 Table provides references to papers describing their connection to coronins.

### Bibliometric analysis

For the bibliometric analysis, a systematic search of PubMed was performed using an extensive set of search terms reflecting the diverse nomenclature of the coronin protein family (last accessed March 4, 2026) from the different species indicated in the S1 Table, S3 and S4 Data, and Fig 6. Although every effort was made to ensure completeness, the possibility of missed publications cannot be fully excluded due to indexing and naming variability. The authors welcome notification of any missed publications for inclusion in the regularly updated repository at https://www.coronin.org. Publications were manually screened whether or not coronin/Crn1-actin interaction data are presented and whether endogenous or recombinantly expressed and/or tagged proteins were analyzed. Only interaction reported from biochemical analysis (and not imaging-based colocalization) was scored. Actin interaction was defined as (i) co-sedimentation of coronin/Crn1 proteins with actin in vitro; (ii) co-immunoprecipitation from cell lysates; (iii) cosedimentation following Triton X100 lysis in case actin depolymerizing agents (e.g., LatA/B) were included; and (iv) cryo-EM analysis. In the summary pages of S3 and S4 Data and in the bar graphs in Fig 6 papers describing both endogenous and recombinantly expressed and/or tagged proteins are counted multiple times. The summary sheets in the individual S3 and S4 Data tally the single publications. The PubMed entry of each publication can be accessed by clicking on the ID number listed in the PMID column of each table.

### Quantification and statistical analysis

All statistical analyses were done in GraphPad Prism. For colocalization, z-stacks containing regions of interest with one cell each were analyzed for each condition and the median values were plotted. To assess the significance of HeLa, Jurkat and mouse T cells Pearson’s and Manders’ coefficients, Student *t* test was used.

The details of all statistical analyses are found in Figure captions and the significance is denoted with asterisks in the figure itself.

For phalloidin-647I fluorescence retrieval quantification, 12 wells of a 96-well plate were imaged for each condition (*n* = 12) and the median values were plotted. Two-way ANOVA was used to assess the significance relative to wild-type samples different conditions on each time point using multiple comparisons relative to wild-type.

For immunocompetitive kit-based cAMP measurements, two-way ANOVA was used to assess the significance relative to wild-type samples of different conditions on each time point using multiple comparisons relative to wild-type.

Adobe Illustrator was used to assemble the figures.

## Supporting information

S1 Fig Validation of coronin 2 and 3-deficient HeLa cells and antibodies for immunofluorescence. **(A)** Lysates of the indicated Hela cells were probed with anti-coronin 2 (mouse mAb cl.5–7.1) and anti-coronin 3 (mouse mAb K6-444) antibodies (upper panels), and with anti-actin antibodies as a loading control (lower panels). Raw images of blots can be found in S1 Raw Images. **(B)** Coronin 2 and coronin 3 were stained intracellularly in wild-type, coronin 2- and coronin 3-deficient cells using anti-coronin 2 (rabbit, Sigma,HPA070456) and anti-coronin 3 (rabbit, Thermo Fisher, CAT: PA5-30479) antibodies and fluorescence was measured by FACS. Gating strategy and.fcs files can be found in S1 Data. **(C)** Mean expression of coronin proteins in wild-type HeLa cells was examined by qPCR and plotted relative to coronin 7. Error bars indicate standard deviation. Numeric values can be found in S1 Data. **(D)** Wild-type (upper panels) or coronin 2-deficient (lower panels) HeLa cells were fixed with PFA, permeabilised, stained with anti-coronin 2 antibody (mouse mAb cl. G-8, Santa Cruz, cat. # sc-271445, 1:500 dilution) and 647I-phalloidin to label F-actin, followed by image acquisition through a 63× objective (oil, NA 1.4, plan-apochromat), with single slices shown. Bars are 10 μm. **(E)** Wild type (upper panels) or coronin 3-deficient (lower panels) HeLa cells were fixed with methanol, stained with anti-coronin 3 antibody (rabbit pAb, Invitrogen, # PA5-30479, 1:1,000 dilution) and anti-actin antibody, followed by image acquisition through a 63× objective (oil, NA 1.4, plan-apochromat), with single slices shown. Bars are 10 μm.
(TIF)

S2 Fig Localization of coronin 2 and GFP in mouse embryonal fibroblasts. **(A)** Mouse embryonal fibroblasts (MEFs) that were fixed in PFA, permeabilised, and stained with anti-coronin 2 antibodies (PA5-64443, lower panels). 647I-phalloidin was used to label F-actin. Images were deconvolved and single slices are shown. Enlarged insets from white rectangles are shown underneath each image. Bars are 50 μm for images and 10 μm for insets. **(B)** Mouse embryonal fibroblasts (MEFs) transfected with a plasmid encoding for GFP that were fixed in PFA, permeabilised and stained with 647I-phalloidin to label F-actin. Images were deconvolved and single slices are shown. Enlarged insets from white rectangles are shown underneath each image. Bars are 50 μm for images and 10 μm for insets.
(TIF)

S3 Fig Colocalization supplement for Fig 1. **(A)** Representative Jurkat cells transfected with a plasmid encoding coronin 1-GFP (upper) or GFP (lower) seeded by sedimentation and then fixed in PFA, permeabilised, and stained with anti-GFP antibodies. 647I-phalloidin was used to label F-actin. Images were deconvolved and single slices are shown. Bars are 10 μm. **(B)** Representative HeLa cells transfected with a plasmid encoding coronin 2-GFP (upper) or GFP (lower) fixed in PFA, permeabilised, and stained with anti-GFP antibodies. 647I-phalloidin was used to label F-actin. Images were deconvolved and single slices are shown. Insets from white rectangles are shown to the right of the corresponding image. Bars are 10 μm in images and 5 μm in insets. **(C, D)** Representative wild-type Jurkat (C) and HeLa (D) cells were stained for membrane using WGA-568, fixed in PFA, permeabilised and stained for S6 ribosomal protein using antibodies, as well as F-actin using 647I-phalloidin. Images were deconvolved and single slices are shown. Bars are 10 μm. **(E, F)** Median Pearson’s correlation coefficients calculated between endogenous coronin 1 in Jurkat (E) or coronin 2 in HeLa (F) cells, their respective GFP fusion proteins and control stains relative to F-actin. **** *p* < 0.0001, ns - nonsignificant (Student *t* test relative to endogenous coronin). Numeric values can be found in S1 Data. **(G)** Representative wild-type HeLa cell transfected with plasmid expressing GFP, fixed in PFA, permeabilised and immunostained for coronin 2, as well as F-actin using 647I-phalloidin. GFP was imaged by its intrinsic fluorescence. Insets from white rectangles are enlarged beneath the corresponding channel images. White arrowhead in the inset points to an example of a filament prominent in phalloidin and GFP channels, but not coronin 2 channel. Bars are 10 μm in images and 5 μm in the insets.
(TIF)

S4 Fig Effect of GFP-tagging on overexpressed coronin 1 localization in Mel JuSo cells. **(A)** Representative wild-type Mel JuSo cells (upper panels), and Mel JuSo stably overexpressing untagged murine coronin 1 (middle panels) or murine coronin 1 tagged with EGFP (lower panels) were fixed in PFA, permeabilised, and stained using coronin 1 antibodies. 647I-phalloidin was used to label F-actin. Images were deconvolved and single slices are shown. Enlarged insets from white rectangles are shown to the right of each image. Bars are 10 μm for images and 5 μm for insets. **(B)** Median Pearson’s correlation coefficients calculated between coronin 1 or coronin 1-EGFP relative to F-actin in cells stained as in (A). **** *p* < 0.0001 (Student *t* test). Numeric values can be found in S1 Data. **(C)** Median thresholded Manders’ coefficients calculated for coronin 1 or coronin 1-EGFP relative to F-actin in cells stained as in (A). **** *p* < 0.0001 (Student *t* test). Numeric values can be found in S1 Data.
(TIF)

S5 Fig Construction and validation of knockin mice with the octapeptide FLAG inserted into the coronin 1-encoding genomic locus in mice. **(A, B)** Strategy for inserting FLAG into the N-terminal of mouse coronin 1. A: Diagram of CRISPR/Cas9 electroporation to generate germline-modified mice. B: Sanger sequencing of the region encompassing the gRNA targeting site from both wild-type (WT) and FLAG knock-in (KI) mice. The start codon, gRNA, PAM sequence (protospacer adjacent motif), and FLAG tag are indicated. **(C)** Strategy used for FLAG knock-in (KI) into the murine genomic locus encoding for coronin 1. Modified from [103]. **(D)** Analysis of FLAG-coronin 1 protein expression by Western blotting. Coronin 1 (WT) and FLAG-coronin 1 (KI) proteins were detected by coronin 1 antibodies (Abnova, cl. 4G10), with lysates from coronin 1-deficient T cells for comparison (KO). Accompanying Ponceau stain of the same membrane is shown to the right of the blot. See also S1 Raw Images. **(E)** Expression of FLAG in thymus, splenocytes, lymph nodes (LN), and blood cells from wild-type and FLAG-knock in mice measured by flow cytometry. Cells were first fixed and permeabilised, subsequently incubated with primary (rabbit anti-FLAG) and secondary (anti-rabbit Alexa Fluor 568) antibodies. For gating strategy and.fcs files see S1 Data.
(TIF)

S6 Fig Relocalization of endogenous coronin 1 and 2 is dependent on protein kinase C activity, but not polymerization state of actin. **(A–C)** Representative images of Jurkat (A), J774.A1 (B) and HeLa cells (C) left untreated or treated with indicated chemicals diluted in respective serum-free medium in the absence or presence of LatA for 1 h at 37 °C and 5% CO_2_. The cells were then fixed and stained as for Fig 1 for HeLa and Jurkat, while the J774.A1 macrophages were probed for coronin 1 using rabbit polyclonal serum (1:1,000 dilution) raised in-house followed by anti-rabbit secondary antibodies coupled to AlexaFluor-488. Brightfield images were also acquired. Images were acquired, processed, handled and presented as in Fig 1. Bars are 10 µm in all images.
(TIF)

S7 Fig Ablation of expression of coronin 2 and 3 in HeLa cells does not result in compensatory increase in other coronin protein levels. Log2 fold changes of all coronin proteins detected by mass spectrometry in four HeLa cell clones deficient in both coronin 2 and 3 were plotted relative to wild-type. Corresponding adjusted *p* values for each of the conditions are shown underneath the plot. Lack of plotted symbols means the proteins were not detected in any of the samples.
(TIF)

S8 Fig Analysis of tagged coronin protein association with F-actin in cells. **(A, B)** Jurkat (A) or HeLa (B) cells were transfected with plasmids to ectopically express the indicated fluorescent protein constructs and treated as in Fig 4A and 4B. Arrowheads indicate the position of the degradation product observed in C-terminally tagged coronin constructs. For raw images of blots see S1 Raw Images.
(TIF)

S9 Fig Production of cAMP in wild-type and coronin-deficient HeLa cells upon forskolin stimulation with and without an intact actin cytoskeleton. **(A, B)** Wild-type, and coronin 2 and/or 3-deficient HeLa cells were incubated in 500 µM pan-phosphodiesterase inhibitor IBMX in the absence (A) or presence of 2 µM LatA (B) for 30 min, after which they were stimulated with either DMSO (vehicle) or 50 µM forskolin for 30 min. Concentration of cAMP was measured using a FRET-based immunocompetition kit, where donor and acceptor fluorescence were measured on a Tecan Spark plate reader. Calculated fold cAMP concentration changes upon forskolin stimulation relative to vehicle control are shown for each cell line. Bars represent standard deviation and statistical significance was examined by one-way ANOVA. ns, nonsignificant; ** *p* < 0.01, *** *p* < 0.001, **** *p* < 0.0001. Numeric values can be found in S1 Data.
(TIF)

S1 Table Coronin proteins_tagged fusions and localization. (XLSX)

S2 Table Coronin 2 and 3 antibody testing. (PDF)

S3 Table Actin modulating proteins_UNIPROT list. (XLSX)

S4 Table Literature referring to coronin actin modulation. (PDF)

S1 Raw Images File containing all the annotated and uncropped images of blots in the main and supplementary figures. (PDF)

S1 Data Excel spreadsheet with all the numeric data underlying main and supplementary figure plots. (XLSX)

S2 Data Excel spreadsheet containing plotted genes in the transcriptomic and proteomic analyses in the paper. (XLSX)

S3 Data Excel spreadsheet containing underlying bibliography for the analysis of non-mammalian coronin literature. (XLSX)

S4 Data Excel spreadsheet containing underlying bibliography for the analysis of mammalian coronin literature. (XLSX)

S1 Movie Supplementary to Fig 7A and 7C showing time-lapse analysis of wild-type (WT) and coronin 2 and 3-deficient HeLa cells expressing the Epac-S-H187 cAMP-reactive FRET reporter in the absence or presence of 2 µM LatA following forskolin stimulation. (AVI)

S1 FACS Data Compressed archive containing gating strategies and .fcs files used in relevant main and supplementary figures. Provided via Zenodo. DOI: https://doi.org/10.5281/zenodo.20745990.
(7z)

## Abbreviations

DKO: double knockout
DSB: double strand break
GAPDH: glyceraldehyde 3-phosphate dehydrogenase
LatA: latrunculin A
MEF: murine embryonal fibroblasts
PKC: protein kinase C
PMA: phorbol myristate acetate
PFA: paraformaldehyde
qPCR: quantitative polymerase chain reaction
WGA: wheat germ agglutinin

## References

[1] Eckert C, Hammesfahr B, Kollmar M. A holistic phylogeny of the coronin gene family reveals an ancient origin of the tandem-coronin, defines a new subfamily, and predicts protein function. BMC Evol Biol. 2011;11:268. doi: 10.1186/1471-2148-11-268 21943019 PMC3203266

[2] Pieters J, Müller P, Jayachandran R. On guard: coronin proteins in innate and adaptive immunity. Nat Rev Immunol. 2013;13(7):510–8. doi: 10.1038/nri3465 23765056

[3] Roadcap DW, Clemen CS, Bear JE. The role of mammalian coronins in development and disease. Subcell Biochem. 2008;48:124–35. doi: 10.1007/978-0-387-09595-0_12 18925377

[4] de Hostos EL, Bradtke B, Lottspeich F, Guggenheim R, Gerisch G. Coronin, an actin binding protein of Dictyostelium discoideum localized to cell surface projections, has sequence similarities to G protein beta subunits. EMBO J. 1991;10(13):4097–104. doi: 10.1002/j.1460-2075.1991.tb04986.x 1661669 PMC453159

[5] Gatfield J, Albrecht I, Zanolari B, Steinmetz MO, Pieters J. Association of the leukocyte plasma membrane with the actin cytoskeleton through coiled coil-mediated trimeric coronin 1 molecules. Mol Biol Cell. 2005;16(6):2786–98. doi: 10.1091/mbc.e05-01-0042 15800061 PMC1142424

[6] Appleton BA, Wu P, Wiesmann C. The crystal structure of murine coronin-1: a regulator of actin cytoskeletal dynamics in lymphocytes. Structure. 2006;14(1):87–96. doi: 10.1016/j.str.2005.09.013 16407068

[7] Nakamura T, Takeuchi K, Muraoka S, Takezoe H, Takahashi N, Mori N. A neurally enriched coronin-like protein, ClipinC, is a novel candidate for an actin cytoskeleton-cortical membrane-linking protein. J Biol Chem. 1999;274(19):13322–7. doi: 10.1074/jbc.274.19.13322 10224093

[8] Gatfield J, Pieters J. Essential role for cholesterol in entry of mycobacteria into macrophages. Science. 2000;288(5471):1647–50. doi: 10.1126/science.288.5471.1647 10834844

[9] Olshina MA, Angrisano F, Marapana DS, Riglar DT, Bane K, Wong W, et al. Plasmodium falciparum coronin organizes arrays of parallel actin filaments potentially guiding directional motility in invasive malaria parasites. Malar J. 2015;14:280. doi: 10.1186/s12936-015-0801-5 26187846 PMC4506582

[10] Riley DRJ, Khalil JS, Naseem KM, Rivero F. Biochemical and immunocytochemical characterization of coronins in platelets. Platelets. 2020;31(7):913–24. doi: 10.1080/09537104.2019.1696457 31801396 PMC7497283

[11] Chan KT, Creed SJ, Bear JE. Unraveling the enigma: progress towards understanding the coronin family of actin regulators. Trends Cell Biol. 2011;21(8):481–8. doi: 10.1016/j.tcb.2011.04.004 21632254 PMC3163407

[12] Goode BL, Eskin J, Shekhar S. Mechanisms of actin disassembly and turnover. J Cell Biol. 2023;222(12):e202309021. doi: 10.1083/jcb.202309021 37948068 PMC10638096

[13] Ndinyanka Fabrice T, Bianda C, Zhang H, Jayachandran R, Ruer-Laventie J, Mori M, et al. An evolutionarily conserved coronin-dependent pathway defines cell population size. Sci Signal. 2022;15(759):eabo5363. doi: 10.1126/scisignal.abo5363 36346836

[14] Ford ML. Coronin-1, king of alloimmunity. Immunity. 2019;50(1):3–5. doi: 10.1016/j.immuni.2018.12.030 30650379

[15] Ndinyanka Fabrice T, Buczak K, Schmidt A, Pieters J. T cell population size control by coronin 1 uncovered: from a spot identified by two-dimensional gel electrophoresis to quantitative proteomics. Expert Rev Proteomics. 2025;22(1):35–44. doi: 10.1080/14789450.2025.2450812 39849824

[16] Fechheimer M, Zigmond SH. Changes in cytoskeletal proteins of polymorphonuclear leukocytes induced by chemotactic peptides. Cell Motil. 1983;3(4):349–61. doi: 10.1002/cm.970030406 6640631

[17] de Hostos EL, Rehfuess C, Bradtke B, Waddell DR, Albrecht R, Murphy J, et al. Dictyostelium mutants lacking the cytoskeletal protein coronin are defective in cytokinesis and cell motility. J Cell Biol. 1993;120(1):163–73. doi: 10.1083/jcb.120.1.163 8380174 PMC2119478

[18] Vinet AF, Fiedler T, Studer V, Froquet R, Dardel A, Cosson P, et al. Initiation of multicellular differentiation in Dictyostelium discoideum is regulated by coronin A. Mol Biol Cell. 2014;25(5):688–701. doi: 10.1091/mbc.E13-04-0219 24403600 PMC3937094

[19] Fabrice TN, Fiedler T, Studer V, Vinet A, Brogna F, Schmidt A, et al. Interactome and F-Actin Interaction Analysis of Dictyostelium discoideum Coronin A. Int J Mol Sci. 2020;21(4):1469. doi: 10.3390/ijms21041469 32098122 PMC7073074

[20] Heil-Chapdelaine RA, Tran NK, Cooper JA. The role of *Saccharomyces cerevisiae* coronin in the actin and microtubule cytoskeletons. Curr Biol. 1998;8(23):1281–4. doi: 10.1016/s0960-9822(07)00539-8 9822583

[21] Lin M-C, Galletta BJ, Sept D, Cooper JA. Overlapping and distinct functions for cofilin, coronin and Aip1 in actin dynamics in vivo. J Cell Sci. 2010;123(Pt 8):1329–42. doi: 10.1242/jcs.065698 20332110 PMC2848116

[22] Mueller P, Massner J, Jayachandran R, Combaluzier B, Albrecht I, Gatfield J, et al. Regulation of T cell survival through coronin-1-mediated generation of inositol-1,4,5-trisphosphate and calcium mobilization after T cell receptor triggering. Nat Immunol. 2008;9(4):424–31. doi: 10.1038/ni1570 18345003

[23] Haraldsson MK, Louis-Dit-Sully CA, Lawson BR, Sternik G, Santiago-Raber M-L, Gascoigne NRJ, et al. The lupus-related Lmb3 locus contains a disease-suppressing Coronin-1A gene mutation. Immunity. 2008;28(1):40–51. doi: 10.1016/j.immuni.2007.11.023 18199416 PMC2274909

[24] Jayachandran R, Sundaramurthy V, Combaluzier B, Mueller P, Korf H, Huygen K, et al. Survival of mycobacteria in macrophages is mediated by coronin 1-dependent activation of calcineurin. Cell. 2007;130(1):37–50. doi: 10.1016/j.cell.2007.04.043 17632055

[25] Jayachandran R, Gatfield J, Massner J, Albrecht I, Zanolari B, Pieters J. RNA interference in J774 macrophages reveals a role for coronin 1 in mycobacterial trafficking but not in actin-dependent processes. Mol Biol Cell. 2008;19(3):1241–51. doi: 10.1091/mbc.e07-07-0640 18162581 PMC2262969

[26] Mueller P, Liu X, Pieters J. Migration and homeostasis of naive T cells depends on coronin 1-mediated prosurvival signals and not on coronin 1-dependent filamentous actin modulation. J Immunol. 2011;186(7):4039–50. doi: 10.4049/jimmunol.1003352 21339362

[27] Jayachandran R, Liu X, Bosedasgupta S, Müller P, Zhang C-L, Moshous D, et al. Coronin 1 regulates cognition and behavior through modulation of cAMP/protein kinase A signaling. PLoS Biol. 2014;12(3):e1001820. doi: 10.1371/journal.pbio.1001820 24667537 PMC3965382

[28] Suo D, Park J, Harrington AW, Zweifel LS, Mihalas S, Deppmann CD. Coronin-1 is a neurotrophin endosomal effector that is required for developmental competition for survival. Nat Neurosci. 2014;17(1):36–45. doi: 10.1038/nn.3593 24270184 PMC3962792

[29] Jayachandran R, Gumienny A, Bolinger B, Ruehl S, Lang MJ, Fucile G, et al. Disruption of coronin 1 signaling in T cells promotes allograft tolerance while maintaining anti-pathogen immunity. Immunity. 2019;50(1):152-165.e8. doi: 10.1016/j.immuni.2018.12.011 30611611

[30] Mori M, Ruer-Laventie J, Duchemin W, Demougin P, Ndinyanka Fabrice T, Wymann MP, et al. Suppression of caspase 8 activity by a coronin 1-PI3Kδ pathway promotes T cell survival independently of TCR and IL-7 signaling. Sci Signal. 2021;14(714):eabj0057. doi: 10.1126/scisignal.abj0057 34932374

[31] Uetrecht AC, Bear JE. Coronins: the return of the crown. Trends Cell Biol. 2006;16(8):421–6. doi: 10.1016/j.tcb.2006.06.002 16806932

[32] Gandhi M, Goode BL. Coronin: the double-edged sword of actin dynamics. Subcell Biochem. 2008;48:72–87. doi: 10.1007/978-0-387-09595-0_7 18925372

[33] Föger N, Rangell L, Danilenko DM, Chan AC. Requirement for coronin 1 in T lymphocyte trafficking and cellular homeostasis. Science. 2006;313(5788):839–42. doi: 10.1126/science.1130563 16902139

[34] Suo D, Park J, Young S, Makita T, Deppmann CD. Coronin-1 and calcium signaling governs sympathetic final target innervation. J Neurosci. 2015;35(9):3893–902. doi: 10.1523/JNEUROSCI.4402-14.2015 25740518 PMC4348186

[35] Tsien RY. The green fluorescent protein. Annu Rev Biochem. 1998;67:509–44. doi: 10.1146/annurev.biochem.67.1.509 9759496

[36] Cubitt AB, Woollenweber LA, Heim R. Understanding structure-function relationships in the Aequorea victoria green fluorescent protein. Methods Cell Biol. 1999;58:19–30. doi: 10.1016/s0091-679x(08)61946-9 9891372

[37] Hopp TP, Prickett KS, Price VL, Libby RT, March CJ, Pat Cerretti D, et al. A short polypeptide marker sequence useful for recombinant protein identification and purification. Nat Biotechnol. 1988;6(10):1204–10. doi: 10.1038/nbt1088-1204

[38] Margolin W. The price of tags in protein localization studies. J Bacteriol. 2012;194(23):6369–71. doi: 10.1128/JB.01640-12 22961859 PMC3497479

[39] Stadler C, Rexhepaj E, Singan VR, Murphy RF, Pepperkok R, Uhlén M, et al. Immunofluorescence and fluorescent-protein tagging show high correlation for protein localization in mammalian cells. Nat Methods. 2013;10(4):315–23. doi: 10.1038/nmeth.2377 23435261

[40] Weill U, Krieger G, Avihou Z, Milo R, Schuldiner M, Davidi D. Assessment of GFP tag position on protein localization and growth fitness in yeast. J Mol Biol. 2019;431(3):636–41. doi: 10.1016/j.jmb.2018.12.004 30550779 PMC7611381

[41] Saper CB. An open letter to our readers on the use of antibodies. J Comp Neurol. 2005;493(4):477–8. doi: 10.1002/cne.20839 16304632

[42] Saper CB. A guide to the perplexed on the specificity of antibodies. J Histochem Cytochem. 2009;57(1):1–5. doi: 10.1369/jhc.2008.952770 18854594 PMC2605712

[43] Scherer WF, Syverton JT, Gey GO. Studies on the propagation in vitro of poliomyelitis viruses. IV. Viral multiplication in a stable strain of human malignant epithelial cells (strain HeLa) derived from an epidermoid carcinoma of the cervix. J Exp Med. 1953;97(5):695–710. doi: 10.1084/jem.97.5.695 13052828 PMC2136303

[44] Estes JE, Selden LA, Gershman LC. Mechanism of action of phalloidin on the polymerization of muscle actin. Biochemistry. 1981;20(4):708–12. doi: 10.1021/bi00507a006 6452160

[45] Cooper JA. Effects of cytochalasin and phalloidin on actin. J Cell Biol. 1987;105(4):1473–8. doi: 10.1083/jcb.105.4.1473 3312229 PMC2114638

[46] King ZT, Butler MT, Hockenberry MA, Subramanian BC, Siesser PF, Graham DM, et al. Coro1B and Coro1C regulate lamellipodia dynamics and cell motility by tuning branched actin turnover. J Cell Biol. 2022;221(8):e202111126. doi: 10.1083/jcb.202111126 35657370 PMC9170525

[47] Okumura M, Kung C, Wong S, Rodgers M, Thomas ML. Definition of family of coronin-related proteins conserved between humans and mice: close genetic linkage between coronin-2 and CD45-associated protein. DNA Cell Biol. 1998;17(9):779–87. doi: 10.1089/dna.1998.17.779 9778037

[48] Ferrari G, Langen H, Naito M, Pieters J. A coat protein on phagosomes involved in the intracellular survival of mycobacteria. Cell. 1999;97(4):435–47. doi: 10.1016/s0092-8674(00)80754-0 10338208

[49] Dunn KW, Kamocka MM, McDonald JH. A practical guide to evaluating colocalization in biological microscopy. Am J Physiol Cell Physiol. 2011;300(4):C723-42. doi: 10.1152/ajpcell.00462.2010 21209361 PMC3074624

[50] Cordelières FP, Bolte S. Experimenters’ guide to colocalization studies: finding a way through indicators and quantifiers, in practice. Methods Cell Biol. 2014;123:395–408. doi: 10.1016/B978-0-12-420138-5.00021-5 24974039

[51] Schnell U, Dijk F, Sjollema KA, Giepmans BNG. Immunolabeling artifacts and the need for live-cell imaging. Nat Methods. 2012;9(2):152–8. doi: 10.1038/nmeth.1855 22290187

[52] Melan MA, Sluder G. Redistribution and differential extraction of soluble proteins in permeabilized cultured cells. Implications for immunofluorescence microscopy. J Cell Sci. 1992;101 (Pt 4):731–43. doi: 10.1242/jcs.101.4.731 1527176

[53] Chazotte B. Labeling membrane glycoproteins or glycolipids with fluorescent wheat germ agglutinin. Cold Spring Harb Protoc. 2011;2011(5):pdb.prot5623. doi: 10.1101/pdb.prot5623 21536757

[54] Tsujita K, Itoh T, Kondo A, Oyama M, Kozuka-Hata H, Irino Y, et al. Proteome of acidic phospholipid-binding proteins: spatial and temporal regulation of Coronin 1A by phosphoinositides. J Biol Chem. 2010;285(9):6781–9. doi: 10.1074/jbc.M109.057018 20032464 PMC2825472

[55] Chan KT, Roadcap DW, Holoweckyj N, Bear JE. Coronin 1C harbours a second actin-binding site that confers co-operative binding to F-actin. Biochem J. 2012;444(1):89–96. doi: 10.1042/BJ20120209 22364218 PMC3339874

[56] Liu HS, Jan MS, Chou CK, Chen PH, Ke NJ. Is green fluorescent protein toxic to the living cells?. Biochem Biophys Res Commun. 1999;260(3):712–7. doi: 10.1006/bbrc.1999.0954 10403831

[57] Agbulut O, Coirault C, Niederländer N, Huet A, Vicart P, Hagège A, et al. GFP expression in muscle cells impairs actin-myosin interactions: implications for cell therapy. Nat Methods. 2006;3(5):331. doi: 10.1038/nmeth0506-331 16628201

[58] Agbulut O, Huet A, Niederländer N, Puceat M, Menasché P, Coirault C. Green fluorescent protein impairs actin-myosin interactions by binding to the actin-binding site of myosin. J Biol Chem. 2007;282(14):10465–71. doi: 10.1074/jbc.M610418200 17289667

[59] Baens M, Noels H, Broeckx V, Hagens S, Fevery S, Billiau AD, et al. The dark side of EGFP: defective polyubiquitination. Plos One. 2006;1(1):e54 doi: 10.1371/journal.pone.0000054 PMC1762387 17183684

[60] Montecinos-Franjola F, Bauer BL, Mears JA, Ramachandran R. GFP fluorescence tagging alters dynamin-related protein 1 oligomerization dynamics and creates disassembly-refractory puncta to mediate mitochondrial fission. Sci Rep. 2020;10(1):14777. Epub 2020/09/10. doi: 10.1038/s41598-020-71655-x 32901052 PMC7479153

[61] Swulius MT, Jensen GJ. The helical MreB cytoskeleton in *Escherichia coli* MC1000/pLE7 is an artifact of the N-Terminal yellow fluorescent protein tag. J Bacteriol. 2012;194(23):6382–6. doi: 10.1128/JB.00505-12 22904287 PMC3497537

[62] Simpson JC, Joggerst B, Laketa V, Verissimo F, Cetin C, Erfle H, et al. Genome-wide RNAi screening identifies human proteins with a regulatory function in the early secretory pathway. Nat Cell Biol. 2012;14(7):764–74. doi: 10.1038/ncb2510 22660414

[63] Kimple ME, Sondek J. Overview of affinity tags for protein purification. Curr Protoc Protein Sci. 2004;Chapter 9:9.9.1-9.9.19. doi: 10.1002/0471140864.ps0909s36 18429272

[64] Shiow LR, Roadcap DW, Paris K, Watson SR, Grigorova IL, Lebet T, et al. The actin regulator coronin 1A is mutant in a thymic egress-deficient mouse strain and in a patient with severe combined immunodeficiency. Nat Immunol. 2008;9(11):1307–15. doi: 10.1038/ni.1662 18836449 PMC2672406

[65] Mugnier B, Nal B, Verthuy C, Boyer C, Lam D, Chasson L, et al. Coronin-1A links cytoskeleton dynamics to TCR alpha beta-induced cell signaling. PLoS One. 2008;3(10):e3467. doi: 10.1371/journal.pone.0003467 18941544 PMC2568942

[66] Lang MJ, Mori M, Ruer-Laventie J, Pieters J. A coronin 1-Dependent Decision Switch In Juvenile Mice Determines The Population Of The Peripheral Naive T cell compartment. J Immunol. 2017;199(7):2421–31. doi: 10.4049/jimmunol.1700438 28821585

[67] Coué M, Brenner SL, Spector I, Korn ED. Inhibition of actin polymerization by latrunculin A. FEBS Lett. 1987;213(2):316–8. doi: 10.1016/0014-5793(87)81513-2 3556584

[68] Bosedasgupta S, Pieters J. Inflammatory stimuli reprogram macrophage phagocytosis to macropinocytosis for the rapid elimination of pathogens. PLoS Pathog. 2014;10(1):e1003879. doi: 10.1371/journal.ppat.1003879 24497827 PMC3907376

[69] BoseDasgupta S, Pieters J. Coronin 1 trimerization is essential to protect pathogenic mycobacteria within macrophages from lysosomal delivery. FEBS Lett. 2014;588(21):3898–905. doi: 10.1016/j.febslet.2014.08.036 25217836

[70] Niedel JE, Kuhn LJ, Vandenbark GR. Phorbol diester receptor co-purifies with protein kinase-C. P Natl Acad Sci-Biol. 1983;80(1):36–40. doi: 10.1073/pnas.80.1.36 PMC393304 6296873

[71] Zick Y, Sagi-Eisenberg R. A combination of H2O2 and vanadate concomitantly stimulates protein tyrosine phosphorylation and polyphosphoinositide breakdown in different cell lines. Biochemistry. 1990;29(44):10240–5. doi: 10.1021/bi00496a013 2176864

[72] Herbert JM, Augereau JM, Gleye J, Maffrand JP. Chelerythrine is a potent and specific inhibitor of protein kinase C. Biochem Biophys Res Commun. 1990;172(3):993–9. doi: 10.1016/0006-291x(90)91544-3 2244923

[73] Mueller P, Quintana A, Griesemer D, Hoth M, Pieters J. Disruption of the cortical actin cytoskeleton does not affect store operated Ca2+ channels in human T-cells. FEBS Lett. 2007;581(18):3557–62. doi: 10.1016/j.febslet.2007.06.068 17624329

[74] Bubb MR, Senderowicz AM, Sausville EA, Duncan KL, Korn ED. Jasplakinolide, a cytotoxic natural product, induces actin polymerization and competitively inhibits the binding of phalloidin to F-actin. J Biol Chem. 1994;269(21):14869–71. doi: 10.1016/s0021-9258(17)36545-6 8195116

[75] Schafer DA, Gill SR, Cooper JA, Heuser JE, Schroer TA. Ultrastructural analysis of the dynactin complex: an actin-related protein is a component of a filament that resembles F-actin. J Cell Biol. 1994;126(2):403–12. doi: 10.1083/jcb.126.2.403 7518465 PMC2200042

[76] Machesky LM, Atkinson SJ, Ampe C, Vandekerckhove J, Pollard TD. Purification of a cortical complex containing two unconventional actins from Acanthamoeba by affinity chromatography on profilin-agarose. J Cell Biol. 1994;127(1):107–15. doi: 10.1083/jcb.127.1.107 7929556 PMC2120189

[77] von Toerne C, Sieg C, Kaufmann U, Diedrichs-Möhring M, Nelson PJ, Wildner G. Effector T cells driving monophasic vs. relapsing/remitting experimental autoimmune uveitis show unique pathway signatures. Mol Immunol. 2010;48(1–3):272–80. doi: 10.1016/j.molimm.2010.07.017 20727594

[78] Liu S-L, Needham KM, May JR, Nolen BJ. Mechanism of a concentration-dependent switch between activation and inhibition of Arp2/3 complex by coronin. J Biol Chem. 2011;286(19):17039–46. doi: 10.1074/jbc.M111.219964 21454476 PMC3089548

[79] Goode BL, Wong JJ, Butty AC, Peter M, McCormack AL, Yates JR, et al. Coronin promotes the rapid assembly and cross-linking of actin filaments and may link the actin and microtubule cytoskeletons in yeast. J Cell Biol. 1999;144(1):83–98. doi: 10.1083/jcb.144.1.83 9885246 PMC2148128

[80] Gandhi M, Achard V, Blanchoin L, Goode BL. Coronin switches roles in actin disassembly depending on the nucleotide state of actin. Mol Cell. 2009;34(3):364–74. doi: 10.1016/j.molcel.2009.02.029 19450534 PMC2702861

[81] Ge P, Durer ZA, Kudryashov D, Zhou ZH, Reisler E. Cryo-EM reveals different coronin binding modes for ADP- and ADP-BeFx actin filaments. Nat Struct Mol Biol. 2014;21(12):1075–81. doi: 10.1038/nsmb.2907 25362487 PMC4388421

[82] Liu X, BoseDasgupta S, Jayachandran R, Studer V, Rühl S, Stiess M, et al. Activation of the cAMP/protein kinase A signalling pathway by coronin 1 is regulated by cyclin-dependent kinase 5 activity. FEBS Lett. 2016;590(2):279–87. doi: 10.1002/1873-3468.12046 26823173

[83] Saha S, Hazra A, Ghatak D, Singh AV, Roy S, BoseDasgupta S. A bumpy ride of mycobacterial phagosome maturation: roleplay of coronin1 through cofilin1 and cAMP. Front Immunol. 2021;12:687044. doi: 10.3389/fimmu.2021.687044 34630380 PMC8495260

[84] Klarenbeek J, Goedhart J, van Batenburg A, Groenewald D, Jalink K. Fourth-generation epac-based FRET sensors for cAMP feature exceptional brightness, photostability and dynamic range: characterization of dedicated sensors for FLIM, for ratiometry and with high affinity. PLoS One. 2015;10(4):e0122513. doi: 10.1371/journal.pone.0122513 25875503 PMC4397040

[85] Insel PA, Ostrom RS. Forskolin as a tool for examining adenylyl cyclase expression, regulation, and G protein signaling. Cell Mol Neurobiol. 2003;23(3):305–14. 12825829 10.1023/A:1023684503883 PMC11530207

[86] Oosterheert W, Boiero Sanders M, Hofnagel O, Bieling P, Raunser S. Choreography of rapid actin filament disassembly by coronin, cofilin, and AIP1. Cell. 2025;188(24):6845-6860.e27. doi: 10.1016/j.cell.2025.09.016 41075793

[87] Swaminathan K, Müller-Taubenberger A, Faix J, Rivero F, Noegel AA. A Cdc42- and Rac-interactive binding (CRIB) domain mediates functions of coronin. Proc Natl Acad Sci U S A. 2014;111(1):E25-33. doi: 10.1073/pnas.1315368111 24347642 PMC3890859

[88] Kimura T, Kaneko Y, Yamada S, Ishihara H, Senda T, Iwamatsu A, et al. The GDP-dependent Rab27a effector coronin 3 controls endocytosis of secretory membrane in insulin-secreting cell lines. J Cell Sci. 2008;121(Pt 18):3092–8. doi: 10.1242/jcs.030544 18768935

[89] Shaverskyi A, Hegermann J, Brand K, Lee K-H, Föger N. Coronin 1a-mediated F-actin disassembly controls effector function in murine neutrophils. Redox Biol. 2025;82:103618. doi: 10.1016/j.redox.2025.103618 40158258 PMC11997354

[90] Kelley CF, Litschel T, Schumacher S, Dedden D, Schwille P, Mizuno N. Phosphoinositides regulate force-independent interactions between talin, vinculin, and actin. Elife. 2020;9:e56110. doi: 10.7554/eLife.56110 32657269 PMC7384861

[91] Johnson RP, Craig SW. F-actin binding site masked by the intramolecular association of vinculin head and tail domains. Nature. 1995;373(6511):261–4. doi: 10.1038/373261a0 7816144

[92] Craig SW, Johnson RP. Assembly of focal adhesions: progress, paradigms, and portents. Curr Opin Cell Biol. 1996;8(1):74–85. doi: 10.1016/s0955-0674(96)80051-2 8791409

[93] Pareek TK, Lam E, Zheng X, Askew D, Kulkarni AB, Chance MR, et al. Cyclin-dependent kinase 5 activity is required for T cell activation and induction of experimental autoimmune encephalomyelitis. J Exp Med. 2010;207(11):2507–19. doi: 10.1084/jem.20100876 20937706 PMC2964575

[94] Itoh S, Suzuki K, Nishihata J, Iwasa M, Oku T, Nakajin S, et al. The role of protein kinase C in the transient association of p57, a coronin family actin-binding protein, with phagosomes. Biol Pharm Bull. 2002;25(7):837–44. doi: 10.1248/bpb.25.837 12132654

[95] Spoerl Z, Stumpf M, Noegel AA, Hasse A. Oligomerization, F-actin interaction, and membrane association of the ubiquitous mammalian coronin 3 are mediated by its carboxyl terminus. J Biol Chem. 2002;277(50):48858–67. doi: 10.1074/jbc.M205136200 12377779

[96] Brogna F. Identification of coronin A interacting partners in dictyostelium discoideum. Basel: Biozentrum, Universität Basel; 2015. Available from: https://basel.swisscovery.org/permalink/41SLSP_UBS/tocbsk/alma9973116066505504

[97] Fiedler T, Fabrice TN, Studer V, Vinet A, Faltova L, Kammerer RA, et al. Homodimerization of coronin A through the C-terminal coiled-coil domain is essential for multicellular differentiation of Dictyostelium discoideum. FEBS Lett. 2020. doi: 10.1002/1873-3468.13787 32298460

[98] Machesky LM, Reeves E, Wientjes F, Mattheyse FJ, Grogan A, Totty NF, et al. Mammalian actin-related protein 2/3 complex localizes to regions of lamellipodial protrusion and is composed of evolutionarily conserved proteins. Biochem J. 1997;328 (Pt 1) (Pt 1):105–12. doi: 10.1042/bj3280105 9359840 PMC1218893

[99] Combaluzier B, Pieters J. Chemotaxis and phagocytosis in neutrophils is independent of coronin 1. J Immunol. 2009;182(5):2745–52. doi: 10.4049/jimmunol.0801812 19234169

[100] Tang WJ, Gilman AG. Adenylyl cyclases. Cell. 1992;70(6):869–72. doi: 10.1016/0092-8674(92)90236-6 1525824

[101] Gilman AG. G proteins: transducers of receptor-generated signals. Annu Rev Biochem. 1987;56:615–49. doi: 10.1146/annurev.bi.56.070187.003151 3113327

[102] Casey PJ, Graziano MP, Freissmuth M, Gilman AG. Role of G proteins in transmembrane signaling. Cold Spring Harb Symp Quant Biol. 1988;53 Pt 1:203–8. doi: 10.1101/sqb.1988.053.01.026 3151167

[103] Means AR, Tash JS, Chafouleas JG, Lagace L, Guerriero V. Regulation of the cytoskeleton by Ca2+-calmodulin and cAMP. Ann N Y Acad Sci. 1982;383:69–84. doi: 10.1111/j.1749-6632.1982.tb23162.x 6283996

[104] Kaminski S, Hermann-Kleiter N, Meisel M, Thuille N, Cronin S, Hara H, et al. Coronin 1A is an essential regulator of the TGFβ receptor/SMAD3 signaling pathway in Th17 CD4(+) T cells. J Autoimmun. 2011;37(3):198–208. doi: 10.1016/j.jaut.2011.05.018 21700422

[105] Jinek M, Chylinski K, Fonfara I, Hauer M, Doudna JA, Charpentier E. A programmable dual-RNA-guided DNA endonuclease in adaptive bacterial immunity. Science. 2012;337(6096):816–21. doi: 10.1126/science.1225829 22745249 PMC6286148

[106] Modzelewski AJ, Chen S, Willis BJ, Lloyd KCK, Wood JA, He L. Efficient mouse genome engineering by CRISPR-EZ technology. Nat Protoc. 2018;13(6):1253–74. doi: 10.1038/nprot.2018.012 29748649 PMC6296855

[107] Haueter S, Kawasumi M, Asner I, Brykczynska U, Cinelli P, Moisyadi S, et al. Genetic vasectomy-overexpression of Prm1-EGFP fusion protein in elongating spermatids causes dominant male sterility in mice. Genesis. 2010;48(3):151–60. doi: 10.1002/dvg.20598 20095053 PMC3432410

[108] Johnson JP, Demmer-Dieckmann M, Meo T, Hadam MR, Riethmüller G. Surface antigens of human melanoma cells defined by monoclonal antibodies. I. Biochemical characterization of two antigens found on cell lines and fresh tumors of diverse tissue origin. Eur J Immunol. 1981;11(10):825–31. doi: 10.1002/eji.1830111015 6895502

[109] Pieters J, Horstmann H, Bakke O, Griffiths G, Lipp J. Intracellular transport and localization of major histocompatibility complex class II molecules and associated invariant chain. J Cell Biol. 1991;115(5):1213–23. doi: 10.1083/jcb.115.5.1213 1955469 PMC2289241

[110] Tulp A, Verwoerd D, Dobberstein B, Ploegh HL, Pieters J. Isolation and characterization of the intracellular MHC class II compartment. Nature. 1994;369(6476):120–6. doi: 10.1038/369120a0 8177317

[111] Ran FA, Hsu PD, Wright J, Agarwala V, Scott DA, Zhang F. Genome engineering using the CRISPR-Cas9 system. Nat Protoc. 2013;8(11):2281–308. doi: 10.1038/nprot.2013.143 24157548 PMC3969860

[112] Ruer-Laventie J, Jayachandran R, Schmaler M, Rossi SW, Pieters J. Skin transplantation and lymphoid organ analysis in mice. Bio-Protocol. 2020;10(4). doi: 10.21769/BioProtoc.3531 PMC7842299 33654755

[113] Waterman-Storer CM. Microtubule/organelle motility assays. Curr Protocols Cell Biol. 1998;(1):13.1.1–13.1.21. doi: 10.1002/0471143030.cb1301s00 18228320

[114] Kohler D, Staniak M, Tsai T-H, Huang T, Shulman N, Bernhardt OM, et al. MSstats version 4.0: statistical analyses of quantitative mass spectrometry-based proteomic experiments with chromatography-based quantification at scale. J Proteome Res. 2023;22(5):1466–82. doi: 10.1021/acs.jproteome.2c00834 37018319 PMC10629259

[115] Bolger AM, Lohse M, Usadel B. Trimmomatic: a flexible trimmer for Illumina sequence data. Bioinformatics. 2014;30(15):2114–20. doi: 10.1093/bioinformatics/btu170 24695404 PMC4103590

[116] Dobin A, Davis CA, Schlesinger F, Drenkow J, Zaleski C, Jha S, et al. STAR: ultrafast universal RNA-seq aligner. Bioinformatics. 2013;29(1):15–21. doi: 10.1093/bioinformatics/bts635 23104886 PMC3530905

[117] Love MI, Huber W, Anders S. Moderated estimation of fold change and dispersion for RNA-seq data with DESeq2. Genome Biol. 2014;15(12):550. doi: 10.1186/s13059-014-0550-8 25516281 PMC4302049

[118] Stephens M. False discovery rates: a new deal. Biostatistics. 2017;18(2):275–94. doi: 10.1093/biostatistics/kxw041 27756721 PMC5379932

[119] Wóycicki RK. RNA-Seq Power Calculator. Rafalwoycicki/rafalwoycicki.github.io: V.0.0.2.0: Zenedo; 2025.

