# Supplementary material for: Reassessment of the roles of coronin proteins as actin effectors and in signaling
Source: PLoS Biol. 2026 Aug 18;24(8):e3003904. doi: 10.1371/journal.pbio.3003904 (PMC13485075; doi:10.1371/journal.pbio.3003904)
Supplement: Supplementary file 1 [file pbio.3003904.s011.pdf]

S2 Table: Anti-coronin 2 and 3 antibodies tested in HeLa wild type and coronin 2 or 3-deficient cells

Anti-coronin 2 antibodies tested in HeLa wild type and coronin 2-deficient cells

Antibody	Species	Information available:	Results	Wild type cells	Coronin 2-deficient cells	Dilutions tested
**HPA040113**	Rabbit, polyclonal, affinity purified; Atlas prestige project	https://www.sigmaaldrich.com/CH/fr/product/sigma/hpa040113	Unspecifically stains Cor2KO	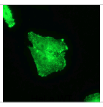	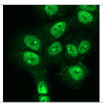	1:500 now, 1:1000 and 1:2000 previously tested
**SC-271445,**	Mouse monoclonal (IgG2b κ), clone G-8	https://www.scbt.com/p/coronin-1b-antibody-g-8	Specific and being used	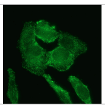	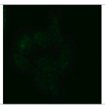	1:500 being used, 1:100, 1:200 and 1:1000 have been screened in the past
**HPA070456**	Rabbit, polyclonal, affinity purified; Atlas prestige project	https://www.sigmaaldrich.com/CH/de/product/sigma/hpa070456	Largely specific, various levels of unspecific stain in the Golgi area	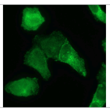	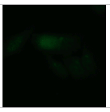	1:500 now, 1:1000 and 1:2000 previously tested
**PA5-64443**	Rabbit, polyclonal	https://www.thermofisher.com/antibody/product/Coronin-1B-Antibody-Polyclonal/PA5-64443	Largely specific, various levels of unspecific stain in the Golgi area	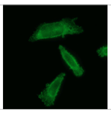	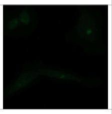	1:500 and 1:1000

Anti-coronin 3 antibodies tested in HeLa wild type and coronin 3-deficient cells

Antibody	Species	Information available:	Results	Wild type cells	Coronin 3-deficient cells	Dilutions tested
**14749-1-AP**	Rabbit, IgG, Polyclonal	https://www.ptglab.com/products/CORO1C-Antibody-14749-1-AP.htm	Unspecific	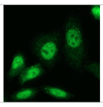	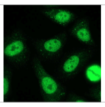	1:200 and 1:500
**AB15719**	Goat, polyclonal	https://www.abcam.com/en-us/products/primary-antibodies/coronin-1c-antibody-ab15719	Unspecific	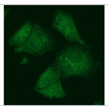	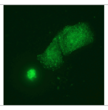	1:1000
**AB283693**	Rabbit, monoclonal	https://www.abcam.com/en-us/products/primary-antibodies/coronin-1c-antibody-epr25365-24-ab283693	Unspecific	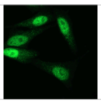	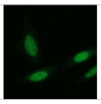	1:500 and 1:1000
**H00023603-M02**	Mouse, monoclonal, IgG2a Kappa	https://www.abnova.com/en-global/product/detail/H00023603-M02	Unspecific, signal only slightly stronger than in secondary antibody controls in both WT and KO cells	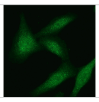	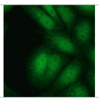	1:500 and 1:1000
**SC-376919 (D9)**	Mouse, monoclonal, IgM Kappa	https://www.scbt.com/p/coronin-1c-antibody-d-9	Unspecific, signal only slightly stronger than in secondary antibody controls in both WT and KO cells	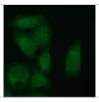	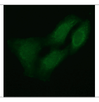	1:200, 1:500 and 1:1000
**K6-444**	Mouse, monoclonal	https://pubmed.ncbi.nlm.nih.gov/12377779/	Unspecific, signal only slightly stronger than in secondary antibody controls in both WT and KO cells	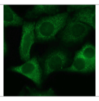	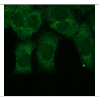	1:50 and 1:100, higher dilutions tested previously by others
**Pur50**	Rabbit, polyclonal	Home made anti-peptide antiserum	Unspecific	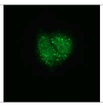	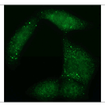	1:100, 1:500, 1:1000
**Pur51**	Rabbit, polyclonal	Home made anti-peptide antiserum	Unspecific	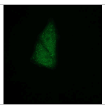	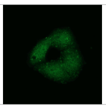	1:100, 1:500, 1:1000
**8-2.2**	Mouse, monoclonal, purified	See supplementary information	Unspecific	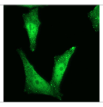	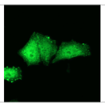	1:200 and 1:500
**SC-130448 (G-R2)**	Mouse, monoclonal, IgG2a Kappa	https://www.scbt.com/p/coronin-1c-antibody-g-r2	Unspecific, signal only slightly stronger than in secondary antibody controls in both WT and KO cells	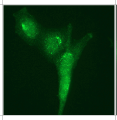	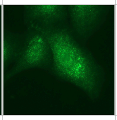	1:200 and 1:500
