# Supplementary material for: Reassessment of the roles of coronin proteins as actin effectors and in signaling
Source: PLoS Biol. 2026 Aug 18;24(8):e3003904. doi: 10.1371/journal.pbio.3003904 (PMC13485075; doi:10.1371/journal.pbio.3003904)
Supplement: Supplementary file 2 [file pbio.3003904.s013.pdf]

S4 Table: Literature referring to coronin and actin modulation

This table lists the publications in which coronin 1, 2 or 3 proteins are described to be connected to actin dynamics and/or actin-related functions.

This lists are the basis of the proteins listed in Table S3 and indicated in Figure 5.

Coronin 1

References				Specific proteins mentioned in the study
Kueh..Brieher 2008	J. Cell Biol. (2008) 182:341–353	**Actin disassembly by cofilin, coronin, and Aip1 occurs in bursts and is inhibited by barbed-end cappers**	PMID: 18663144	Actin, Cofilin, Actin-interacting protein 1 (Aip1)
Galkin..Egelman 2008	J. Mol. Biol. (2008) 376:607–613	**Coronin-1A Stabilizes F-Actin by Bridging Adjacent Actin Protomers and Stapling Opposite Strands of the Actin Filament**	PMID: 18177666	Actin, Arp2/3 complex; Cofilin;
Brieher..Mitchinson 2006	J. Cell Biol. (2006) 175:315-324	**Rapid actin monomer–insensitive depolymerization of Listeria actin comet tails by cofilin, coronin, and Aip1**	PMID: 17060499	Cofilin, Aip1
Liu..Sui 2006	Cell Research (2006) 16:106-112	**The identification of a new actin-binding region in p57**	PMID: 16467882	Actin
Mace..Orange 2014	Proc. Natl. Acad. Sci. USA (2014) 111:6708-13	**Lytic immune synapse function requires filamentous actin deconstruction by Coronin 1A**	PMID: 24760828	Actin
Foeger..Chan 2006	Science (2006) 313:839-842	**Requirement for Coronin 1 in T Lymphocyte Trafficking and Cellular Homeostasis**	PMID: 16902139	Actin, Arp2/3 complex
Shiow..Cyster 2008	Nature Immunol. (2008) 9:424-431	**The actin regulator coronin 1A is mutant in a thymic egress–deficient mouse strain and in a patient with severe combined immunodeficiency**	PMID: 18836449	Actin, Arp2/3 complex
Pick..Walzog 2017	Blood (2017) 130:847-858	**Coronin 1A, a novel player in integrin biology, controls neutrophil trafficking in innate immunity**	PMID: 28615221	β2 integral (ITGB2, CD18)
Foeger..Chan 2006	Science (2006) 313:839-842	**Requirement for Coronin 1 in T Lymphocyte Trafficking and Cellular Homeostasis**	PMID: 16902139	Actin, Arp2/3 complex
Shiow..Cyster 2008	Nature Immunol. (2008) 9:424-431	**The actin regulator coronin 1A is mutant in a thymic egress–deficient mouse strain and in a patient with severe combined immunodeficiency**	PMID: 18836449	Actin, Arp2/3 complex
Yan..Trimble 2007	J. Immunol. (2007) 178:5769-5778	**Coronin Function Is Required for Chemotaxis and Phagocytosis in Human Neutrophils1**	PMID: 17442961	Actin, Arp2/3 complex
Yan..Trimble 2005	Mol. Biol. Cell (2005) 16:3077-87	**Coronin-1 function is required for phagosome formation**	PMID: 15829569	Actin, Arp3
Terzi..Ayter 2014	Neurosci Lett (2014) 582:38-42	**Coronin 1A inhibits neurite outgrowth in PC12 cells**	PMID: 25179994	Actin
Foeger..Bulfoni Paus 2011	J. Exp. Med. (2011) 208:1777-1787	**Differential regulation of mast cell degranulation versus cytokine secretion by the actin regulatory proteins Coronin1a and Coronin1b**	PMID: 21844203	Actin

Coronin 2

References				Specific proteins mentioned in the study
Cai..Bear 2007	J. Cell Sci. (2007) 120:2796-2806	**F-actin binding is essential for coronin 1B function in vivo**	PMID: 17456547	Actin, cofilin
Jansen..Goode 2015	Nat Commun (2015) 6:7202	**Single-molecule imaging of a three-component ordered actin disassembly mechanism**	PMID: 25995115	Actin, cofilin, Aip1
Chin..Goode 2016	J Mol. Biol. (2016) 428:1604-16	**TIRF microscopy analysis of human Cof1, Cof2, and ADF effects on actin filament severing and turnover**	PMID: 26996939	Actin, cofilin (cofilin 1, cofilin 2), actin-depolymerizing factor (ADF)
Sinha..Weaver 2016	J Cell Biol (2016) 214:197-213	**Cortactin promotes exosome secretion by controlling branched actin dynamics**	PMID: 27402952	Actin, contacting, Rab27
Merino..Raunser 2018	Nat Struct Mol Biol (2018) 25:528-537	**Structural transitions of F-actin upon ATP hydrolysis at near-atomic resolution revealed by cryo-EM**	PMID: 29867215	Actin
Molinie..Gautreau 2019	Cell Res (2019) 29:432-445	**Cortical branched actin determines cell cycle progression**	PMID: 30971746	Actin, Arp2/3
Werner..Montanez 2020	Front Cell Dev Biol (2020) 8:708	**Coronin 1B Controls Endothelial Actin Dynamics at Cell–Cell Junctions and Is Required for Endothelial Network Assembly**	PMID: 32850828	Arp2/3, Integrin-linked kinase (ILK)
Hachimi..Belmonte 2021	Curr Biol (2021) 31:696-706.e9	**Smoothelin-like 2 Inhibits Coronin-1B to Stabilize the Apical Actin Cortex during Epithelial Morphogenesis**	PMID: 33275893	Actin, Arp2/3, smoothelin-like-2 (SMTNL2)
King..Bear 2022	J Cell Biol (2022) 221:e202111126	**Coro1B and Coro1C regulate lamellipodia dynamics and cell motility by tuning branched actin turnover**	PMID: 35657370	Actin, Arp2/3, cofilin
Cai..Bear 2005	J. Biol. Chem. (2005) 280:31913–31923	**Phosphorylation of Coronin 1B by Protein Kinase C Regulates Interaction with Arp2/3 and Cell Motility**	PMID: 16027158	Actin, Arp2/3
Cai..Bear 2007	Cell (2007) 128:915–929	**Coronin 1B Coordinates Arp2/3 Complex and Cofilin Activities at the Leading Edge**	PMID: 17350576	Actin, Arp2/3, cofilin, slingshot (SSH1L) phosphatase
Cai..Bear 2008	Cell (2008) 34:828–842	**Coronin 1B antagonizes cortactin and remodels Arp2/3-containing actin branches in lamellipodia**	PMID: 18775315	Actin, Arp2/3
Howell..Fotedar 2015	J Cell Biol (2015) 208:961-74	**WISp39 binds phosphorylated Coronin 1B to regulate Arp2/3 localization and Cofilin-dependent motility**	PMID: 25800056	Waf1 Cip1 stabilizing protein 39 (WISp39), Hsp90, Slingshot phosphatase, cofilin, Arp2/3
Abella..Way 2016	Nat Cell Biol (2016) 18:76-86	**Isoform diversity in the Arp2/3 complex determines actin filament dynamics**	PMID: 26655834	Actin, Arp2/3, cortactin
Brayford..Gunning 2016	Curr Biol (2016) 26:1312-8	**Tropomyosin Promotes Lamellipodial Persistence by Collaborating with Arp2/3 at the Leading Edge**	PMID: 27112294	Actin, Arp2/3, cofilin, slingshot (SSH1L) phosphatase, tropomyosin isoforms Tpm1.8/9
Molinie..Gautreau 2019	Cell Res (2019) 29:432-445	**Cortical branched actin determines cell cycle progression**	PMID: 30971746	Actin, Arp2/3
King..Bear 2022 (42)	J Cell Biol (2022) 221:e202111126	**Coro1B and Coro1C regulate lamellipodia dynamics and cell motility by tuning branched actin turnover**	PMID: 35657370	Actin, Arp2/3, cofilin
Kurita..Mizuno 2008	J. Biol. Chem. (2008) 283:32542–32552,	**Molecular Dissection of the Mechanisms of Substrate Recognition and F-actin-mediated Activation of Cofilin-phosphatase Slingshot-1**	PMID: 18809681	cofilin, slingshot (SSH1L) phosphatase
Cai..Bear 2007	Cell (2007) 128:915–929	**Coronin 1B Coordinates Arp2/3 Complex and Cofilin Activities at the Leading Edge**	PMID: 17350576	Actin, Arp2/3, cofilin, slingshot (SSH1L) phosphatase
Rana..Worthylake 2012	J Biol Chem (2012) 287:21836–21845	**Novel Mechanism for Negatively Regulating Rho-Kinase (ROCK) Signaling through Coronin1B Protein in Neuregulin 1 (NRG-1)-induced Tumor Cell Motility**	PMID: 22563075	Slingshot phosphatase (SSH1L), ROCK2
Priya..Michael 2016	Cell Cycle (2016) 15:3033-3041	**Coronin 1B supports RhoA signaling at cell-cell junctions through Myosin II**	PMID: 27650961	RhoA
Williams..Griendling 2012	Circ Res. (2012) 111:56-65	**Role of Coronin 1B in PDGF-Induced Migration of Vascular Smooth Muscle Cells**	PMID: 22619279	Arp2/3, PDGF
Di Giovanni..Faden 2005	J. Biol. Chem. (2005) 280:2084–2091	**In Vivo and in Vitro Characterization of Novel Neuronal Plasticity Factors Identified following Spinal Cord Injury**	PMID: 15522871	Rab13, GAP-43
Di Giovanni..Faden 2006	EMBO J. (2006) 25:4084–4096	**The tumor suppressor protein p53 is required for neurite outgrowth and axon regeneration**	PMID: 16946709	Rab13, P53
Ma..Wang 2011	Mol. Psychiatry (2011) 16:1105–1116	**Dysbindin-1, a schizophrenia-related protein, facilitates neurite outgrowth by promoting the transcriptional activity of p53**	PMID: 21502952	Dysbindin-1, P54
Föger..Bulfone-Paus 2011	J. Exp. Med. (2011) 208:1777-1787	**Differential regulation of mast cell degranulation versus cytokine secretion by the actin regulatory proteins Coronin1a and Coronin1b**	PMID: 21844203	Actin

Coronin 3

References				Specific proteins mentioned in the study
Spoerl..Hasse 2002	J. Biol. Chem (2002) 277:48858–48867	**Oligomerization, F-actin Interaction, and Membrane Association of the Ubiquitous Mammalian Coronin 3 Are Mediated by Its Carboxyl Terminus**	PMID: 12377779	Actin
Rosentreter..Clemen 2007	Exp. Cell Res. (2007) 313:878-895	**Coronin 3 involvement in F-actin-dependent processes at the cell cortex**	PMID: 17274980	Actin, Arp2/3, cofilin
Kimura..Niki 2010	Arch. Biochem. Biophys. (2010) 496:33–37	**Actin assembly controlled by GDP-Rab27a is essential for endocytosis of the insulin secretory membrane**	PMID: 20138020	Actin, Rab27a
Chan..Bear 2012	Biochem. J. (2012) 444:89–96	**Coronin 1C harbours a second actin-binding site that confers co-operative binding to F-actin**	PMID: 22364218	Actin
Tiley..Bass 2015	Small GTPases (2015) 6:36-42	**Integration of the Rac1- and actin-binding properties of Coronin-1C**	PMID: 25862165	Actin, Rac1
Striepen..Voeltz 2022	J Cell Biol (2022) 221:e202110089	**Coronin 1C restricts endosomal branched actin to organize ER contact and endosome fission**	PMID: 35802042	Actin, Arp2/3
King..Bear 2022	J Cell Biol (2022) 221:e202111126	**Coro1B and Coro1C regulate lamellipodia dynamics and cell motility by tuning branched actin turnover**	PMID: 35657370	Actin, Arp2/3, cofilin
Xavier..Clemen 2012	Sci. Rep. (2012) 2:241	**Phosphorylation of CRN2 by CK2 regulates F-actin and Arp2/3 interaction and inhibits cell migration**	PMID: 22355754	Actin, Arp2/3
Sun..Wang 2014	Cancer Biol Ther (2014) 15:1163-73	**Coronin3 regulates gastric cancer invasion and metastasis by interacting with Arp2**	PMID: 24918434	Arp2/3
Abella..Way 2016	Nat Cell Biol (2016) 18:76-86	**Isoform diversity in the Arp2/3 complex determines actin filament dynamics**	PMID: 26655834	Actin, Arp2/3, cortactin
Behrens..Clemen 2016	Eur J Cell Biol (2016) 95:239-51	**Coronin 1C-free primary mouse fibroblasts exhibit robust rearrangements in the orientation of actin filaments, microtubules and intermediate filaments**	PMID: 27178841	Actin, tubular, vimentin
Galloni..Way 2020	J. Cell Biol. (2021) 220:e202102043	**MICAL2 enhances branched actin network disassembly by oxidizing Arp3B-containing Arp2/3 complexes**	PMID: 34106209	Actin, Arp2/3, methionine monooxygenase MICAL2
Wang..Fang 2013	Oncol Rep (2013) 29:1066-72	**Knockdown of Coronin-1C disrupts Rac1 activation and impairs tumorigenic potential in hepatocellular carcinoma cells**	PMID: 23292607	Rac1
Williamson..Bass 2014	J Cell Sci 1 (2014) 27:4292-307	**Coronin-1C and RCC2 guide mesenchymal migration by trafficking Rac1 and controlling GEF exposure**	PMID: 25074804	RCC2, Rack
Tiley..Bass 2015	Small GTPases (2015) 6:36-42	**Integration of the Rac1- and actin-binding properties of Coronin-1C**	PMID: 25862165	Actin, Rac1
Williamson..Bass 2015	J Biol Chem (2015) 290:15437-15449	**Coronin-1C Protein and Caveolin Protein Provide Constitutive and Inducible Mechanisms of Rac1 Protein Trafficking**	PMID: 25925950	Rac1, caveolin
Luan..Du 2010	J Pathol 2010; 222:166–179	**Primary effusion lymphoma: genomic profiling revealed amplification of SELPLG and CORO1C encoding for proteins important for cell migration**	PMID: 20690162	selectin-P ligand (SELPLG)
Samarin..Nusrat 2010	BBRC (2010) 391:394–400	**Coronin 1C negatively regulates cell–matrix adhesion and motility of intestinal epithelial cells**	PMID: 19913511	focal adhesion kinase (FAK), paxillin
Xavier..Clemen 2012	Sci. Rep. (2012) 2:241	**Phosphorylation of CRN2 by CK2 regulates F-actin and Arp2/3 interaction and inhibits cell migration**	PMID: 22355754	Actin, Arp2/3
