# Supplementary material for: Reassessment of the roles of coronin proteins as actin effectors and in signaling
Source: PLoS Biol. 2026 Aug 18;24(8):e3003904. doi: 10.1371/journal.pbio.3003904 (PMC13485075; doi:10.1371/journal.pbio.3003904)
Supplement: Supplementary file 3 [file pbio.3003904.s014.pdf]

Figure 4 A

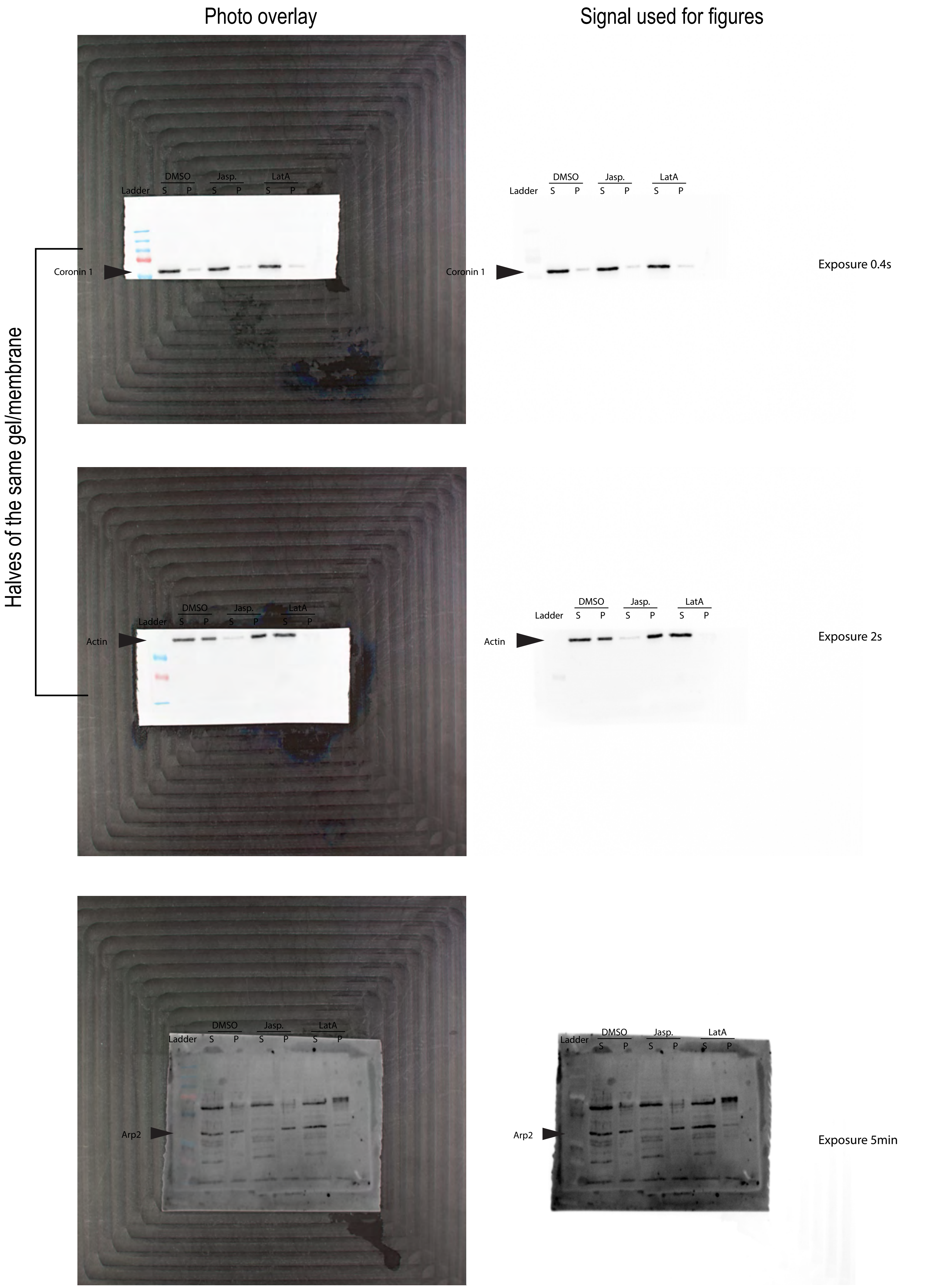
Photo overlay
Signal used for figures
Halves of the same gel/membrane
DMSO
Jasp.
LatA
Ladder
S
P
Coronin 1
Exposure 0.4s
Actin
Exposure 2s
Arp2
Exposure 5min

Figure 4 B

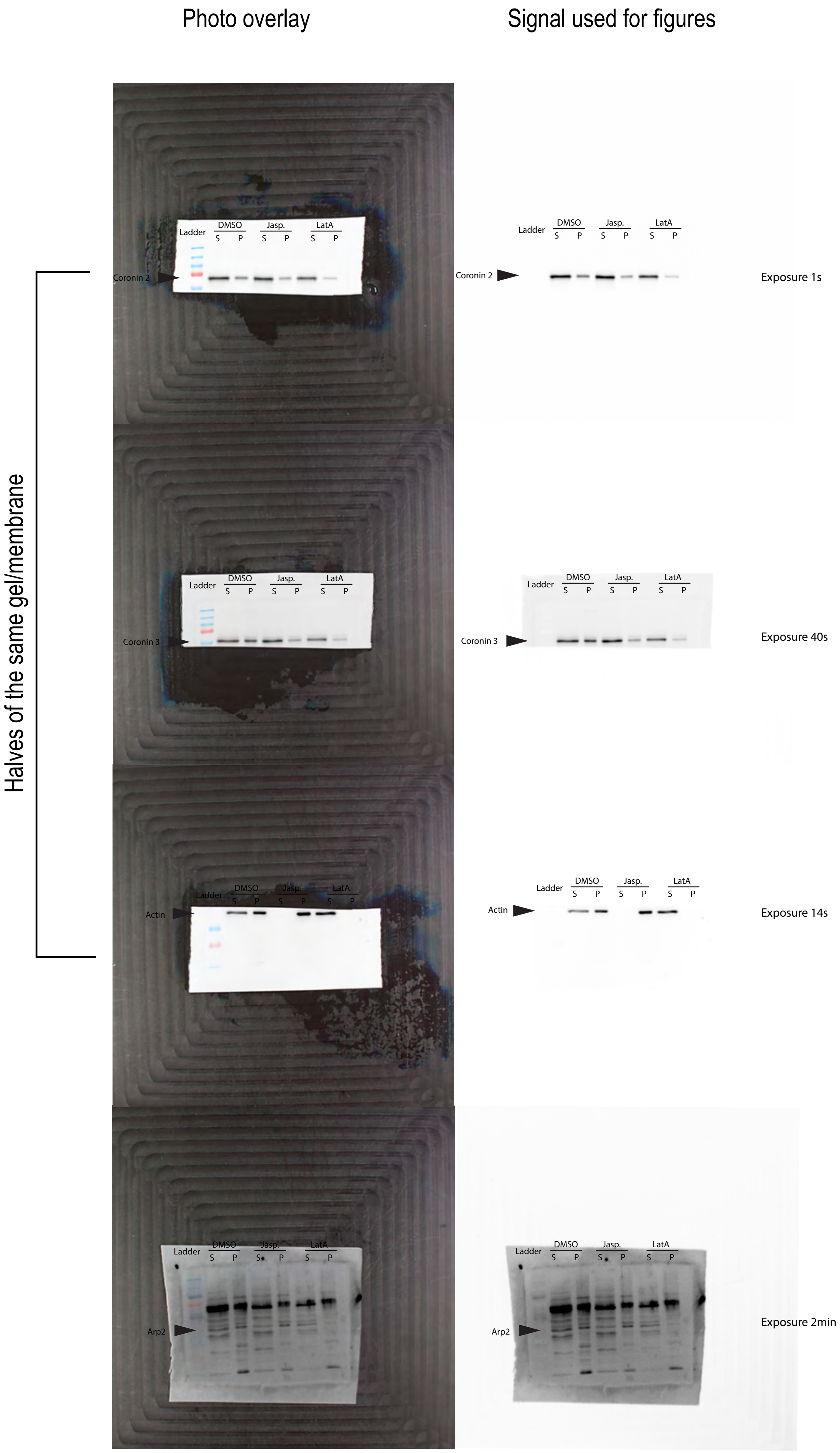

Photo overlay
Signal used for figures
Halves of the same gel/membrane
Ladder
DMSO
Jasp.
LatA
S
P
Coronin 2
Coronin 3
Actin
Arp2
Exposure 1s
Exposure 40s
Exposure 14s
Exposure 2min

Figure S1 A

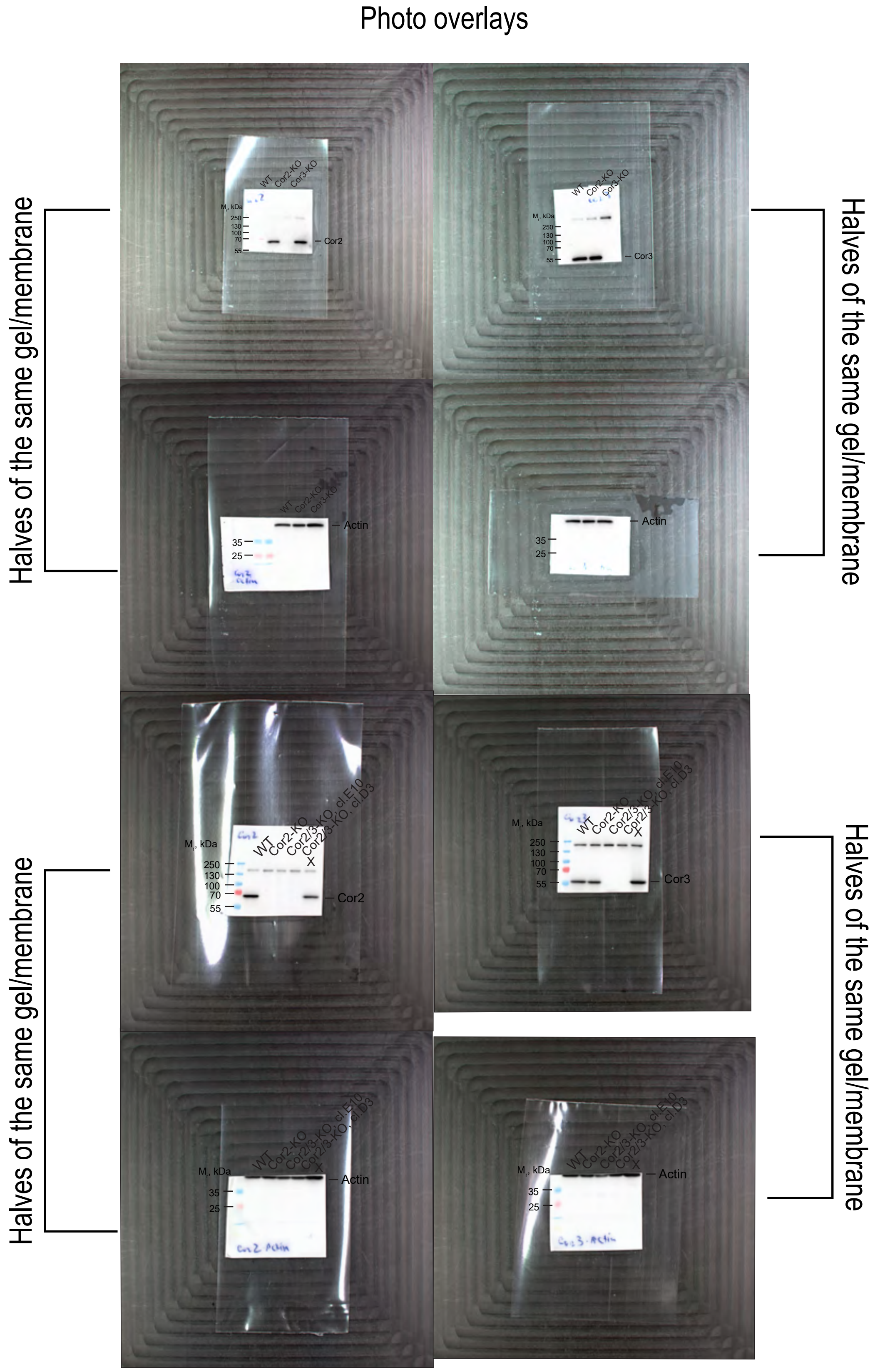

Photo overlays
Halves of the same gel/membrane
WT
Cor2-KO
Cor3-KO
Cor2
Cor3
Actin
Cor2/3-KO, cl.E10
Cor2/3-KO, cl.D3
250
130
100
70
55
35
25

Figure S5 D

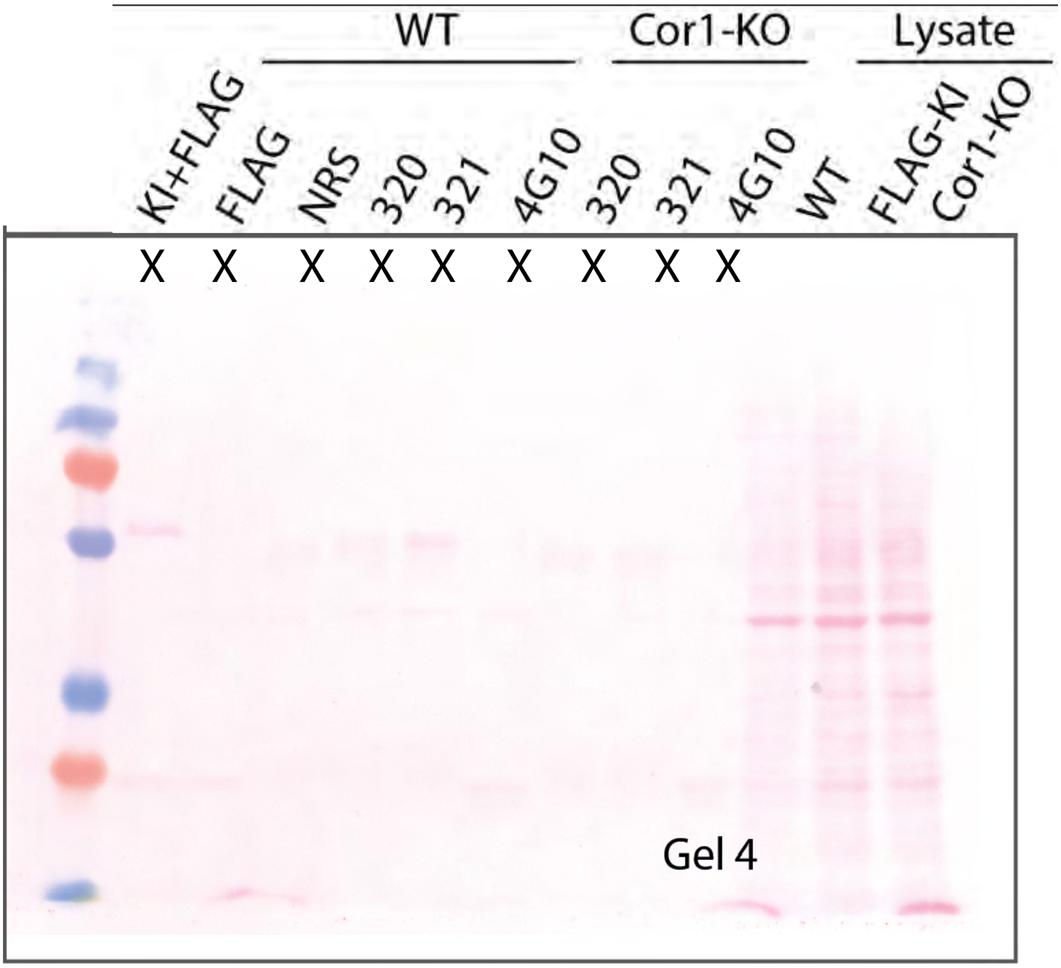
WT
Cor1-KO
Lysate
KI+FLAG
FLAG
NRS
320
321
4G10
320
321
4G10
WT
FLAG-KI
Cor1-KO
X X X X X X X X X
Gel 4

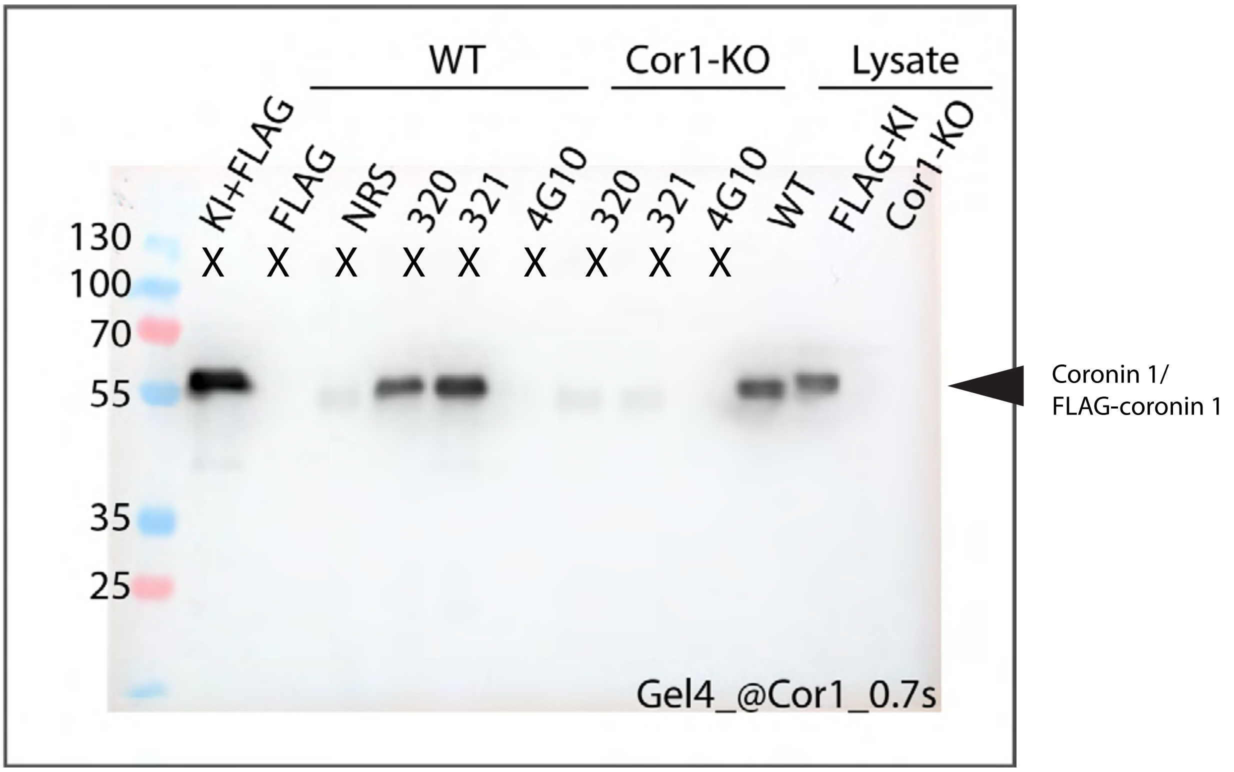
WT
Cor1-KO
Lysate
KI+FLAG
FLAG
NRS
320
321
4G10
320
321
4G10
WT
FLAG-KI
Cor1-KO
X X X X X X X X X
130
100
70
55
35
25
Gel4_@Cor1_0.7s
Coronin 1/
FLAG-coronin 1

Figure S8 A

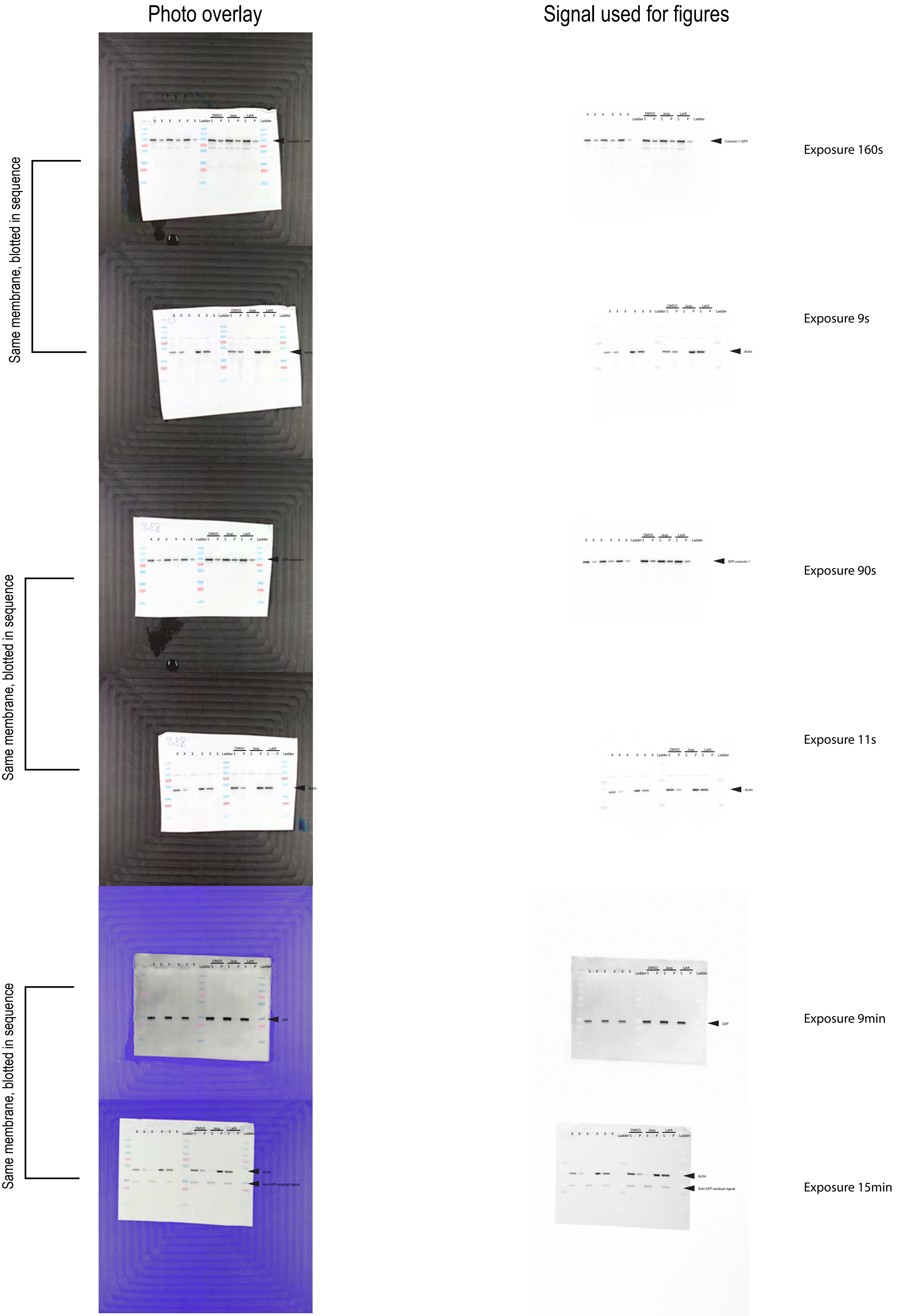
Photo overlay
Signal used for figures
Same membrane, blotted in sequence
Same membrane, blotted in sequence
Same membrane, blotted in sequence
Exposure 160s
Exposure 9s
Exposure 90s
Exposure 11s
Exposure 9min
Exposure 15min
DMSO
Jasp.
LatA
Ladder
Coronin 1-GFP
Actin
GFP-coronin 1
GFP
Anti-GFP residual signal

Figure S8 B

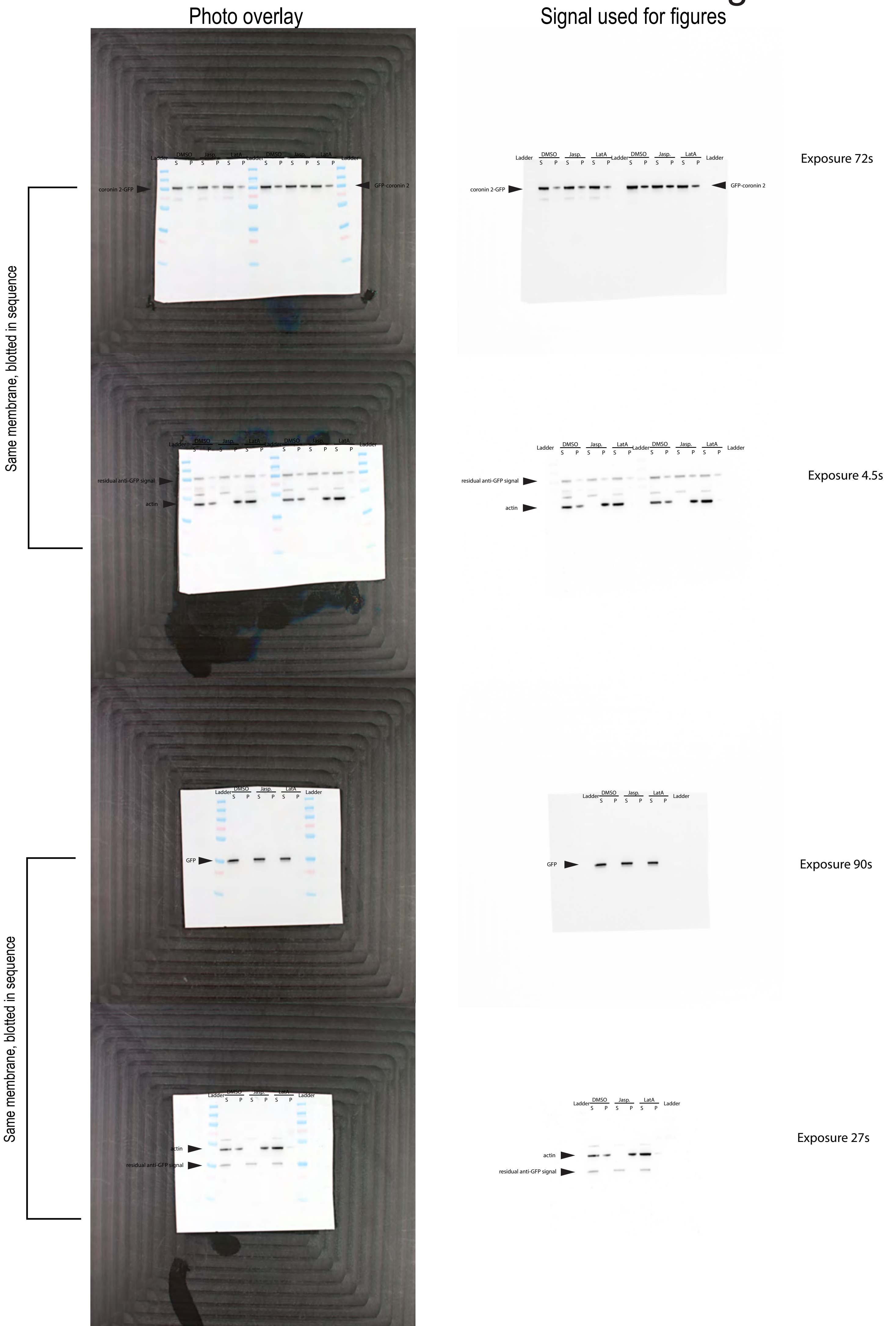

Photo overlay
Signal used for figures
Same membrane, blotted in sequence
Same membrane, blotted in sequence
Ladder
DMSO
Jasp.
LatA
S
P
coronin 2-GFP
GFP-coronin 2
Exposure 72s
residual anti-GFP signal
actin
Exposure 4.5s
GFP
Exposure 90s
Exposure 27s
